# Metabolomics and transcriptomics pathway approach reveals outcome-specific perturbations in COPD

**DOI:** 10.1038/s41598-018-35372-w

**Published:** 2018-11-20

**Authors:** Charmion I. Cruickshank-Quinn, Sean Jacobson, Grant Hughes, Roger L. Powell, Irina Petrache, Katerina Kechris, Russell Bowler, Nichole Reisdorph

**Affiliations:** 1 Department of Pharmaceutical Sciences, University of Colorado Anschutz Medical Campus, Aurora, CO 80045 United States of America; 2 Department of Biostatistics and Informatics, University of Colorado Anschutz Medical Campus, Aurora, CO 80045 United States of America; 3 Department of Medicine, National Jewish Health, Denver, CO 80206 United States of America; 4 Department of Medicine, University of Colorado Anschutz Medical Campus, Aurora, CO 80045 United States of America; 5 Flathead Valley Community College, Kalispell, MT 59901 United States of America

## Abstract

Chronic obstructive pulmonary disease (COPD) comprises multiple phenotypes such as airflow obstruction, emphysema, and frequent episodes of acute worsening of respiratory symptoms, known as exacerbations. The goal of this pilot study was to test the usefulness of unbiased metabolomics and transcriptomics approaches to delineate biological pathways associated with COPD phenotypes and outcomes. Blood was collected from 149 current or former smokers with or without COPD and separated into peripheral blood mononuclear cells (PBMC) and plasma. PBMCs and plasma were analyzed using microarray and liquid chromatography mass spectrometry, respectively. Statistically significant transcripts and compounds were mapped to pathways using IMPaLA. Results showed that glycerophospholipid metabolism was associated with worse airflow obstruction and more COPD exacerbations. Sphingolipid metabolism was associated with worse lung function outcomes and exacerbation severity requiring hospitalizations. The strongest associations between a pathway and a certain COPD outcome were: fat digestion and absorption and T cell receptor signaling with lung function outcomes; antigen processing with exacerbation frequency; arginine and proline metabolism with exacerbation severity; and oxidative phosphorylation with emphysema. Overlaying transcriptomic and metabolomics datasets across pathways enabled outcome and phenotypic differences to be determined. Findings are relevant for identifying molecular targets for animal intervention studies and early intervention markers in human cohorts.

## Introduction

Chronic obstructive pulmonary disease (COPD) is the third leading cause of death in the United States^1^. In 2015, the economic burden of COPD in the United States through 2020 was estimated to be ~$90 billion^2^, while in the European Union a recent 2017 release estimated the economic burden to be ~ €48.4 billion^3^. COPD occurs predominately in smokers; however, only 10–50% of smokers develop COPD^4^. Current therapy is focused on treating symptoms such as chronic cough and excessive sputum production, and preventing exacerbations. There are no therapies that reduce the progression of disease or mortality. Diagnosis and treatment are complicated because COPD may manifest as multiple phenotypes including airway obstruction, emphysema (destruction of alveoli), and frequent exacerbations. Identifying biomarkers and pathways that distinguish COPD phenotypes can potentially lead to more specific treatments.

In addition to clinical challenges, there remain significant gaps in understanding the pathways and mechanisms involved in the pathogenesis of COPD phenotypes. Moreover, there are inherent challenges in clinical omics-based research that make studying COPD difficult, including (1) complexity of patient stratification across multiple phenotypes^5^, (2) obtaining suitable numbers of study subjects across phenotypes, (3) lack of validated informatics tools and strategies for large clinical datasets^6,7^, and (4) limitations within the omics technological platforms. The latter includes challenges in integrating clinical metadata with multiple datasets from functional genomics, proteomics, and/or metabolomics sources; i.e. a true “systems approach”.

Some progress has been made in the fields of functional genomics and metabolomics; however, no discovery-based approach has yet resulted in validated clinical biomarkers. For example, genetic studies previously identified alpha-1 antitrypsin in 1964 as a cause of COPD^8^; however alpha-1 antitrypsin deficiency accounts for only 1% of COPD cases^9^. Most recently, proteomic approaches have identified new biomarkers such as plasma sRAGE for the presence and progression of emphysema^10,11^. Metabolomics approaches have identified potential markers of disease severity or therapeutic candidates such as purines^12^, sphingolipids^13^, glycerophospholipids^14^, and amino acids which differentiate patients with or without emphysema and/or cachexia^15^. While these omic-centric studies have added to the knowledge base, only limited information is obtained. A systems- or pathways-based approach could further pinpoint potential mechanisms by integrating datasets and thereby increasing statistical power. This approach has been used in plant and bacterial studies and has recently been applied to human disease. For example, integrating functional genomics and metabolomics identified markers associated with poor outcome in human neuroendocrine cancers^16^, determined predictors of asthma control^17^, and determined novel therapeutic targets in lung cancer^18^.

While single ‘omic studies have helped to address the knowledge gap, to date no study has integrated transcriptomics and metabolomics from a COPD cohort to determine if specific pathways are associated with individual COPD outcomes. This could be a useful strategy since functional genomics measures the association between genes, transcripts and clinical phenotype^19^ and metabolomics provides a readout of the physiological state of the human body^20^. Therefore, we used metabolomics and transcriptomics to identify markers associated with six COPD health outcomes, then mapped the significant results to pathways for each outcome. The overall goal is to identify dysregulated pathways that distinguish outcomes.

The novelty and strength of our study lies in the uniqueness of the highly phenotyped and matched samples across several stages of COPD severity. Using transcriptomics and metabolomics datasets provides information at a molecular level that is not available when only a single profiling technology is used. Determining how the molecular basis of disease phenotypes vary will improve treatment options and clinical outcomes since unique therapies can be adapted to each patient based on their individual phenotype.

## Results

### Cohort description

Clinical characteristics are provided in Table 1. Exacerbation frequency is the total number of exacerbations (0–6) in the year prior to enrollment. Exacerbation severity is the total number of severe exacerbations (0–3) in the year prior to enrollment. Age was uniform across Global Initiative for Chronic Obstructive Lung Disease (GOLD) Stages. Gender was balanced across males (n = 79) and females (n = 70). Clinical phenotypes were associated with GOLD stage, smoking pack-years, asthma, and current smoking status.

**Table 1:** Characteristics of the COPD cohort.

COPD Gold Stage	PRISm	Gold 0	Gold 1	Gold 2	Gold 3	Gold 4	p-value
Subjects	10	45	10	37	30	17	
Age (years)^‡^	60.5 (7.6)	60.4 (9.2)	63.8 (10.6)	62.4 (9.1)	68.2 (6.2)	64.3 (6)	0.0014
Gender, % (M/F) *	20%/80%	51.1%/48.9%	70%/30%	45.9%/54.1%	66.7%/33.3%	58.8%/41.2%	0.1166
BMI (kg/m^2^)^‡^	38.1 (17.9)	43.3 (27.9)	55.2 (38.3)	45.1 (23.7)	52.8 (22.3)	57.4 (30)	0.0273
Pack-Years^‡^	29.1 (6.9)	27.8 (4.9)	26.6 (4.5)	29.9 (5.9)	27.9 (6.5)	24.8 (6.7)	0.3272
BDR	0: 100%	0: 91.1%; 1: 8.9%	0: 60%; 1: 40%	0: 64.9%; 1: 29.7%	0: 70%; 1: 30%	0: 35.3%; 1: 64.7%	0.0001
Current Smoking Status (No/Yes)	50%/50%	68.9%/31.1%	100%/0%	67.6%/32.4%	86.7%/13.3%	88.2%/11.8%	0.0289
Exacerbation Frequency	0: 50%; 1: 40%; 6: 10%	0: 84.4%; 1: 11.1%; 2: 2.2%; 5: 2.2%	0: 70%; 1: 10%; 3: 20%	0: 51.4%; 1: 21.6%; 2: 16.2%; 3: 5.4%; 4: 5.4%	0: 63.3%; 1: 33.3%; 3: 3.3%	0: 52.9%; 1: 11.8%; 2: 11.8%; 3: 17.6%; 5: 5.9%	0.0011
Exacerbation Severity	0: 80%; 1: 10%; 3: 10%	0: 97.8%; 1: 2.2%	0: 100%	0: 78.4%; 1: 16.2%; 2: 2.7%; 3: 2.7%	0: 93.3%; 1: 6.7%	0: 70.6%; 1: 29.4%	0.0634
Total Severe Exacerbations/year	0.2 (0.3)	0 (0)	0 (0.1)	0.2 (0.6)	0.1 (0.3)	1.2 (3)	0.0793
Total Exacerbations/year	0.7 (0.8)	0.1 (0.3)	0.4 (0.7)	0.7 (1.1)	0.7 (0.7)	2 (3.7)	0.0153
Six-minute-walk distance^‡^	1499.5 (329.8)	1711.8 (328.4)	1509.5 (262.1)	1377.7 (448.8)	1262.6 (234.3)	850.7 (271.2)	<0.0001
Gas Trapping, %^‡^	8 (8.4)	8.3 (5.6)	25.3 (12.7)	24.2 (16.8)	49.2 (16)	59.9 (12.8)	<0.0001
Emphysema, %^‡^	0.6 (0.7)	1.4 (1.5)	9.1 (7.9)	6.3 (7)	21 (12.3)	22.1 (9.3)	<0.0001
FEV_1_/FVC^‡^	0.8 (0)	0.8 (0)	0.6 (0.1)	0.6 (0.1)	0.4 (0.1)	0.3 (0.1)	<0.0001
FEV_1_% predicted*	72.1 (5.3)	97.9 (13.4)	87.3 (10.3)	64 (8.5)	40 (5)	22.6 (5.7)	<0.0001

Values are presented as an average with standard deviation in parenthesis unless otherwise indicated as a percentage. All subjects were Non-Hispanic White. Emphysema was defined as percent of lung attenuation voxels below -950 HU; exacerbations were defined as moderate (treated with either antibiotics or corticosteroids) or severe (leading to hospitalization) in the previous year. GOLD: Global Initiative for Chronic Obstructive Lung Disease, FEV_1_: forced expiratory volume in 1 second, FVC: forced vital capacity, BDR: bronchodilator response where 0 is reversible and 1 is irreversible, PRISm: unclassified, GOLD 0: healthy, GOLD 1: mild COPD, GOLD 2: moderate COPD, GOLD 3: severe COPD, GOLD 4: very severe COPD. *For categorical covariates, a GOLD stage–specific percentage is given, along with a P value based on a *Χ*^2^ test of association. ^‡^For continuous covariates, a GOLD stage–specific mean is given, along with a P value based on an overall F-test for equality of means.

Approximately 10–15% of the current and former smoker population falls into the Preserved Ratio, Intact Spirometry (PRISm)^21^ category. Studies in COPDGene have found that these subjects are not as healthy as control subjects^21^ and it is not clear where these patients fit in the spectrum of COPD. We therefore included PRISm subjects in the transcriptomics analyses since patients within that category presented with outcomes such as exacerbations or emphysema. None of the PRISm subjects overlapped with the metabolomics dataset and were therefore not included in the metabolomics analysis.

### Individual outcome associations

**Figure 1:**
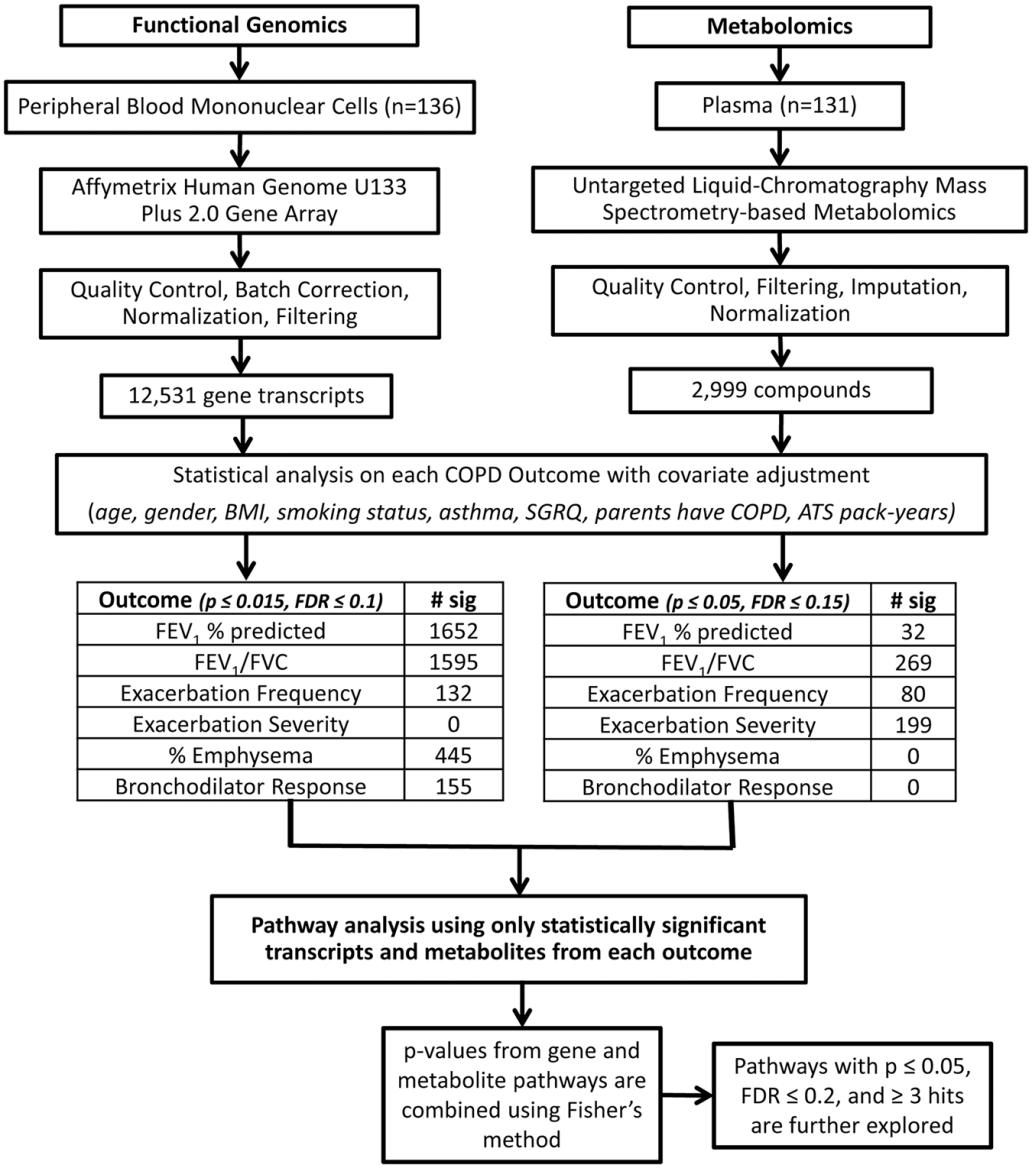
Flow chart summarizing the omics methods and analysis. Peripheral blood mononuclear cells (PBMC) (n = 136 human subjects) and matched plasma (n = 131) were prepared and analyzed using functional genomics and metabolomics, respectively. Metabolomics samples were analyzed randomly in triplicate. Regression model fitting in R resulted in a list of statistically significant transcript probes and metabolites associated with each outcome. These significant transcript probes and metabolites were mapped to pathways using IMPaLA. Pathway p-values for metabolites and transcripts were combined by a meta-analysis using Fisher’s method to obtain a single p-value and FDR for each pathway. In the outcome tables, ‘# sig’ refers to the number of statistically significant transcripts probes and metabolites. BMI: body mass index, FEV_1_: forced expiratory volume in 1 second, FVC: forced vital capacity.

**Figure 2:**
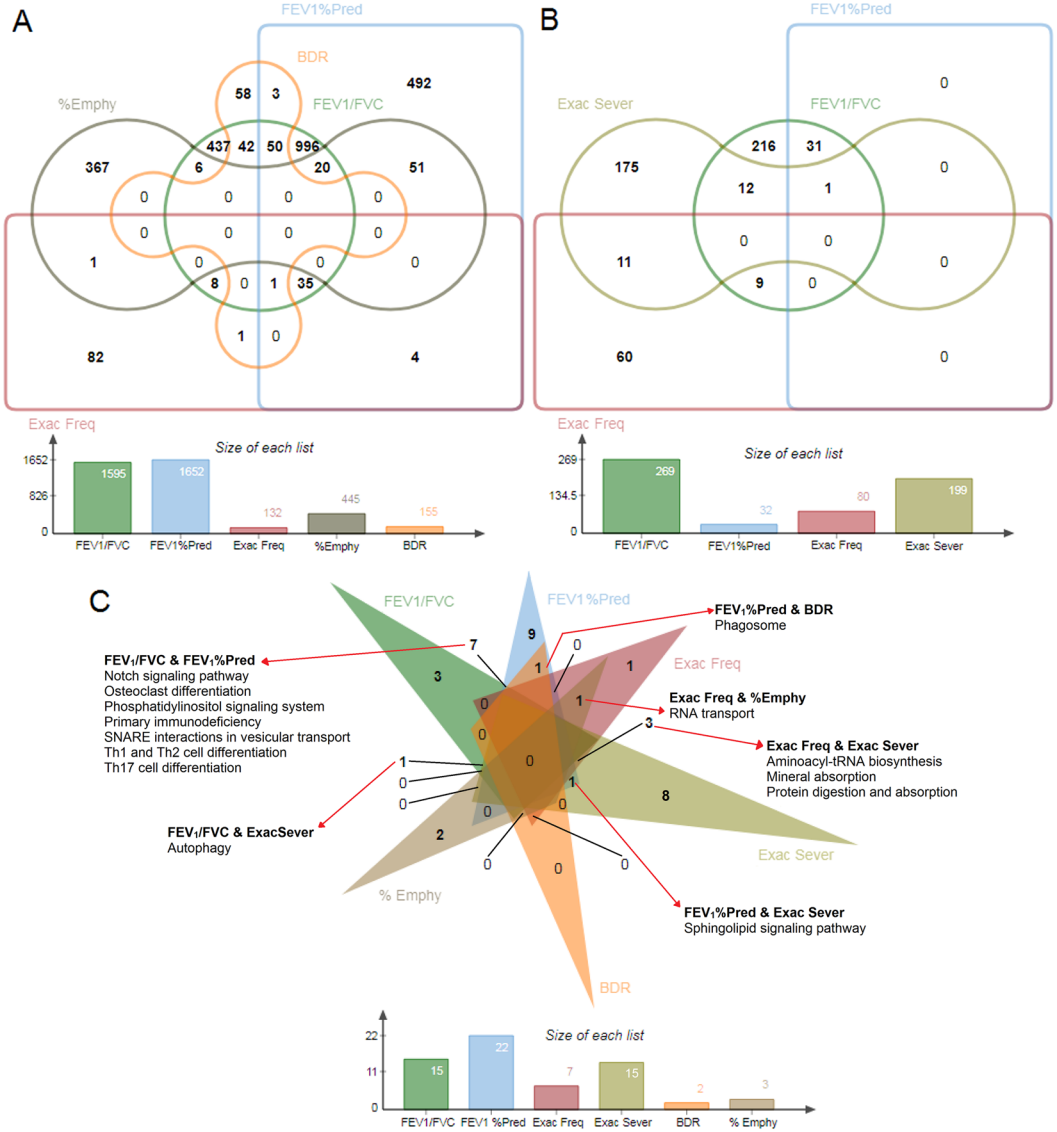
Venn diagrams. (**A**) Edwards’ Venn diagram showing the overlap of significant transcript probes (p ≤ 0.015, FDR ≤ 0.1) across COPD outcomes. **(B)** Edwards’ Venn diagram showing the overlap of statistically significant metabolites (p ≤ 0.05, FDR ≤ 0.15) across COPD outcomes. **(C)** Classic Venn diagram showing the overlap of statistically significant pathways (p ≤ 0.05, FDR ≤ 0.2, ≥3 hits). The overlapping pathways are shown in red arrows. The unique pathways are listed in Table 4. %Emphy: % emphysema, Exac Freq: exacerbation frequency, Exac Sever: exacerbation severity, BDR: bronchodilator response, FEV_1_: Forced expiratory volume in 1 second, FVC: Forced vital capacity, FEV1%Pred: FEV1% predicted.

We evaluated 12,531 transcripts and 2,999 metabolites for associations with COPD outcomes (Fig. 1). The highest number of significant transcript differences was found in FEV_1_% predicted (1,652) while no transcript differences were associated with exacerbation severity. FEV_1_/FVC had the most significant metabolites (269) while emphysema and BDR had none. There was minimal overlap in the significant transcripts (Fig. 2A) and metabolites (Fig. 2B) associated with each outcome.

The top gene transcripts for each outcome based on smallest p-value and FDR are listed in Table 2. *ACTG1*, a cytoplasmic protein found in all cell types^22^, was associated with seven transcripts, all with increased expression with increasing exacerbation frequency. For emphysema, the top gene transcripts were all associated with a decrease with worsening emphysema. These genes are associated with apoptosis (*BCL10, SUMO2*), cancer (*CCDC186, RECQL*), regulation of dietary iron absorption (*CYBRD1*), lipid binding (*OSBPL8*), protein transport (*TMCO3*), protein binding (*TOPORS*), and the mitochondria (*SLC25A43, TFAM*)^22^. The gene interactions among these significant associations were investigated, with FEV_1_/FVC having the most interactions (Supplementary Document S1).

**Table 2:** Significant associations from transcriptomics analysis.

Outcome	Transcript probe	Gene symbol	Coefficient	p-value	FDR
Bronchodilator Response	224981_at	*TMEM219*	6.141	2.15E-05	0.09541
	219150_s_at	*ADAP1*	5.766	4.44E-05	0.09541
	56256_at	*SIDT2*	3.061	7.45E-05	0.09541
	209696_at	*FBP1*	2.334	7.54E-05	0.09541
	208130_s_at	*TBXAS1*	2.836	1.03E-04	0.09541
	211071_s_at	*MLLT11*	−3.105	1.27E-04	0.09541
	221230_s_at	*ARID4B*	−3.948	1.53E-04	0.09541
	218831_s_at	*FCGRT*	3.094	1.65E-04	0.09541
	204588_s_at	*SLC7A7*	3.435	1.86E-04	0.09541
	212639_x_at	*TUBA1B*	4.649	1.86E-04	0.09541
Exacerbation Frequency	200801_x_at; 213867_x_at	*ACTB*	2.407; 1.939	<0.0001	<0.0001
	201550_x_at; 211970_x_at; 211983_x_at; 212988_x_at; 213214_x_at; 221607_x_at; 224585_x_at	*ACTG1*	3.535; 3.695; 3.095; 4.178; 2.624; 4.509; 4.536	<0.0001	<0.0001
	207988_s_at	*ARPC2*	−3.0434729	<0.0001	<0.0001
	200021_at	*CFL1*	−3.599	<0.0001	<0.0001
	204892_x_at; 213477_x_at	*EEF1A1*	−2.267; 2.220	<0.0001	<0.0001
	211956_s_at	*EIF1*	−3.919	<0.0001	<0.0001
	213932_x_at	*HLA-A*	−2.291	<0.0001	<0.0001
	209140_x_at	*HLA-B*	−3.899	<0.0001	<0.0001
	216526_x_at	*HLA-C*	−4.453	<0.0001	<0.0001
	208668_x_at	*HMGN2*	−3.989	<0.0001	<0.0001
FEV_1_/FVC	214298_x_at	*SEPT6*	−1.139	7.59E-07	0.00106
	1555889_a_at	*CRTAP*	1.070	5.02E-07	0.00106
	209397_at	*ME2*	1.585	2.42E-07	0.00106
	205005_s_at	*NMT2*	−0.649	3.71E-07	0.00106
	225314_at	*OCIAD2*	−0.900	5.92E-07	0.00106
	214857_at	*RPARP-AS1*	−0.875	4.92E-07	0.00106
	213377_x_at	*RPS12*	−4.436	5.21E-07	0.00106
	200017_at	*RPS27A*	−2.461	7.47E-07	0.00106
	201094_at	*RPS29*	−3.241	4.52E-07	0.00106
	215785_s_at	*CYFIP2*	−0.919	1.37E-06	0.00156
% Emphysema	205263_at	*BCL10*	−0.821	1.21E-05	0.01928
	227701_at	*CCDC186*	−0.861	1.67E-05	0.01928
	222453_at	*CYBRD1*	−0.527	1.69E-05	0.01928
	212582_at	*OSBPL8*	−0.823	5.09E-06	0.01928
	205091_x_at	*RECQL*	−0.860	1.32E-05	0.01928
	1557411_s_at	*SLC25A43*	−0.766	6.88E-06	0.01928
	208739_x_at	*SUMO2*	−2.160	1.57E-05	0.01928
	203177_x_at	*TFAM*	−0.850	1.69E-05	0.01928
	226050_at	*TMCO3*	−0.967	4.42E-06	0.01928
	204071_s_at	*TOPORS*	−0.791	9.90E-06	0.01928
FEV1% predicted	209397_at	*ME2*	65.244	1.83E-07	0.00229
	210607_at	*FLT3LG*	−27.897	2.47E-06	0.00543
	203569_s_at	*OFD1*	−45.307	3.46E-06	0.00543
	40446_at	*PHF1*	−46.519	2.83E-06	0.00543
	208206_s_at	*RASGRP2*	−46.273	1.94E-06	0.00543
	214857_at	*RPARP-AS1*	−36.645	1.66E-06	0.00543
	200062_s_at	*RPL30*	−212.496	3.05E-06	0.00543
	201094_at	*RPS29*	−131.468	1.95E-06	0.00543
	214298_x_at	*SEPT6*	−45.428	4.75E-06	0.00661
	203066_at	*CHST15*	23.997	7.45E-06	0.00815

Significant transcript probes (p ≤ 0.015, FDR ≤ 0.1) were matched to gene symbols for each outcome. This list comprises the ten most significant transcripts based on smallest p and FDR. FEV_1_/FVC: forced expiratory volume in 1 second/forced vital capacity. Coef: Coefficient for the indicted outcome; A positive Coef. for FEV_1_/FVC and FEV_1_% predicted indicates: associated with an increase in COPD or with COPD stage. A positive Coef. for % emphysema indicates an increase in expression with worsening emphysema. A positive Coef. for exacerbation frequency indicates an increase in expression with increasing number of exacerbations. A positive Coef. for BDR indicates an increase in expression with airflow reversible post-BDR treatment. A negative coefficient for all outcomes would be the opposite.

For the metabolomics analysis, only significant compounds that were confidently identified are shown in Table 3. A large number of amino acids were associated with exacerbation frequency while both amino acids and carbohydrates associate with exacerbation severity. Predominantly glycerophospholipids are associated with FEV_1_/FVC, and several lipid classes associate with FEV_1_% predicted. Carnitine(C14:2) was increased with increasing exacerbation frequency, acetylcarnitine* was decreased with increasing exacerbation severity, and octanoylcarnitine** was decreased with COPD for FEV_1_/FVC. The metabolite interactions for the significant associations are shown in Supplementary Document S1. Interactions were present for exacerbations and absent for lung function.

**Table 3:** Significant compounds in the metabolomics analysis.

Outcome	Compound	Class	Coef.	p-value	FDR	ID	Accession ID
Exacerbation Frequency	L-Glutamine**	Amino acid	−0.405	0.0012	0.067	MSI 1	C00064
	Pyroglutamic acid*	Amino acid	−0.607	0.0033	0.0983	MSI 1	C01879
	Tryptophan*	Amino acid	−0.845	0.0035	0.0983	MSI 1	C00078
	Tyrosine*	Amino acid	−0.372	0.0029	0.0983	MSI 1	C00082
	Carnitine (C14:2)	Carnitine	0.301	0.0012	0.067	MSI 2	HMDB13331
	Oleamide*	Fatty amide	−0.404	0.0009	0.0795	MSI 1	HMDB02117
	Dimethylallyl diphosphate	Isoprenoid phosphate	0.897	0.0013	0.067	MSI 2	C00235
	Leupeptin*	Peptide	−0.435	0.0039	0.0983	MSI 1	C01591
Exacerbation Severity	Acetylcarnitine*	Carnitine	−0.248	<0.0001	<0.0001	MSI 1	C02571
	Citrulline**	Amino acid	−0.671	<0.0001	<0.0001	MSI 1	C00327
	Creatinine**	Amino acid	−0.114	<0.0001	<0.0001	MSI 1	C00791
	L-Glutamine**	Amino acid	−0.22	0.0046	0.0149	MSI 1	C00064
	L-Norvaline**	Amino acid	−0.976	<0.0001	<0.0001	MSI 1	C01826
	Lysine*	Amino acid	0.266	<0.0001	<0.0001	MSI 1	C00047
	Cholic acid**	Bile acid	0.495	<0.0001	<0.0001	MSI 1	C00695
	7-Hydroxy-3-oxocholanoic acid	Bile acid	−0.553	<0.0001	<0.0001	MSI 2	HMDB00460
	1,6-Anhydro-β-D-glucopyranose*	Carbohydrate	−0.466	<0.0001	<0.0001	MSI 1	HMDB00640
	Alpha-D-glucose**	Carbohydrate	0.272	<0.0001	<0.0001	MSI 1	C00267
	Mannitol**	Carbohydrate	0.346	<0.0001	<0.0001	MSI 1	C00392
	2-Deoxy-D-glucose**	Fatty alcohol	−0.29	<0.0001	<0.0001	MSI 1	C00586
	3-Methyladenine**	Purine	−0.43	0.0334	0.0885	MSI 1	C00913
	Hypoxanthine**	Purine	−1.205	<0.0001	<0.0001	MSI 1	C00262
FEV1/FVC	Octanoyl-L-carnitine**	Carnitine	−0.252	0.0095	0.115	MSI 1	C02838
	Glucosaminic acid**	Carbohydrate	−0.287	0.0056	0.1049	MSI 1	C03752
	Oleamide*	Fatty amide	−0.117	0.0008	0.063	MSI 1	C19670
	DG(34:1)*	Glycerolipid	−0.14	0.0078	0.1119	MSI 1	C13861
	Eicosapentaenoyl PAF C-16*	Glycerophospholipid	0.183	0.0044	0.0933	MSI 1	132196–28–2
	LysoPC(18:1)*	Glycerophospholipid	−0.156	0.0018	0.0778	MSI 1	C03916
	PC(36:2)*	Glycerophospholipid	−0.388	0.0018	0.0778	MSI 1	C00157
	PC(36:4)*	Glycerophospholipid	−0.177	0.0098	0.1157	MSI 1	C00157
	PC(36:5)*	Glycerophospholipid	−0.611	0.0057	0.1049	MSI 1	C00157
	N-Acetylserotonin**	Indole	−0.252	0.0094	0.115	MSI 1	C00978
	Glutathione**	Peptide	−0.257	0.0034	0.0892	MSI 1	C00051
	Purine**	Purine	0.122	0.0078	0.1119	MSI 1	C15587
	Uridine**	Pyrimidine	−0.232	0.0065	0.1089	MSI 1	C00299
	Cortisone**	Steroid	−0.246	0.0047	0.0962	MSI 1	C00762
FEV_1_% predicted	cis-7-Hexadecenoic acid methyl ester*	Fatty acid	−16.395	0.0005	0.1483	MSI 1	56875-67-3
	LysoPC(16:0)	Glycerophospholipid	−8.516	0.0018	0.1483	MSI 2	C04230
	PE(P-38:2)	Glycerophospholipid	23.981	0.0018	0.1483	MSI 2	C00350
	Ceramide (d18:1/24:1)	Sphingolipid	−11.426	0.0022	0.1483	MSI 2	C00195
	Sphinganine-1-phosphate	Sphingolipid	15.122	0.0016	0.1483	MSI 2	C01120

Significant compounds (p ≤ 0.05, FDR ≤ 0.15) were identified for each outcome. Those with the highest confidence in metabolite identification and smallest FDR and p-values are shown for each outcome. Coef: Coefficient for the indicted outcome; A positive Coef. for exacerbation frequency or exacerbation severity indicates associated with increasing number of exacerbations or worsening severity of exacerbations. A positive Coef. for FEV_1_/FVC and FEV_1_% predicted indicates associated with an increase in COPD or with COPD stage. A negative coefficient for all outcomes would be the opposite. FEV_1_/FVC: forced expiratory volume in 1 second/forced vital capacity. MSI 2 indicates a database match based on accurate mass, isotope abundance and distribution, ppm ≤10, and database score ≥80 out of 100 from a spectral database. MSI 2 and *indicates confirmed by accurate mass and by matching MSMS fragments to reference standards in a spectral library; MSI 1 and **indicates confirmed by accurate mass, retention time and MSMS of purchased standards. For accession IDs, KEGG is represented, and if unavailable then denoted by CAS number or HMDB ID.

### Specific metabolites were associated with disease progression based on CT scan and FEV_1_

Clinical data from quantitative computerized tomography (CT) scans and lung function measurements from each patient was used to quantify disease progression. This clinical data was incorporated with metabolomics to identify small molecule changes associated with disease severity. There were 90 significant compounds in the emphysema progression analysis compared to 142 significant compounds in FEV_1_ progression analysis. Three compounds overlapped between the two comparisons. These were PC(36:4)* (% emphysema p = 0.0016, FEV_1_% predicted = 0.034), PE-Cer(d16:1/18:0) (% emphysema p = 0.0272, FEV_1_% predicted = 0.019), and an unannotated compound at mass 771.5351 Da (% emphysema, p = 0.0215, FEV_1_% predicted = 0.0489). PC(36:4)* and PE-Cer(d16:1/18:0) are negatively correlated with emphysema progression and positively correlated with FEV_1_ progression, while the unannotated compound was negatively correlated with both emphysema and FEV_1_ progression.

### Pathway associations

Fifteen pathways were significantly associated with FEV_1_/FVC, 22 pathways with FEV_1_% predicted, 7 with exacerbation frequency, and 15 with exacerbation severity (Table 4). The transcript expressions for the significant pathways for BDR were all increased (p ≤ 0.0013, FDR ≤ 0.0954) post-BDR treatment; for phagosome these were *ATP6V0B, ATP6V1F, FCGR2A, CYBB, HLA-DMA, RAC1, SCARB1, TUBA1B, TUBA1C*, and for lysosome these were *ATP6V0B, NPC2, NAGA, GAA, GUSB, HEXA, PSAP*.

**Table 4:** List of significant pathways.

Outcome	Dysregulated Pathways	# Hits	p-value	FDR
FEV1/FVC	Autophagy	8	0.0008	0.0250
FEV1/FVC	Fat digestion and absorption*	10	0.0001	0.0035
FEV1/FVC	Glycerolipid metabolism*	10	0.0018	0.0512
FEV1/FVC	Glycerophospholipid metabolism	18	<0.0001	<0.0001
FEV1/FVC	Hematopoietic cell lineage*	17	0.0005	0.0375
FEV1/FVC	Lysosome	25	<0.0001	0.0016
FEV1/FVC	Notch signaling pathway	10	0.0021	0.0973
FEV1/FVC	Osteoclast differentiation	23	0.0007	0.0245
FEV1/FVC	Phosphatidylinositol signaling system	14	0.0031	0.0817
FEV1/FVC	Primary immunodeficiency	9	0.0009	0.0530
FEV1/FVC	Ribosome	45	<0.0001	<0.0001
FEV1/FVC	SNARE interactions in vesicular transport	8	0.0026	0.1090
FEV1/FVC	Sphingolipid metabolism	14	<0.0001	<0.0001
FEV1/FVC	Th1 and Th2 cell differentiation	25	<0.0001	<0.0001
FEV1/FVC	Th17 cell differentiation	24	<0.0001	<0.0001
FEV_1_% predicted	Endocytosis*	39	0.0055	0.0924
FEV_1_% predicted	Fc gamma R-mediated phagocytosis*	14	0.0017	0.0386
FEV_1_% predicted	Glycerophospholipid metabolism	17	<0.0001	0.0001
FEV_1_% predicted	Hippo signaling pathway*	18	0.0041	0.0751
FEV_1_% predicted	Jak-STAT signaling pathway*	19	0.0085	0.1200
FEV_1_% predicted	Lysosome	22	0.0013	0.0318
FEV_1_% predicted	mTOR signaling pathway*	10	0.0045	0.0857
FEV_1_% predicted	Neurotrophin signaling pathway*	15	0.0070	0.1080
FEV_1_% predicted	NF-kappa B signaling pathway*	17	0.0054	0.0922
FEV_1_% predicted	Notch signaling pathway	11	0.0008	0.0247
FEV_1_% predicted	Osteoclast differentiation	28	<0.0001	0.0003
FEV_1_% predicted	Peroxisome*	14	0.0037	0.0720
FEV_1_% predicted	Phagosome	26	0.0009	0.0220
FEV_1_% predicted	Phosphatidylinositol signaling system	17	0.0019	0.0432
FEV_1_% predicted	Primary immunodeficiency	7	0.0171	0.1810
FEV_1_% predicted	Ribosome	47	<0.0001	<0.0001
FEV_1_% predicted	SNARE interactions in vesicular transport	9	0.0008	0.0247
FEV_1_% predicted	Sphingolipid metabolism	10	<0.0001	<0.0001
FEV_1_% predicted	Sphingolipid signaling pathway	19	0.0015	0.0346
FEV_1_% predicted	T cell receptor signaling pathway*	22	0.0001	0.0042
FEV_1_% predicted	Th1 and Th2 cell differentiation	29	<0.0001	<0.0001
FEV_1_% predicted	Th17 cell differentiation	29	<0.0001	<0.0001
Exacerbation Frequency	Aminoacyl-tRNA biosynthesis	3	0.0139	0.1400
Exacerbation Frequency	Antigen processing and presentation*	4	0.0021	0.1130
Exacerbation Frequency	Glycerophospholipid metabolism	3	0.0139	0.1400
Exacerbation Frequency	Mineral absorption	3	0.0183	0.1570
Exacerbation Frequency	Protein digestion and absorption	4	0.0007	0.0195
Exacerbation Frequency	Ribosome	46	<0.0001	<0.0001
Exacerbation Frequency	RNA transport	6	0.0098	0.1090
Exacerbation Severity	ABC transporters*	6	0.0004	0.0056
Exacerbation Severity	Aminoacyl-tRNA biosynthesis	5	0.0013	0.0158
Exacerbation Severity	Arginine and proline metabolism*	4	0.0002	0.0047
Exacerbation Severity	Arginine biosynthesis*	3	0.0025	0.0671
Exacerbation Severity	Autophagy	3	<0.0001	0.0004
Exacerbation Severity	Glycerophospholipid metabolism	6	<0.0001	0.0004
Exacerbation Severity	Glycine, serine and threonine metabolism*	4	0.0019	0.0156
Exacerbation Severity	Insulin resistance*	3	0.0019	0.0566
Exacerbation Severity	Mineral absorption	5	<0.0001	0.0024
Exacerbation Severity	Phenylalanine, tyrosine and tryptophan biosynthesis*	3	0.0030	0.0214
Exacerbation Severity	Protein digestion and absorption	5	0.0002	0.0118
Exacerbation Severity	Purine Metabolism*	4	0.0177	0.0834
Exacerbation Severity	Retrograde endocannabinoid signaling*	3	0.0014	0.0429
Exacerbation Severity	Sphingolipid metabolism	4	0.0001	0.0034
Exacerbation Severity	Sphingolipid signaling pathway	3	0.0008	0.0306
Bronchodilator Response	Lysosome	7	0.0001	0.0386
Bronchodilator Response	Phagosome	9	<0.0001	0.0053
% Emphysema	mRNA surveillance pathway*	8	0.0009	0.1990
% Emphysema	Oxidative phosphorylation*	11	0.0002	0.1130
% Emphysema	RNA transport	12	0.0004	0.1770

The most statistically significant pathways were filtered for KEGG Pathways, FDR ≤ 0.2, p ≤ 0.05, and ≥3 hits and are listed alphabetically. In the dataset column, the data was derived from solely statistically significant metabolites, solely statistically significant transcripts, or from both statistically significant metabolites and gene transcripts. ^#^Hits represent the number of gene transcripts and/or metabolites that are significant in the indicated pathway. *Pathways that are unique to the indicated outcome.

Three pathways were unique to FEV_1_/FVC (Fig. 2C), with fat digestion and absorption being the most significant. Nine pathways were unique to FEV_1_% predicted, with T cell receptor signaling as the most significant. There was one unique pathway associated with exacerbation frequency (antigen processing and presentation). There were eight unique pathways related to exacerbation severity, with arginine and proline metabolism as the most significant. Sphingolipid metabolism was significant in exacerbation severity with ganglioside GD3 (d18:0/23:0), lactosylceramide (d18:1/14:0), and the glycosphingolipid Fucα1–4GlcNAcβ1-3Galβ1-3GlcNAcβ1-3Galβ1-4Glcβ-Cer(d18:1/22:0) positively associated (FDR < 0.0001) with disease severity. Sphingolipid metabolism was also significant in the lung function outcomes (FDR < 0.0001), with two glycosphingolipids positively associated with FEV_1_% predicted (lung size), and three glycosphingolipids and four ceramides positively associated with FEV_1_/FVC (airflow obstruction). Emphysema was related to two unique pathways (oxidative phosphorylation and mRNA surveillance pathway). There were no unique pathways associated with BDR. Individual pathway entities are available in Supplementary Document S2.

### Transcriptome and metabolome pathways

**Figure 3:**
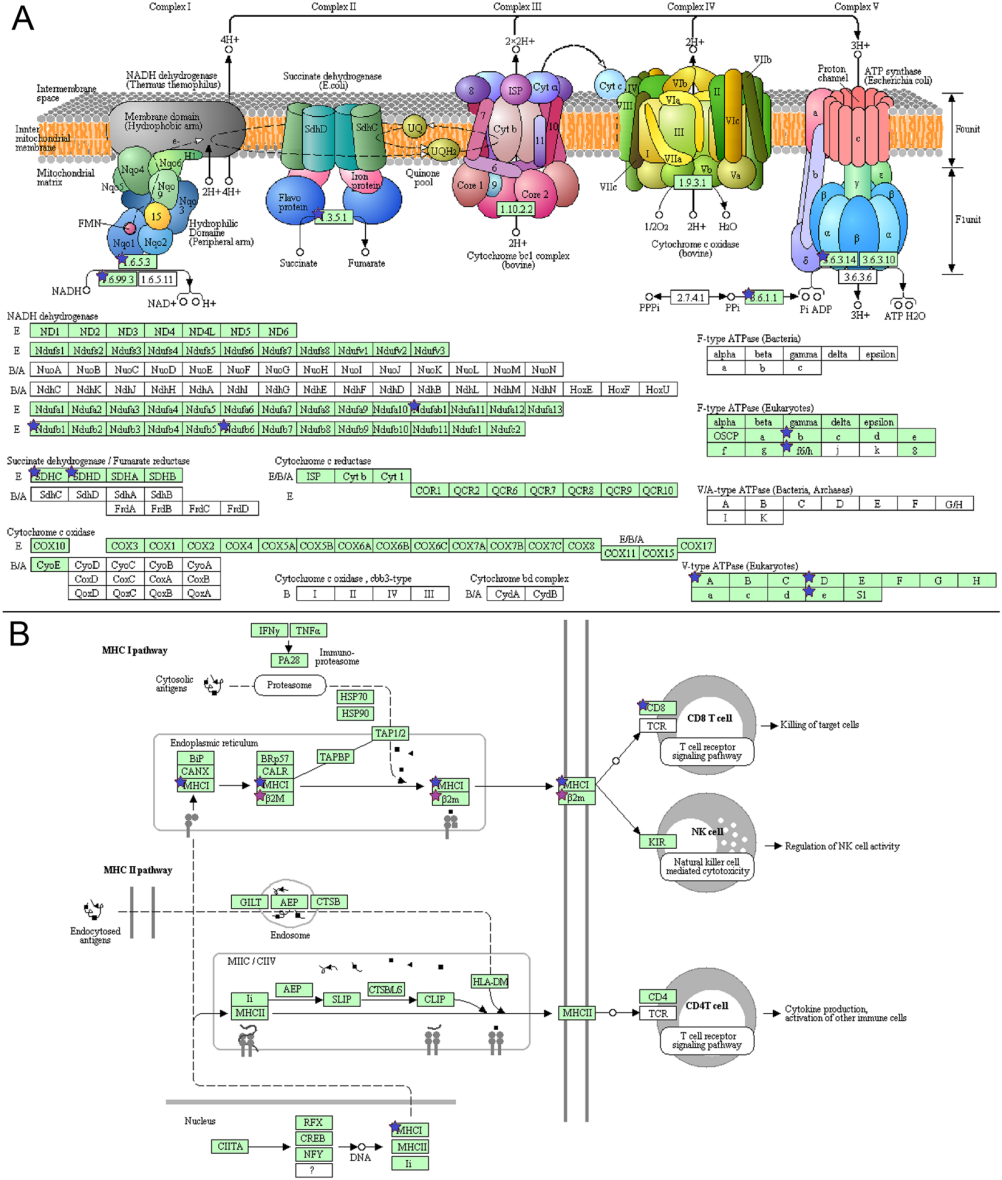
Transcriptomics pathways. (**A)** Oxidative phosphorylation in emphysema. **(B)** Antigen processing and presentation for exacerbation frequency. Blue stars indicate: associated with a decrease with worsening outcome. Purple stars represent both an increase or decrease in expression if multiple transcripts are mapped to a single gene. Pathway image is modified from KEGG^85–87^.

**Figure 4:**
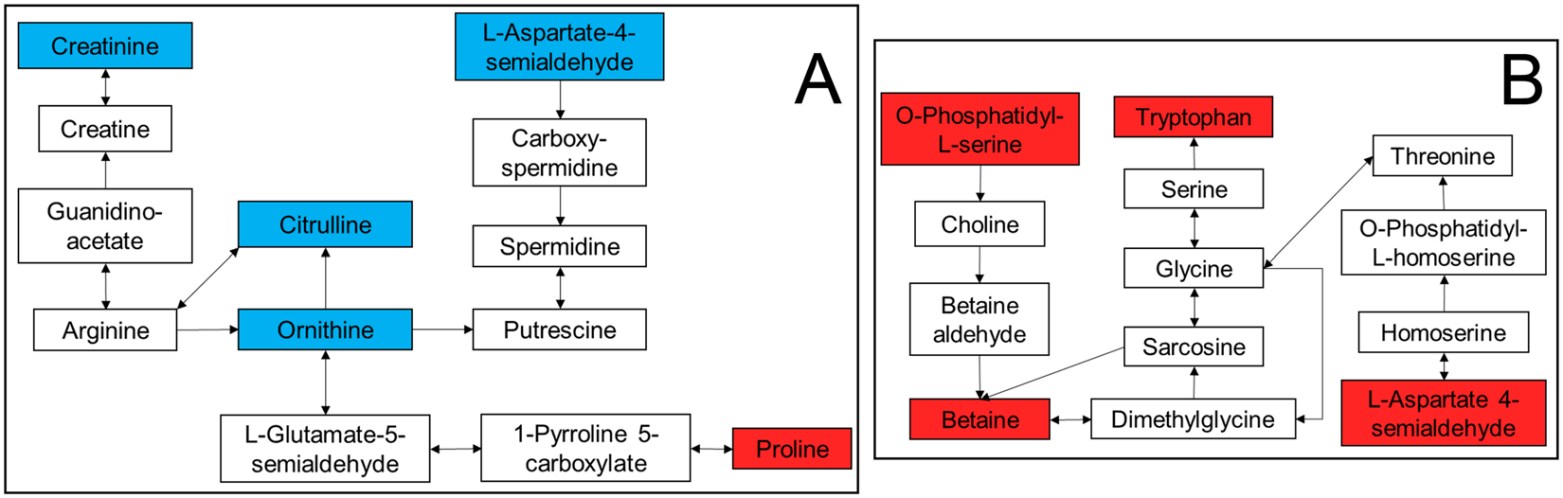
Metabolomics pathways. **(A)** Arginine and proline metabolism in exacerbation severity. **(B)** Glycine, serine and threonine metabolism in exacerbation severity. Red boxes indicate associated with an increase with worsening outcome and blue boxes indicate associated with a decrease with worsening outcome.

Oxidative phosphorylation was uniquely associated with emphysema and therefore examined more closely. All gene transcripts were decreased (Fig. 3A) and are located in complex I, II, and IV. Likewise, antigen processing and presentation (Fig. 3B) was uniquely associated with exacerbation frequency. This pathway comprises two sub-pathways (MHC I and MHC II), however only MHC I was significantly perturbed with an overall decrease. Eight unique metabolome pathways were associated with exacerbation frequency, two of which are amino acid-related metabolic pathways; arginine and proline metabolism was decreased (Fig. 4A) and glycine, serine and threonine metabolism was increased (Fig. 4B).

### Energy and cellular degradation pathways

Pathways associated with exacerbation severity and exacerbation frequency differed to some extent; therefore, HumanCyc^23^ was used to determine what was responsible for the differences. Exacerbation severity comprised eight degradation pathways and three energy pathways, while exacerbation frequency comprised three degradation pathways and no perturbed energy pathways (Supplementary Document S3).

### Integrated transcriptome/metabolome analysis

**Figure 5:**
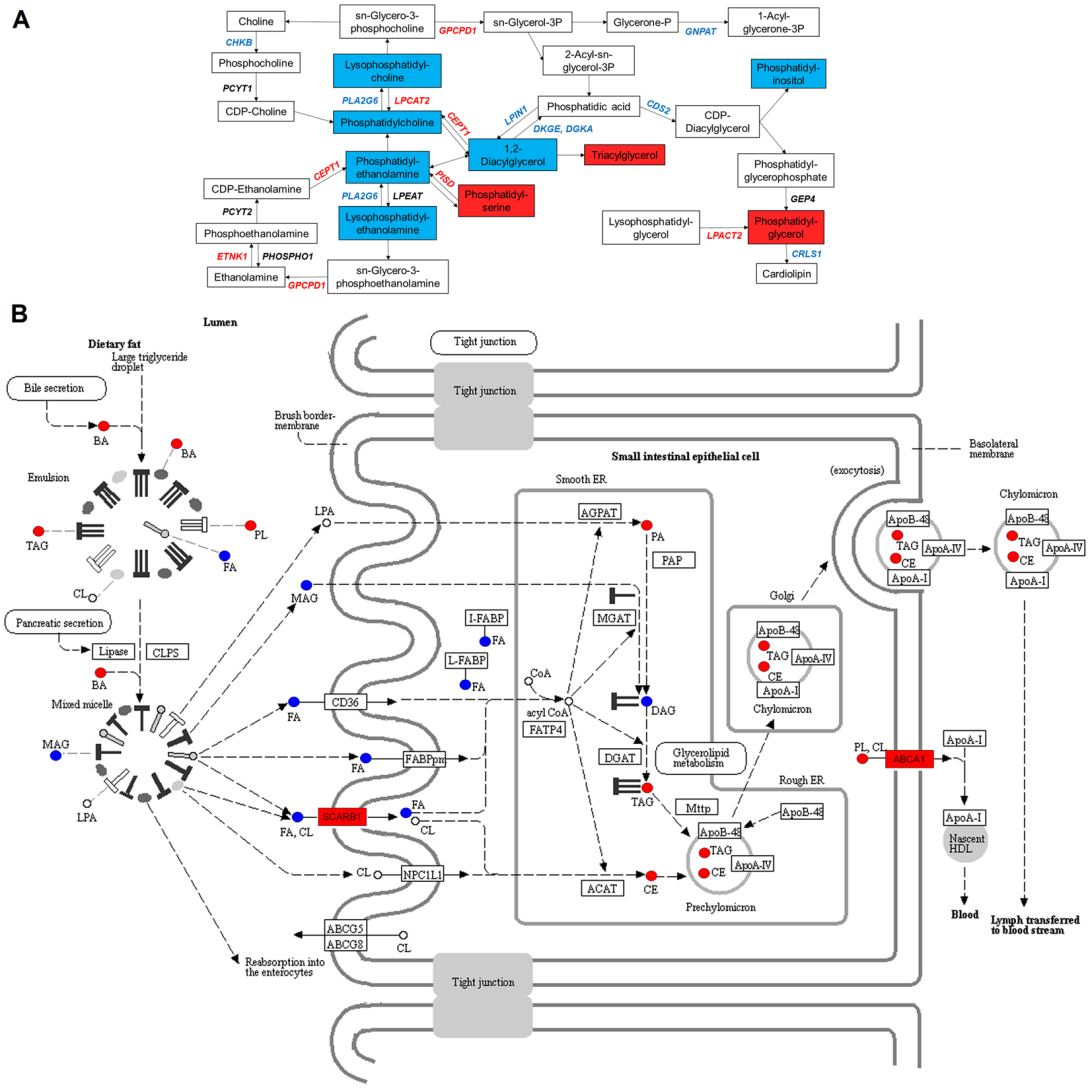
Integrated transcriptomics and metabolomics pathway diagrams. (**A)** Glycerophospholipid metabolism in FEV_1_% predicted. **(B)** Fat digestion and absorption in FEV_1_/FVC modified from KEGG^85–87^. FA: fatty acid, BA: bile acid, PA: phosphatidic acid, PL: phospholipids, MAG: monoglycerides, DAG: diglycerides, TAG: triglycerides, CE: cholesterol ester, CL: cholesterol. Red font and red boxes indicate associated with an increase with worsening disease. Blue font and blue boxes indicate associated with a decrease with worsening disease. Black font or uncolored boxes indicate no statistical significance.

Three pathways were examined to determine association between gene transcripts and metabolites. Glycerophospholipid metabolism was based on FEV_1_% predicted (Fig. 5A). As disease severity increases from GOLD 0-GOLD 4, eight gene transcripts (*CDS2, CHKB, CRLS1, DGKA, DGKE, GNPAT, LPIN1, PLA2G6*) were decreased (p ≤ 0.015, FDR ≤ 0.1) and five gene transcripts (*CEPT1, ETNK1, GPCPD1, LPCAT2, PISD*) were increased (p ≤ 0.015, FDR ≤ 0.1). Lipids that decreased with disease severity were LysoPE(16:0)*, DG(36:2)*, PC(36:2)*, PE(P-16:0), PI(41:1) (FDR ≤ 0.05), and LysoPC(20:0)* (p = 0.0018, FDR = 0.15). Conversely, PG(29:2), PS(39:7), and TG(42:0) increased (FDR ≤ 0.05) with disease severity.

Fat digestion and absorption (Fig. 5B) was uniquely significant in FEV_1_/FVC with an associated increase with disease of triglycerides, cholestrol esters, phospholipids, and bile acids, and a decrease in monoglycerides, diglycerides, and docosahexaenoic acid (DHA) with disease. The SCARB1 gene located in the brush border-membrane, and ABCA1 located in the basolateral membrane, were associated with an increase in expression in COPD subjects compared to healthy controls. The protein encoded by SCARB1 is a plasma membrane receptor for high density lipoprotein cholesterol (HDL)^24^. Lastly, glycerolipid metabolism, also uniquely significant in FEV_1_/FVC revealed an increased association with phosphatidic acid and the AGPAT9 gene that encodes it (Supplementary Document S4).

### Pathway relationships

**Figure 6:**
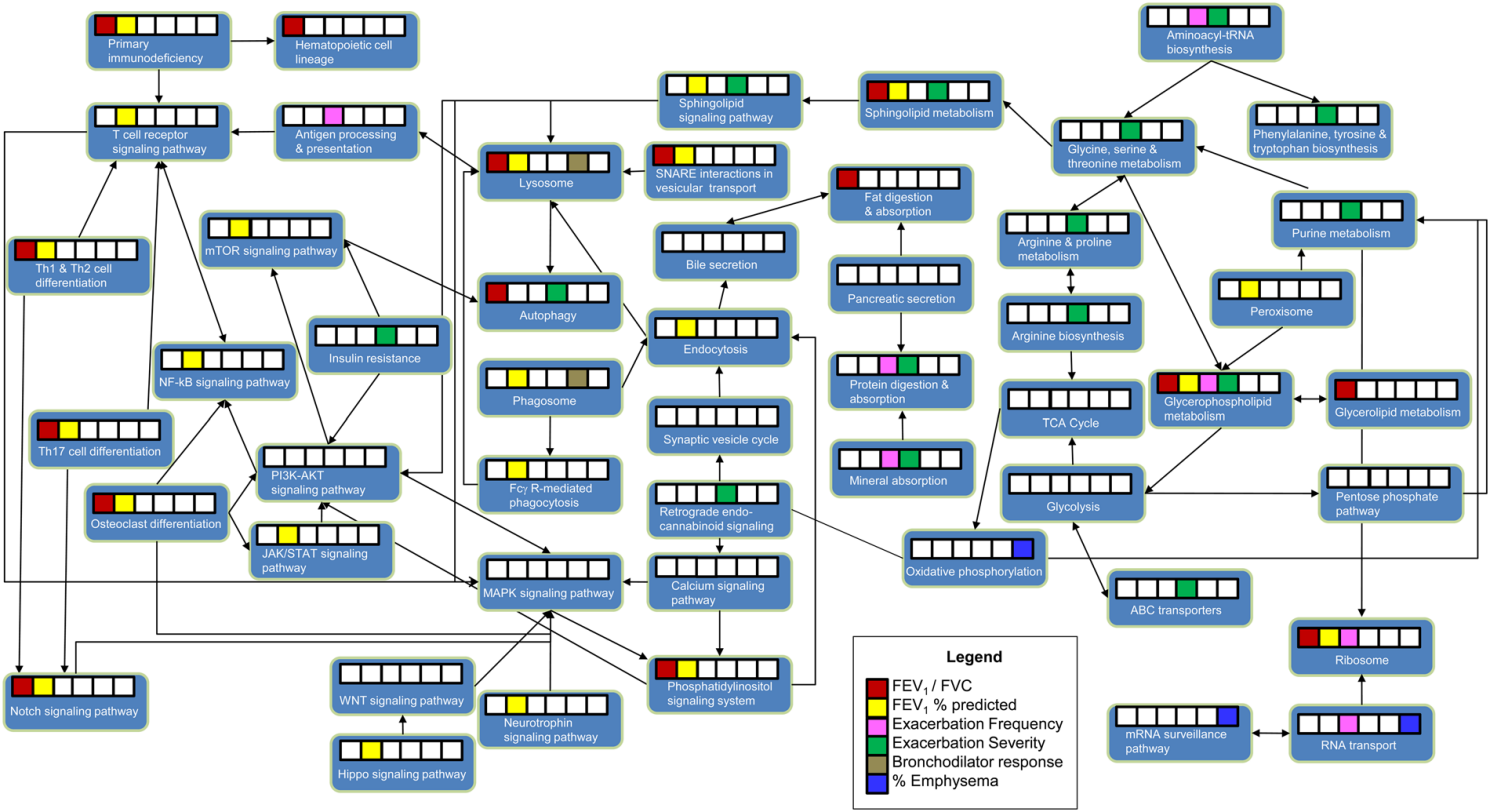
Overview of outcome perturbations and pathway relationships. The connections between the pathways were mapped using KEGG. White boxes indicate no statistical significance, while colored boxes indicate significance based on outcome as described in the Legend.

To determine whether one or multiple pathways were driving the outcome perturbations, pathways were plotted based on KEGG^25^. An integrated pathway map (Fig. 6) demonstrates that MAPK and PI3K-AKT signaling pathways, although not statistically significant, were each connected to eight pathways. In comparison, lysosome and T cell receptor signaling pathway each had six connections while purine metabolism and glycine, serine and threonine metabolism each had five connections.

## Discussion

Overlaying the significant transcripts and compounds using Venn diagrams allowed us to view the differences amongst the various outcomes in a discernable manner. For example, while a larger overlap between FEV_1_/FVC and FEV_1_% predicted might be expected, we observed minimal overlap. This is however not surprising because although these two outcomes are related, especially in a COPD cohort, their measures are different. FEV_1_/FVC ratio is a measure of obstruction and is required in the COPD diagnosis, while FEV_1_% is a measure of lung size. Therefore, a patient can have small lungs (FEV_1_%) and still have no obstruction and occasionally is the reverse (for mild COPD). Also, not surprising is the minimal overlap across all the tested outcomes since COPD is a heterogeneous disease. This approach was important in allowing us to distinguish phenotypes and outcomes.

Overall, a metabolomic signature differentiated the COPD outcomes, which was supported in the transcriptomics analysis for corresponding pathways. Both omics datasets demonstrated that specific COPD phenotypes and outcomes are associated with different transcriptome and metabolome pathways. This supports the premise that distinct COPD phenotypes have distinct mechanisms. There were also differences in the types of omics associations. For example, emphysema appears to have a larger number of gene associations compared to exacerbation severity, which has more metabolic associations. Therefore, we chose to focus the discussion on the most significant and/or unique pathways for each outcome, as a means of narrowing down the large amount of data generated from this study.

We first interpreted exacerbations as a group and observed that for exacerbation frequency, there are no significant differences in the energy pathways and very few associations in the degradation pathways. Conversely, for exacerbation severity, the energy pathways are more dysregulated and accompanied by an increase in the number of degradation pathways, particularly carbohydrate degradation, nucleoside degradation, fatty acid degradation, and amino acid degradation. This is supported by the energy association in degradation pathways; carbohydrate breakdown provides a source of cellular energy for other syntheses such as protein transport, nucleosides act as signaling molecules or provide chemical energy to cells, and fatty acids store energy in the cell^26^. We then observed that sphingolipid metabolism was significant in exacerbation severity. In our previous studies, we observed that trihexosylceramides, a subclass of sphingolipids, were positively associated with severe exacerbations^13^.

Interestingly we also observed for exacerbations that while antigen processing and presentation was unique to exacerbation frequency, only the MHC I pathway (leading to CD8 T cell activation) and not the MHC II pathway (leading to CD4 T cell activation) is affected. The activation of CD4 T cells is beneficial to sustaining memory of CD8 T cells following acute infection^27^. Since COPD exacerbations are associated with viral^28,29^ and bacterial^30,31^ infections, the perturbation of CD8 but not CD4 in our study suggests that a crucial connection is not being made, and this may predispose patients to frequent exacerbations as their immune system is weakened. An alternative explanation is that CD4 T cells were activated and CD8 T cells are now destroying the viral or bacterial pathogen in the more frequent exacerbations. Interestingly, an outcome-pathway shift is observed where the perturbed genes within the MHC I pathway for exacerbation frequency end at the T cell receptor signaling pathway which is uniquely perturbed in FEV_1_% predicted.

We then examined the emphysema phenotype. Oxidative phosphorylation was unique to and significant in the emphysema outcome. Cigarette smoking (CS) is a major cause of emphysematous COPD. CS is known to produce reactive oxygen species (ROS). Excessive production of ROS can lead to oxidative damage, leading to cell apoptosis^32^. In a recent study, exposure of human lung fibroblasts to various doses of nicotine and e-cigarette condensate inhibited myofibroblast differentiation and inhibited oxidative phosphorylation complex III^33^. Therefore, in spite of adjusting for smoking status, our results are not unexpected as our cohort is comprised of current and former smokers.

Next, we focused on the BDR outcome. Unlike the other outcomes, BDR was associated with two cytoplasmic pathways, lysosome and phagosome. Lysosomes break down proteins, nucleic acids, carbohydrates and lipids^26^. Phagosomes fuse with lysosomes allowing the ingested material to be digested; some of the material is recycled to the plasma membrane^26^. The perturbed transcripts in these pathways showed increased expression and may serve as markers of airflow reversibility.

We then examined FEV_1_% predicted outcome. Glycerophospholipid metabolism, although significant in many of the other outcomes, was highly significant in this lung function outcome. These lipids play a role in respiratory infections^34^ and in asthma and COPD^35^ since they constitute lung surfactant^36–38^. Surfactant, a complex combination of proteins and ~90% lipids^39^, is secreted by the airway epithelial cells into the airspace to reduce surface tension and provides a barrier to pathogens. In the FEV_1_% predicted outcome, the lipids classes LysoPE and PG, decreased and increased respectively, and have been previously suggested as markers of lung remodeling^40^. Their associated genes PLA2G6 and LPCAT2 were also decreased and increased respectively and could serve as targets in future studies to repair disrupted surfactant composition.

Sphingolipids were also highly associated with this outcome and FEV_1_/FVC. This is congruent with our recent publications that discuss in depth the significance of sphingolipids to COPD pathogenesis, including signaling related to structural cell fate, innate immune responses, and lymphocyte trafficking^41,42^. Other pathways uniquely and significantly associated with the FEV_1_/FVC was involved in fat digestion and absorption, typicaly reflecting perturbations between the intestine and blood. SCARB1 may be driving the cholesterol (CL) and cholesterol ester (CE) transport and regulation while the phospholipids (PL) may be driving ABCA1. Since bile acids and triglycerides were increased with disease, and the omega-3 fatty acid DHA was decreased with disease, this pathway suggests a diet or nutrition component associated with disease. This is supported by a recent publication on a cohort of 34,739 women where long-term consumption of fruits was inversely assocated with COPD incidence^43^. Diet has been suggested to play a role as a risk factor for many chronic diseases including COPD^44,45^, and suggestions have been made on the role of nutritional supplement therapy in the treatment of COPD^46^.

Finally, we attempted to deconstruct the results into a pathway map where relations could be observed among the outcomes and pathways. Based on the overview pathway map, MAPK and PI3K-AKT signaling pathways appeared to be the unifying links among many of the perturbed pathways. In a recent study exposing human bronchial epithelial cells to urban particulate matter, airway inflammation was triggered via activation of MAPK and NF-κB signaling pathways^47^. Although MAPK signaling was not significant in our study, NF-κB signaling was significant in FEV_1_% predicted. MAPK and PI3K-AKT signaling pathways may therefore be the driving force for downstream events. Researchers have already begun exploring these targets^48–51^.

A limitation of our study is the use of plasma and PBMCs as these may not directly translate to the lung environment. However, our previous studies, both published and unpublished, have observed a high degree of congruency for compounds in lung lavage fluid, lung tissue and plasma in both human and animal studies^52,53^. Also non-fasting samples may affect results as food intake affects the plasma metabolite profile^54^, We therefore used stringent statistics to filter out many of the diet-associated compounds. Lastly, patients in this study were taking medications, which is unavoidable with this type of cohort. However, we did our best to filter those medications from the final dataset prior to analysis. Future work would incorporate lavage fluid and increase sample numbers to gain more power for discriminating metabolite-transcript-phenotype differences. Targeted quantitative analysis of pathway-specific molecules could provide increased power and more quantitative results. Although these results need to be validated in a larger cohort, they point to therapeutic targets to be tested in animal experiments, and the most significant species from each outcome could serve as non-invasive markers of disease outcomes.

Despite these limitations, we were able to determine unique pathways that can be leveraged for treatment targets. For example, oxidative phosphorylation was decreased with worsening emphysema. It is perturbed due to cigarette smoke exposure^33^; cigarette smoking is a major cause of emphysema and produces reactive oxygen species, leading to oxidative damage. Interventions such as rotenone, antimycin A, or oligomycin can be used to inhibit oxidative phosphorylation^33,55,56^, while other small molecule interventions can be used to activate the pathway. Autophagy was highly significant for FEV_1_/FVC and can be inhibited using, 3-methyladenine^57,58^. Another pathway that can be leveraged is antigen processing and presentation. This pathway, decreased in exacerbation frequency and is dysregulated in a rat model of COPD^59^. Therefore, activating these pathways using either small molecules or gene interventions in animal models could be beneficial in developing phenotype-specific COPD treatments to reduce or halt lung damage or viral load. These pathway interventions in mouse models have the potential to lead to phenotype-specific treatment targets. Other follow up studies could include targeted analysis in COPD or smoking cohorts such as COSYCONET^60^, Birmingham^61^, SPIROMICS^62^, or KORA^63,64^.

## Conclusion

Our pilot study has shown that using a pathway enrichment approach to analyze transcriptomics and metabolomics data from matched samples, identified perturbed biological pathways associated with COPD outcomes and identified pathways that distinguish between outcomes. In addition, our results show that the various phenotypes and outcomes have distinct pathways associated with each one, in spite of COPD being a heterogenous disease. Investigators may choose to select the strongest pathways (those with low p-values) for follow up analyses using either mouse intervention studies via knockouts, mouse treatment studies, or using targeted analysis in other COPD or smoking cohorts. The long term clinical applicability of this work would be in the use of blood-based markers where a simple blood panel could identify patient outcomes and indicate patient-targeted treatment. This work could help in the management of COPD.

## Methods

### Ethics statement

All methods were performed in accordance with the relevant guidelines and regulations. Human subjects were from the COPDGene cohort^65^ which is a National Institute of Health–sponsored multicenter study of the genetic epidemiology of COPD. COPDGene was approved by the institutional review board at each participating center; all subjects were enrolled from January 2008 to April 2011 and provided written informed consent. The current analysis was approved by the National Jewish Health Institutional Review Board.

### Study population

The COPDGene study enrolled 10,192 non-Hispanic White and Black individuals, aged 45–80 years old with at least a 10 pack-year history of smoking, who had not had an exacerbation of COPD for at least the previous 30 days. Additional information on the COPDGene study and the collection of clinical data has been previously described^65^. There were ten Preserved Ratio, Intact Spirometry (PRISm)^21^ subjects included in the transcriptomics analysis since these subjects presented with exacerbations or emphysema but are considered an unclassified. Patients who were pregnant or with co-morbidities such as a history of other lung disease (except asthma), active cancer under treatment, and other cardiac hospitalizations, were excluded from the study^65^. Non-fasting blood was collected from a random sampling of subjects (n = 149) from a single clinical center (National Jewish Health) that represented the entire cohort and used for omics analysis. Peripheral blood mononuclear cells (PBMC) from 136 subjects were used for functional genomics and fresh frozen plasma from 131 subjects were used for metabolomics, with an overlap of 118 subjects for both omics technologies. The cohort characteristics are shown in Table 1.

### Clinical data collection and definitions of COPD outcomes

The clinical data included forced expiratory volume in 1 second/forced vital capacity (FEV_1_/FVC), FEV_1_ percent (%) predicted, percent (%) emphysema on CT scan^10^, exacerbation frequency, exacerbation severity, and bronchodilator response (BDR). COPD was defined as post bronchodilator ratio of FEV_1_/FVC < 0.70. Current or former smokers without spirometry evidence of airflow obstruction (FEV_1_/FVC ≥ 0.70) were classified as controls^66^. Subjects with without airflow obstruction but FEV_1_ < 80% of predicted where classified as (PRISm)^21^. Emphysema was measured using quantitative high resolution CT as previously described^67^ and quantified as the percent of lung attenuation lung voxels (LAA) ≤−950 HU on the inspiratory images for the whole lung. Emphysema was further categorized as none (LAA ≤ 5%), mild (5% < LAA ≤ 10%), moderate (10% < LAA ≤ 20%), or severe (20% < LAA). Exacerbations (flare ups) of COPD are characterized by acutely worse cough, sputum, and dyspnea. Only moderate exacerbations (treated by corticosteroids or antibiotics) or severe exacerbations (causing an admission to the hospital) were evaluated. Patients had no exacerbations for at least 30 days prior to sample collection. BDR determined the reversibility of airway obstruction by measuring FEV_1_ before and after the administration of bronchodilator medication. A significant response was defined as an increase in FEV_1_ of 12% and 200 mL increase^68^.

### Sample collection

Blood was drawn into a BD Vacutainer Cell Preparation Tube for peripheral blood mononuclear cells (PBMC) and plasma as previously described^65^. PBMCs were used for gene expression profiling using the Affymetrix Human Genome U133 plus 2.0 Gene Array, (GEO accession number GSE42057)^69^. Plasma was used for untargeted liquid-chromatography mass spectrometry (LC-MS)-based metabolomics.

### Chemicals, standards and reagents

All solvents were LC-MS grade. Water and isopropyl alcohol were purchased from Honeywell Burdick & Jackson (Muskegon, Michigan); chloroform, acetonitrile, methanol, acetic acid, low retention microcentrifuge tubes, serological pipettes were purchased from Fisher Scientific (Fair Lawn, New Jersey); plastic pipette tips were purchased from USA Scientific (Orlando, Florida); methyl tert-butyl ether was purchased from J.T. Baker (Central City, Pennsylvania); internal standards were purchased from Avanti Polar lipids Inc. and Sigma Aldrich (St. Louis, MO); pyrex glass culture tubes were purchased from Corning Incorporated (Corning, New York).

### Sample preparation

Blood samples for functional genomics analysis were prepared as previously described^69^. Briefly, blood was collected from subjects, processed within 30 minutes and peripheral blood mononuclear cells (PBMC) were isolated from the supernatant for RNA isolation as previously described^69^. Fresh frozen plasma was isolated using P100 tubes as previously described^70^ and stored at −80 °C until sample preparation for untargeted liquid-chromatography mass spectrometry (LC-MS)-based metabolomics. For LC-MS, 100 µL of each sample underwent protein precipitation using methanol, followed by liquid-liquid extraction using methyl-tert butyl ether as previously described^71,72^ to obtain an aqueous fraction and a lipid fraction. All samples were prepared randomly to avoid batch effects from phenotype, gender, or GOLD stage.

### Liquid chromatography mass spectrometry

The samples from the lipid fraction were analyzed randomly and each sample vial was run randomly in triplicate using an Agilent 1290 series pump with an Agilent Zorbax Rapid Resolution HD (RRHD) SB-C18, 1.8 micron (2.1 × 100 mm) analytical column and an Agilent Zorbax SB-C18, 1.8 micron (2.1 × 5 mm) guard column. The autosampler tray temperature was set at 4 °C, column temperature was set at 60 °C, and the sample injection volume was 4 µL. The flow rate was 0.7 mL/min with the following mobile phases: mobile phase A was water with 0.1% formic acid, and mobile phase B was 60:36:4 isopropyl alcohol:acetonitrile:water with 0.1% formic acid. Gradient elution was as follows: 0–0.5 minutes 30–70% B, 0.5–7.42 minutes 70–100% B, 7.42–9.9 minutes 100% B, 9.9–10.0 minutes 100–30% B, followed by column re-equilibration. The lipid fraction mass spectrometry conditions were as follows: Agilent 6210 Time-of-Flight mass spectrometer (TOF-MS) in positive ionization mode with dual electrospray (ESI) source, mass range 60–1600 m/z, scan rate 2.03, gas temperature 300 °C, gas flow 12.0 L/min, nebulizer 30 psi, skimmer 60 V, capillary voltage 4000 V, fragmentor 120 V, reference masses 121.050873 and 922.009798 (Agilent reference mix).

The samples from the aqueous fraction were analyzed randomly with each sample vial run randomly in triplicate using an Agilent 1200 series pump using a Phenomenex Kinetex HILIC, 2.6 µm, 100 Å (2.1 × 50 mm) analytical column and an Agilent Zorbax Eclipse Plus-C8 5 µm (2.1 × 12.5 mm) narrow bore guard column. The autosampler tray temperature was set at 4 °C, column temperature was set at 20 °C, and the sample injection volume was 1 µL. The flow rate of 0.6 mL/min with the following mobile phases: mobile phase A was 50% ACN with pH 5.8 ammonium acetate, and mobile phase B was 90% ACN with pH 5.8 ammonium acetate. Gradient elution was as follows: 0.2 minutes 100% B, 0.2–2.1 minutes 100–90% B, 2.1–8.6 minutes 90–50% B, 8.6–8.7 minutes 50–0% B, 8.7–14.7 minutes 0% B, 14.7–14.8 minutes 0–100% B, followed by column re-equilibration. The aqueous fraction mass spectrometry conditions were as follows: Agilent 6520 Quadrupole Time-of-Flight mass spectrometer (Q-TOF-MS) in positive ionization mode with ESI source, mass range 50–1700 m/z, scan rate 2.21, gas temperature 300 °C, gas flow 10.0 L/min, nebulizer 30 psi, skimmer 60 V, capillary voltage 4000 V, fragmentor 120 V, reference masses 121.050873 and 922.009798 (Agilent reference mix).

### Tandem mass spectrometry (MSMS)

The HILIC and C18 chromatographic methods discussed above were replicated for LC-MS/MS analysis using 10, 20, and 40 eV collision energies on a 6520 Q-TOF-MS (Agilent) with a 500 ms/spectra acquisition time, 4 m/z isolation width, and 1 minute delta retention time.

### Metabolomics quality control

Samples were randomly selected from 2000 subjects from the COPDGene cohort comprising both healthy subjects and COPD patients to create aliquots of pooled QC plasma samples. At least 20 of the 131 subjects in this study were included in the pooled sample. Samples were prepared as described above and pooled for use as quality control (QC) samples to monitor instrument reproducibility across multiple days. These pooled QC samples were injected after every five samples, followed by a solvent blank. Total ion chromatograms of all samples were evaluated for retention time reproducibility and intensity overlap. Instrument QC samples were analyzed to ensure that peak areas of spiked internal standards in the plasma samples were reproducible with coefficient of variations ≤10%.

### Metabolomics spectral peaks extraction

A final list of compounds was obtained by collapsing features (e.g. ions, adducts) into compounds as follows: Spectral peaks were extracted using the following parameters in MassHunter software B.07 (Agilent Technologies): Find by Molecular Feature algorithm, single charge, proton, sodium, potassium, ammonium adducts in positive ionization mode. Data were imported into Mass Profiler Professional software 14.5 (MPP, Agilent Technologies) for mass (15 ppm) and retention time alignment (0.2 minutes). Data from sample preparation blanks and instrument blanks were background subtracted to eliminate noise from contaminants. Because LC-MS data can result in missing values, data was further processed using the ‘Find by Formula’ algorithm parameters (+H, +Na, +K, +NH_4_ adducts for positive ionization mode, charge states limited to 2, and absolute height >3000 counts). The ‘Find by Formula’ algorithm merged multiple features such as ions, adducts and dimers into a single compound. The final data set was then re-imported into MPP for metabolite annotation.

### Metabolite annotation and identification

For the first round of identification, ID Browser within the Mass Profiler Professional software v14.5 was used to annotate metabolites with putative identifications. This software utilizes an in-house database comprising data from METabolite LINk (METLIN), Human Metabolome Database (HMDB), Kyoto Encyclopedia of Genes and Genomes (KEGG) and Lipid Maps and applies isotope ratios, accurate mass, chemical formulas, and scores to provide preliminary identifications. A database score ≥70 out of a possible 100 was considered acceptable for annotation confidence. Chosen elements for molecular formula generation were: C, H, N, O, S, and P. An error window of ≤10 ppm was used with a neutral mass range up to 2500 Da and positive ions selected as H, Na, K, and NH_4_. The database identifications were limited to the top 10 best matches based on score, and charge state was limited to a maximum of 2. There were 1,633 out of 2,999 compounds that were putatively matched to a name in a database.

For improved confidence, only the statistically significant compounds were fragmented as described in the Tandem MS section above, and searched using either an in-house mass, retention time, and MSMS library built from purchased standards, or their MSMS fragments were matched to reference standards from the NIST14 and NIST17 MSMS spectral library^73^. For compounds where their fragments were not present in a reference spectral library, MetFrag^74,75^ was used for *in silico* fragmentation. A list of the confirmed and putative IDs is available in Supplementary Documents S5 and S6.

### Reporting level of confidence

Within the manuscript and in Table 3, the symbol * indicates that the compound name was confirmed by accurate mass (<10 ppm) and matching MSMS fragments to the NIST spectral library. The symbol **indicates that the compound name was confirmed by accurate mass (<10 ppm), retention time, and MSMS match to purchased standards. These are MSI 1. All other compound names are MSI 2 and are based on accurate mass (<10 ppm) and isotopic abundance and distribution.

### Data processing

#### Metabolomics

Processing of metabolomics data was performed using the R package MSPrep^76^. Only compounds identified in at least 2 of 3 sample vial injection triplicates were summarized to obtain one measurement per compound per subject, all others were filtered out. A maximum coefficient of variation of 0.5 was set as a threshold for utilizing the mean of the triplicates or the median of the triplicates. This threshold was selected to account for when the extraction software may have missed a peak, thus resulting in one replicate having low to no signal and therefore causing a larger CV across replicates. The data was then filtered for compounds that were found in at least 80 percent of subjects. Missing data was then imputed using Bayesian Principal Component Analysis (BPCA), which uses linear combinations of principal axis vectors to estimate the missing value and is not sensitive to the level of missing data^77^. The data was then normalized using the Cross-Contribution Compensating Multiple Standard Normalization (CRMN) method to remove unwanted batch variation^78^. Following filtering, imputation, and normalization the numbers of metabolites decreased from 1,813 to 662 and 6,183 to 2,337 compounds in the aqueous and lipid fractions respectively, for a total of 2,999 compounds. Many of the compounds that were removed from further analysis were attributed to instrument noise, software extraction artefacts, and single hits from individual subjects.

#### Functional Genomics

Transcript expression was measured using Affymetrix Human Genome U133 plus 2.0 Gene Array (GEO accession number GSE42057; Affymetrix, Santa Clara, CA). Quality control, batch correction, normalization, filtering, and log transformation was performed as previously described^69^. Following filtering, transcripts were reduced from 54,675 to 12,531. Subsequent statistical analysis on the reduced transcript list is described below.

### Statistical analysis

To assess the significance of transcript and metabolite levels in COPD phenotypes, a series of regression models were fit for each outcome and the form of the model depended on the distribution of the outcome. For FEV_1_% predicted, a linear regression was used. For FEV_1_/FVC and % emphysema beta regressions were used. Number of exacerbations was modeled as a negative binomial; since many subjects had no exacerbations, a zero-inflation portion was added. Severity of exacerbations used logistic regression. Since BDR was constrained between 0 (no response) and 1 (positive response), logistic regression was used for BDR. For all models, covariates were included. Covariates for FEV1% predicted were age, BMI, gender, smoking status, parents have COPD, and ATS Pack-years. Covariates for FEV_1_/FVC were age, gender, asthma, and smoking status. Covariates for % emphysema were age, gender, BMI, FEV_1_% predicted, and smoking status. Covariates for exacerbation frequency and exacerbation severity were FEV_1_% predicted, gastro esophageal reflux, St. George’s Respiratory Questionnaire (SGRQ) score, and gender. Covariates for BDR were age, gender, smoking status and asthma. Significant differences within the cohort, such as age and smoking, were controlled for in subsequent analyses.

Then, to assess the significance of various metabolites and transcripts, the log-transformed metabolite or transcript levels were added to the models as the independent variable, and the significance of the effect of these metabolite or transcript levels were reported. For the transcripts, p ≤ 0.015 was considered significant with FDR ≤ 0.1 adjustment. For the metabolites, p ≤ 0.05 was considered significant with FDR ≤ 0.15 adjustment. All analyses were performed in R^79^.

### Pathway enrichment analysis

The significant transcript probes were converted to official gene symbols using DAVID Bioinformatics Database version 6.8^80^ ID conversion tool. The significant compounds were converted to KEGG IDs using Chemical Translation Service^81^. The lists of statistically significant transcripts and metabolites within each COPD outcome were searched using IMPaLA^82^ to obtain pathways relevant to each outcome. In IMPaLA, the gene transcripts and associated values were uploaded using ‘gene symbol’, the metabolites and associated values were uploaded in the ‘KEGG’ identifier, and pathway over-representation analysis was performed. A meta-analysis using Fisher’s method provided a combined p-value and a corrected p-value for the pathways. The results were filtered as follows: KEGG pathways, p ≤ 0.05, FDR ≤ 0.2 and ≥3 pathway hits. A more lenient FDR ≤ 0.2 cut-off was used since the individual samples within each group for each outcome or phenotype were small. Figure 1 highlights the experimental procedure used to generate results from the two datasets.

### Visualization

Venn diagrams were created to show the overlap of statistically significant transcript probes, compounds, and pathways for the COPD outcomes using the publicly available jvenn program^83^.

Cellular function relationships for each outcome were visualized using the ‘Omics Dashboard’ of HumanCyc^23^ (https://humancyc.org/dashboard/dashboard-intro.shtml) using ‘Analysis’. The gene symbols for the significant transcript probes and their coefficients were uploaded in tab-delimited format for expression analysis. The KEGG IDs for the significant compounds and their coefficients were uploaded in tab-delimited format for metabolomics analysis. The interactions between the top gene transcripts and top metabolites were explored using ConsensusPathDB (http://consensuspathdb.org/)^84^ and selecting ‘over-representation analysis’. The selected identifiers were ‘gene symbol’ and ‘ChEBI’ to determine gene interactions and pathways interactions for the gene transcripts and metabolites, respectively. For the transcriptomics data, protein, genetic, biochemical, and gene regulatory interactions were considered; for protein interactions, only binary protein interactions with high and medium confidence were allowed, while complex interactions were ignored. Intermediate nodes were allowed for all interactions. For the metabolomics data, only KEGG pathways with a minimum overlap of 2 and a p-value cut-off of 0.05 was allowed. Finally, outcome and pathway relationships were visualized using KEGG^85^ to plot pathways for each outcome.

## Electronic supplementary material

Supplementary Documents

## Data Availability

The mass spectrometry data from this publication has been deposited to the Metabolomics Workbench database http://www.metabolomicsworkbench.org/ and assigned the identifier PR000438. The microarray data from this publication is available in the Gene Expression Omnibus (GEO) database https://www.ncbi.nlm.nih.gov/geo and assigned the identifier GSE42057^69^.
