# Supplementary material for: Metabolomics and transcriptomics pathway approach reveals outcome-specific perturbations in COPD
Source: Sci Rep. 2018 Nov 20;8:17132. doi: 10.1038/s41598-018-35372-w (PMC6244246; doi:10.1038/s41598-018-35372-w)
Supplement: Supplementary file 1 — Supplementary Documents [file 41598_2018_35372_MOESM1_ESM.docx]

**Metabolomics and transcriptomics pathway approach reveals outcome-specific perturbations in COPD**

**Charmion I. Cruickshank-Quinn, PhD^1^, Sean Jacobson, MS^3^, Grant Hughes, MS^5^, Roger L. Powell, MS^1^, Irina Petrache, MD^3,^**^4^**, Katerina Kechris, PhD^2^, Russell Bowler, MD PhD^3,^**^4^***, Nichole Reisdorph, PhD^1^***

^1^ Department of Pharmaceutical Sciences, University of Colorado Anschutz Medical Campus, Aurora, CO 80045

^2^ Department of Biostatistics and Informatics, University of Colorado Anschutz Medical Campus, Aurora, CO 80045

^3^ Department of Medicine, National Jewish Health, Denver, CO 80206

^4^ Department of Medicine, University of Colorado Anschutz Medical Campus, Aurora, CO 80045

^5^ Flathead Valley Community College, Kalispell, MT 59901

**Supplemental Documents**

| **Name** | **Title** | **Page #** |
| --- | --- | --- |
| Supplementary Document S1 | Interactions of the top gene transcripts and metabolites | 2 - 6 |
| Supplementary Document S2 | Significant compounds in unique pathways | 7 - 8 |
| Supplementary Document S3 | Exacerbations outcome differences. (A) Degradation pathways in exacerbation frequency. (B) Energy and degradation pathways in exacerbation severity. | 9 |
| Supplementary Document S4 | Glycerolipid metabolism in FEV_1_/FVC. | 10 |
| Supplementary Document S5 | Compound identifications based on MSMS of purchased standards or spectral libraries. | 11 – 14 |
| Supplementary Document S6 | Putatively identified compounds based on accurate mass, isotope abundance and isotope distribution. | 14 - 26 |

**Supplementary Document S1: Interactions of the top gene transcripts and metabolites.**

**Part A. Gene interactions**


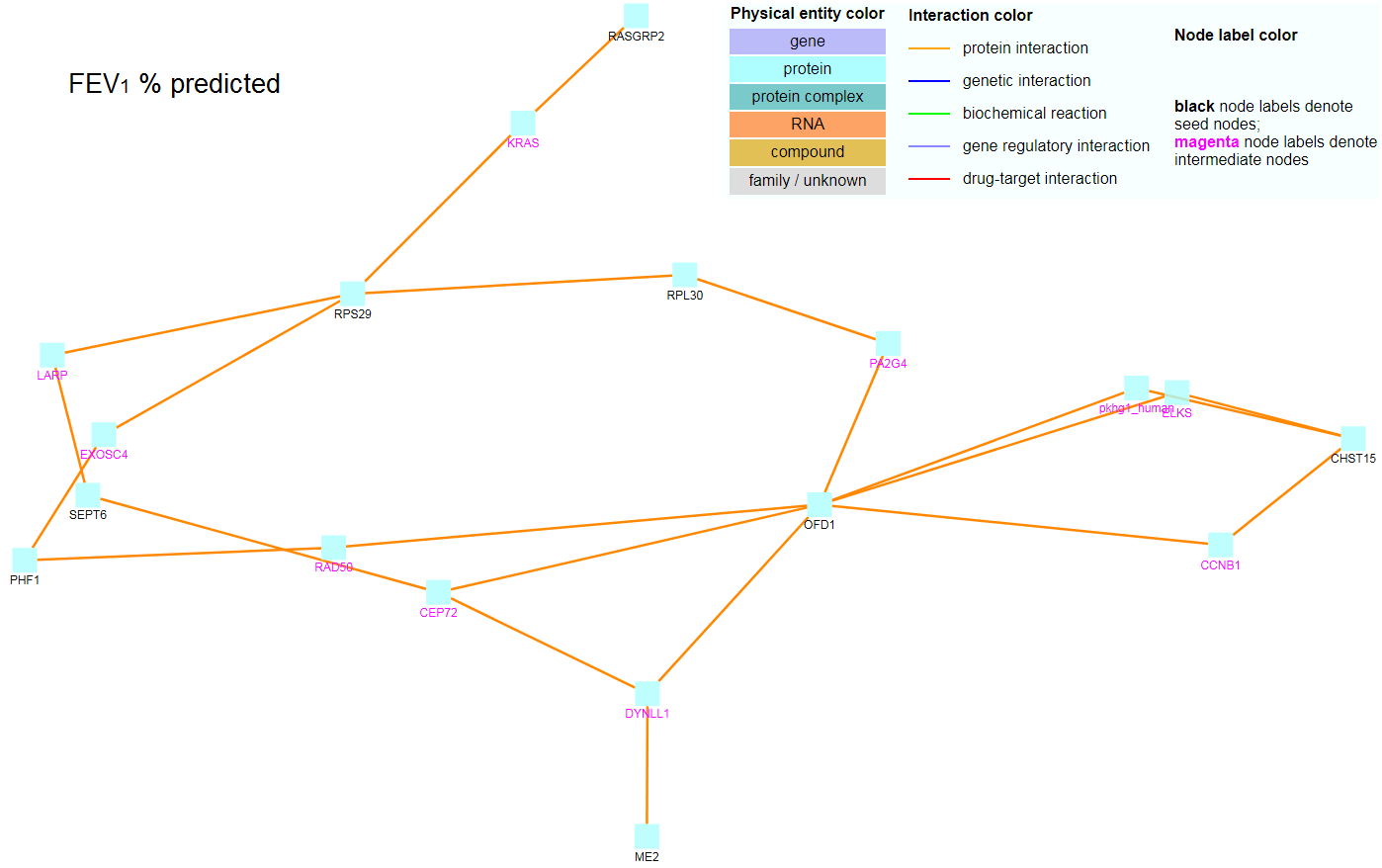


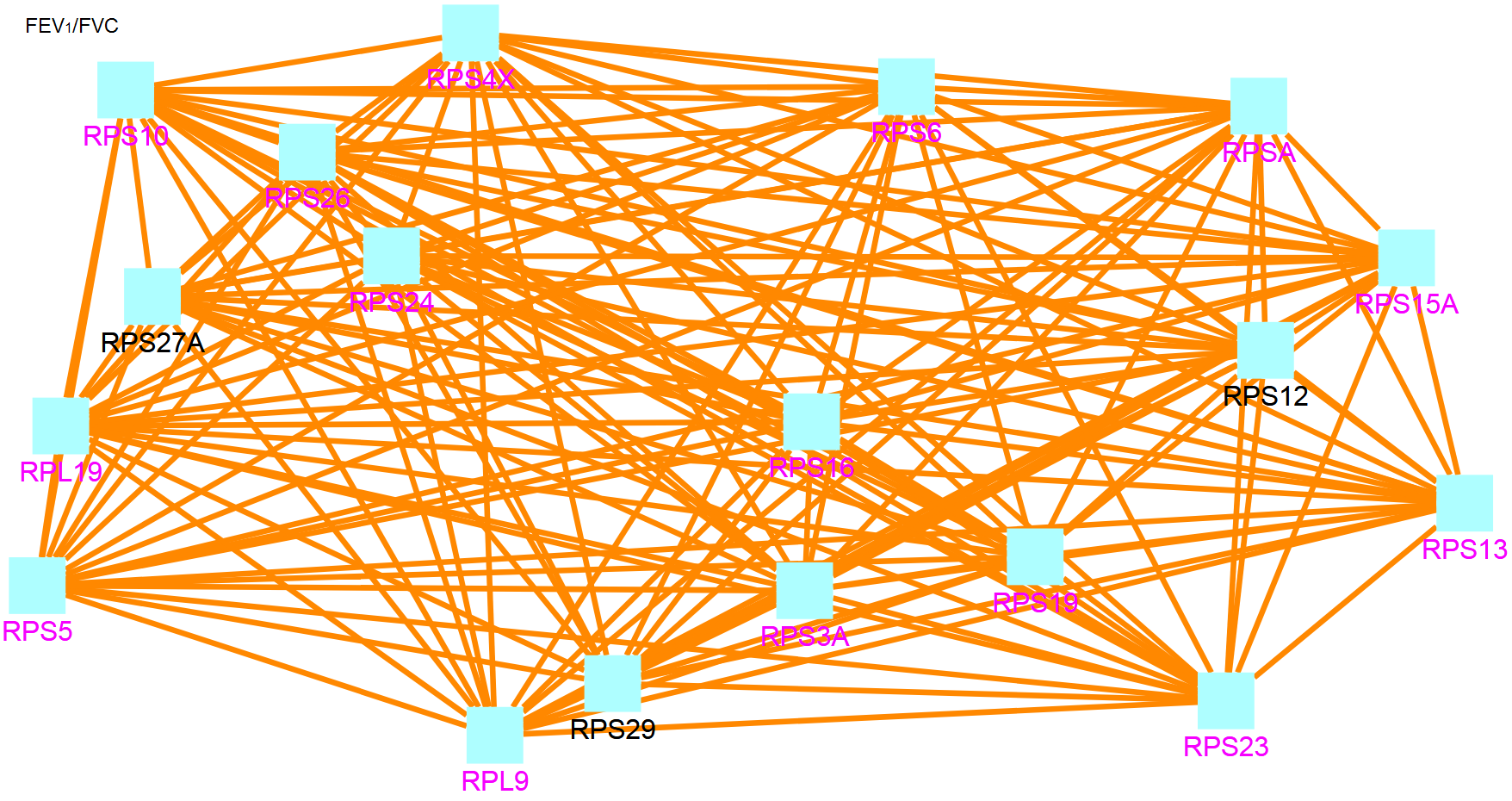


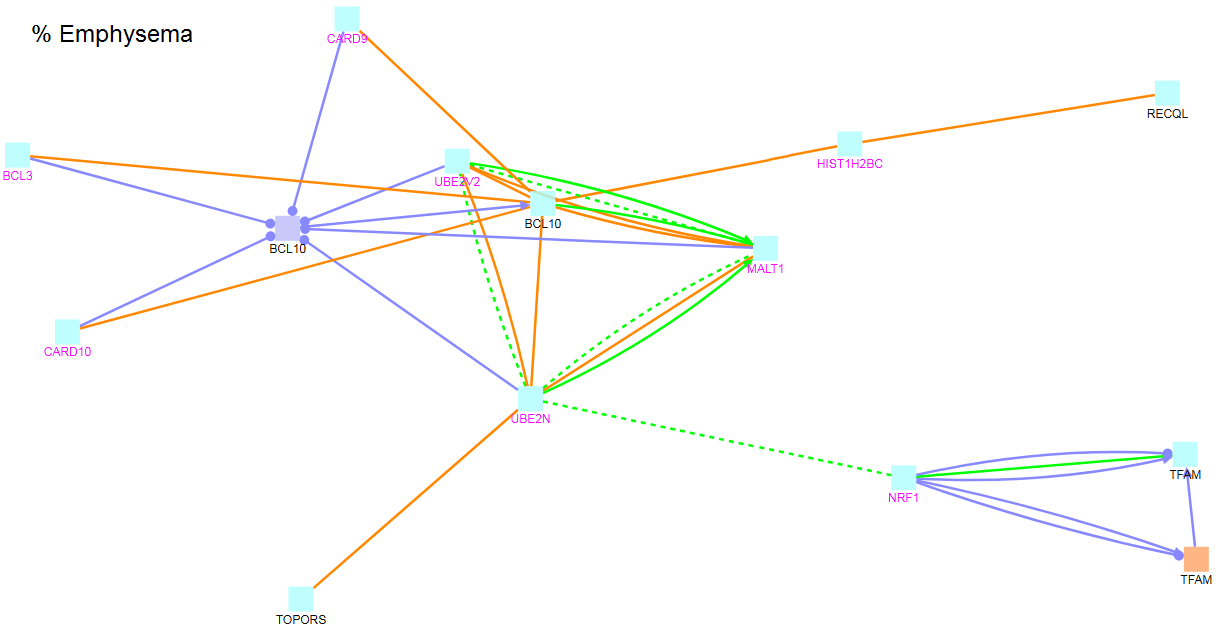


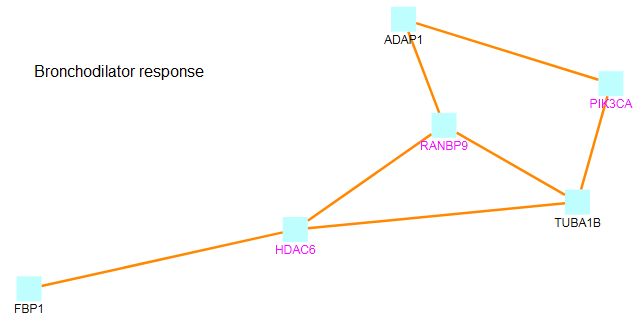


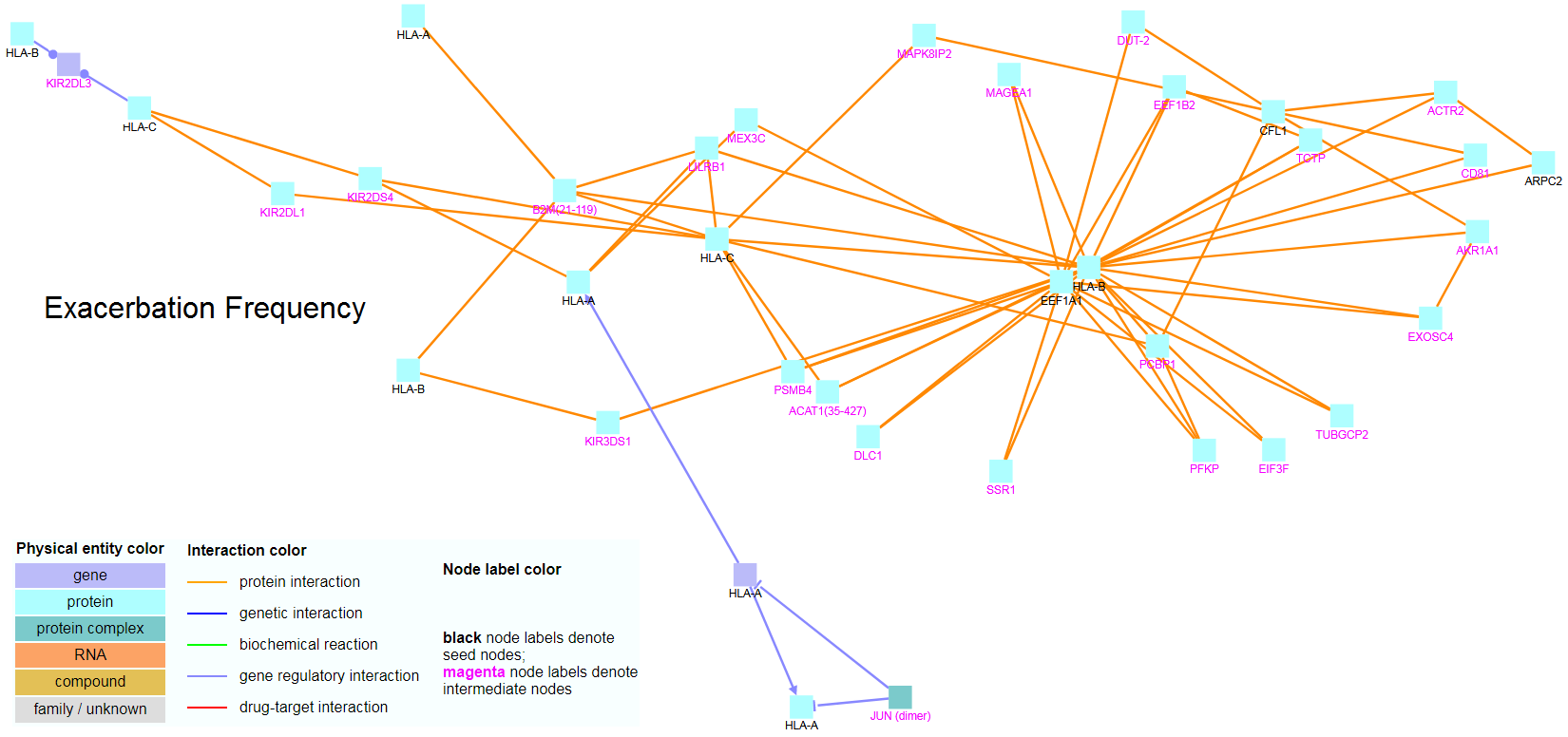


**Part B. Metabolite interactions**


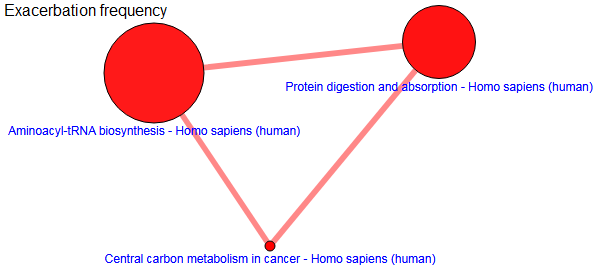


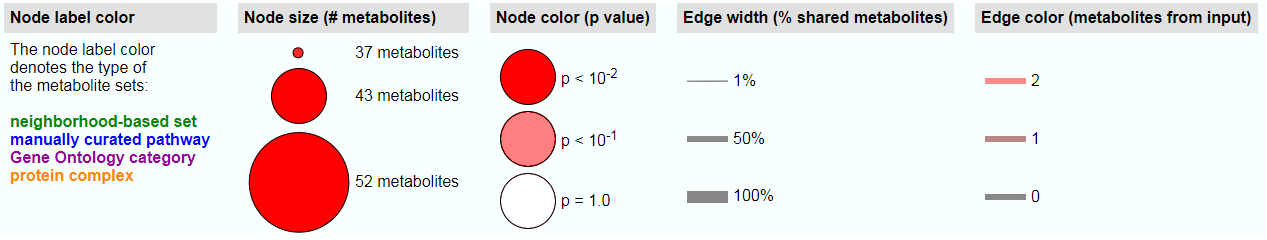


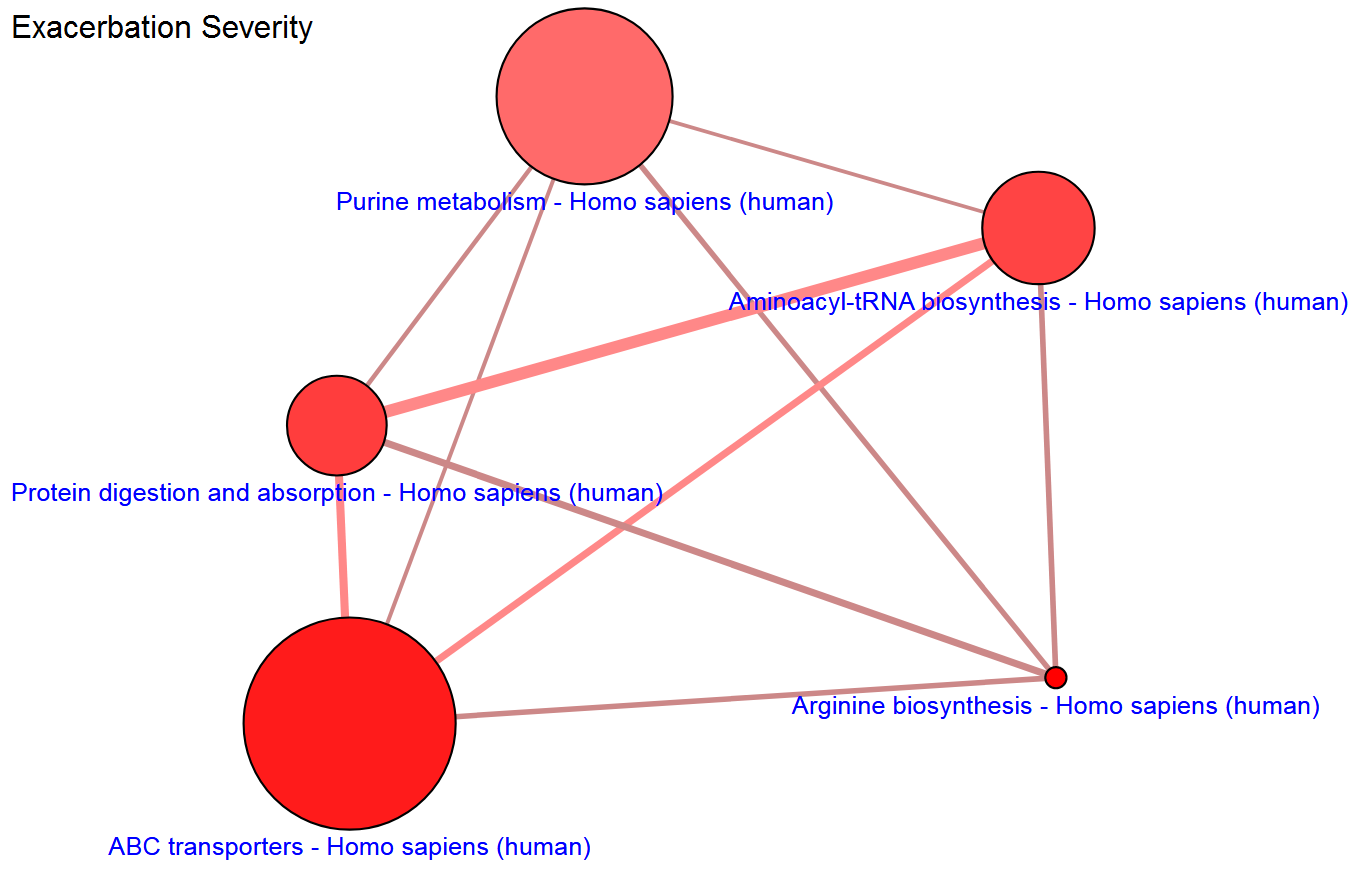


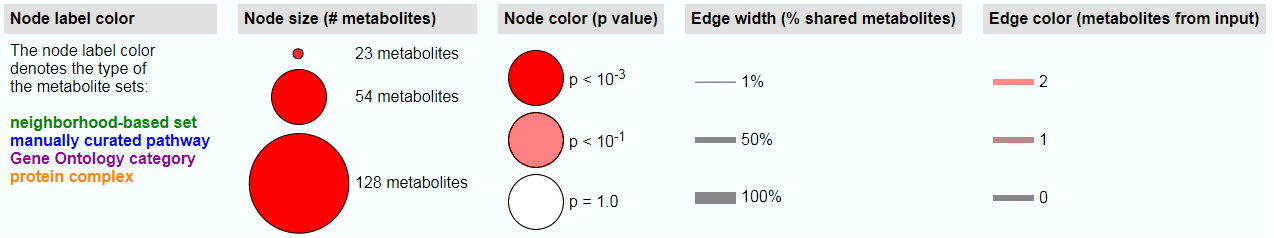


**Supplementary Document S2. Significant compounds in unique pathways.**

| **Outcome** | **Unique Significant Pathways** | **# Hits** | **Significant species** | **p-value** | **FDR** |
| --- | --- | --- | --- | --- | --- |
| **FEV_1_/FVC** | Fat digestion and absorption | 10 | MG(20:2), DG(35:6), DG(34:1), DG(36:2), DG(36:4), DG(36:5), DG(46:0), DG(O-34:1), TG(o-54:4), TG(36:0), TG(42:0), TG(54:7), CE(20:4), CE(18:1), PA(28:0), PA(44:3), PA(22:2), PA(44:2), Additional phospholipids: PC(40:7), PC(36:2), PC(36:4), PC(36:5), PC(O-38:4), PE(34:2), PE(39:3), PE(P-38:2), PE(O-38:5), PE(P-36:4), PG(30:0), PG(29:2), PI(36:2), PS(39:7), PS(40:0), PS(39:2), PS(42:2), Bile acid: (23R)-3α,7α,12α,23-Tetrahydroxy-5β-cholan-24-oic Acid, Fatty acid: Docosahexaenoic acid; *ABCA1, SCARB1* | 7.49E-05 | 0.004 |
|  | Hematopoietic cell lineage | 17 | *CD14, CD1D, CD2, CD33, CD3D, CD3E, CD44, CD5, CD55, CD59, CD7, CSF3R, FLT3LG, HLA-DMB, HLA-DPB1, ITGA6, IL7R* | 5.29E-04 | 0.037 |
|  | Glycerolipid metabolism | 11 | PA(28:0), PA(44:3), PA(22:2), PA(44:2), MG(20:2), TG(o-54:4), TG(36:0), TG(42:0), TG(54:7), DG(36:2), DG(36:4), DG(36:5), DG(35:6), DG(46:0); *ALDH2, DGKA, DGKD, DGKE, GLA, AGPAT9, GPAT3* | 0.002 | 0.051 |
| **FEV_1_ % Predicted** | T cell receptor signaling pathway | 22 | *AKT2, CD247, CD28, CD3D, CD3E, ITK, LCK, NCK2, RASGRP1, GRB2, LAT, MAPK1, MAPK14, NFATC2, PAK1, PAK2, PIK3R1, PRKCQ, PPP3CA, PPP3CC, RHOA, ZAP70* | 1.12E-04 | 0.0042 |
|  | Fc gamma R-mediated phagocytosis | 14 | PA(44:2), PA(O-41:0); *AKT2, ASAP1, CRK, HCK, ARPC5L, BIN1, LAT, MAPK1, PAK1, PIK3R1, PLCG2, RAC1, SYK* | 0.002 | 0.039 |
|  | Peroxisome | 14 | *ACSL1, ACSL4, ACSL6, AGPS, CAT, FAR1, FAR2, GNPAT, HSD17B4, IDH1, PEX16, PEX26, PXMP4, SOD1* | 0.004 | 0.072 |
|  | Hippo signaling pathway | 18 | *LLGL1, LOC400927-CSNK1E, MOB1A, MOB1B, SMAD4, CTNNA1, CCND3, DVL1, DVL2, FZD1, LATS2, LEF1, PPP1CB, PPP2CB, STK3, TCF7, YWHAE, YWHAG* | 0.004 | 0.075 |
|  | mTOR signaling pathway | 10 | *AKT2, RHEB, RRAGD, E1F4EBP1, E1F4E2, MAPK1, PTEN, PIK3R1, RPS6, ULK3* | 0.004 | 0.086 |
|  | NF-kappa B signaling pathway | 17 | *BCL2, BTK, LCK, TAB2, TRAF5, TNFRSF1A, BIRC3, LAT, LY96, LTBR, LTB, MYD88, PLCG2, PRKCQ, SYK, TNFSF13B, ZAP70* | 0.005 | 0.092 |
|  | Endocytosis | 39 | *ARF3, ARAP1, ARAP3, ASAP1, ACAP1, EHD1, GRK3, GIT2, KIAA0196, KIAA1033, RAB10, RAB11FIP1, RAB5A, SH3GLB1, VPS26A, VPS35, VPS37C, ARPC5L, ARRB1, BIN1, CAPZA2, CHMP5, CLTA, IL2RB, IL2RG, ITCH, KIF5B, HLA-E, RBSN, RHOA, STAM2, SNX1, SNX2, SNX3, SNX5, SNX6, TSG101, USP8, VTA1* | 0.005 | 0.092 |
|  | Neurotrophin signaling pathway | 15 | Ceramide (d18:1/24:1); *AKT2, BCL2, CRK, GRB2, IRAK2, MAPK1, MPAK14, MAP3K5, PIK3R1, PLCG2, PSEN1, RHOA, RAC1, YWHAE* | 0.007 | 0.108 |
|  | Jak-STAT signaling pathway | 19 | *AKT2, JAK1, JAK2, JAK3, CSF2RB, CCND3, GRB2, IFNGR1, IFNGR2, IL10RB, IL12RB1, IL13RA1, IL15, IL2RB, IL2RG, IL4R, PIK3R1, STAT5B, STAM2* | 0.008 | 0.120 |
| **Exacerbation Frequency** | Antigen processing and presentation | 4 | *HLA-A, HLA-B, HLA-C, B2M* | 0.002 | 0.113 |
| **Exacerbation Severity** | Arginine and proline metabolism | 4 | Proline, Creatinine, Ornithine, Aspartic acid beta-semialdehyde | 2.38E-04 | 0.005 |
|  | ABC transporters | 6 | Betaine, Orthophosphate, Mannitol, Proline, Ornithine, Glutamine | 3.97E-04 | 0.006 |
|  | Glycine, serine and threonine metabolism | 4 | Tryptophan, PS(22:2), Betaine, Aspartic acid beta-semialdehyde | 0.002 | 0.016 |
|  | Retrograde endocannabinoid signaling | 3 | DG(43:4), PC(38:5), PC(36:3), PC(20:5), PE(41:4) | 0.001 | 0.043 |
|  | Insulin resistance | 3 | Ceramide (d18:1/24:1), DG(43:4), Acetylcarnitine | 0.002 | 0.057 |
|  | Arginine biosynthesis | 3 | Glutamine, Citrulline, Ornithine | 0.003 | 0.067 |
|  | Purine Metabolism | 4 | Hypoxanthine, Glutamine, Urate, 5-Hydroxyisourate | 0.018 | 0.083 |
|  | Phenylalanine, tyrosine and tryptophan biosynthesis | 3 | Tyrosine, Tryptophan, Aspartic acid beta-semialdehyde | 0.008 | 0.143 |
| **% Emphysema** | Oxidative phosphorylation | 11 | *ATP5F1, ATP5J, ATP6V0E1, ATP6V1A, ATP6V1D, NDUFAB1, NDUFB1, NDUFB6, PPA, SDHC, SDHD* | 1.83E-04 | 0.113 |
|  | mRNA surveillance pathway | 8 | *CSTF2, NXF1, NXT2, NUDT21, PCF11, PELO, PPP2R5E* | 9.24E-04 | 0.199 |

**Supplementary Document S3: Exacerbations outcome differences. (A)** Degradation pathways in exacerbation frequency. **(B)** Energy and degradation pathways in exacerbation severity.


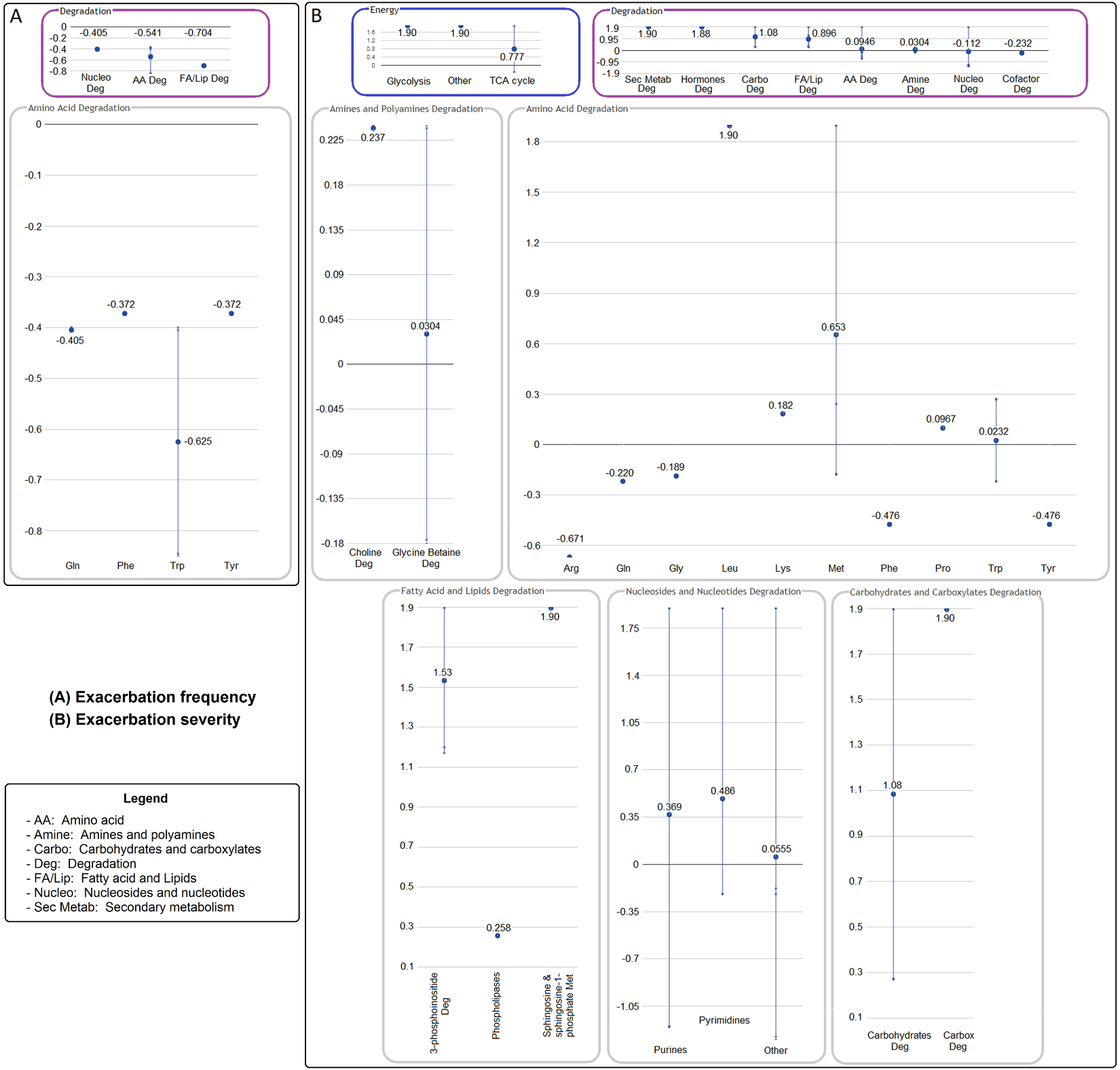


**Supplementary Document S4: Glycerolipid metabolism in FEV_1_/FVC.** Red indicates associated with an increase with worsening disease. Blue indicates associated with a decrease with worsening disease. Circles are compounds and rectangles are genes. Image modified from KEGG^1-3^.


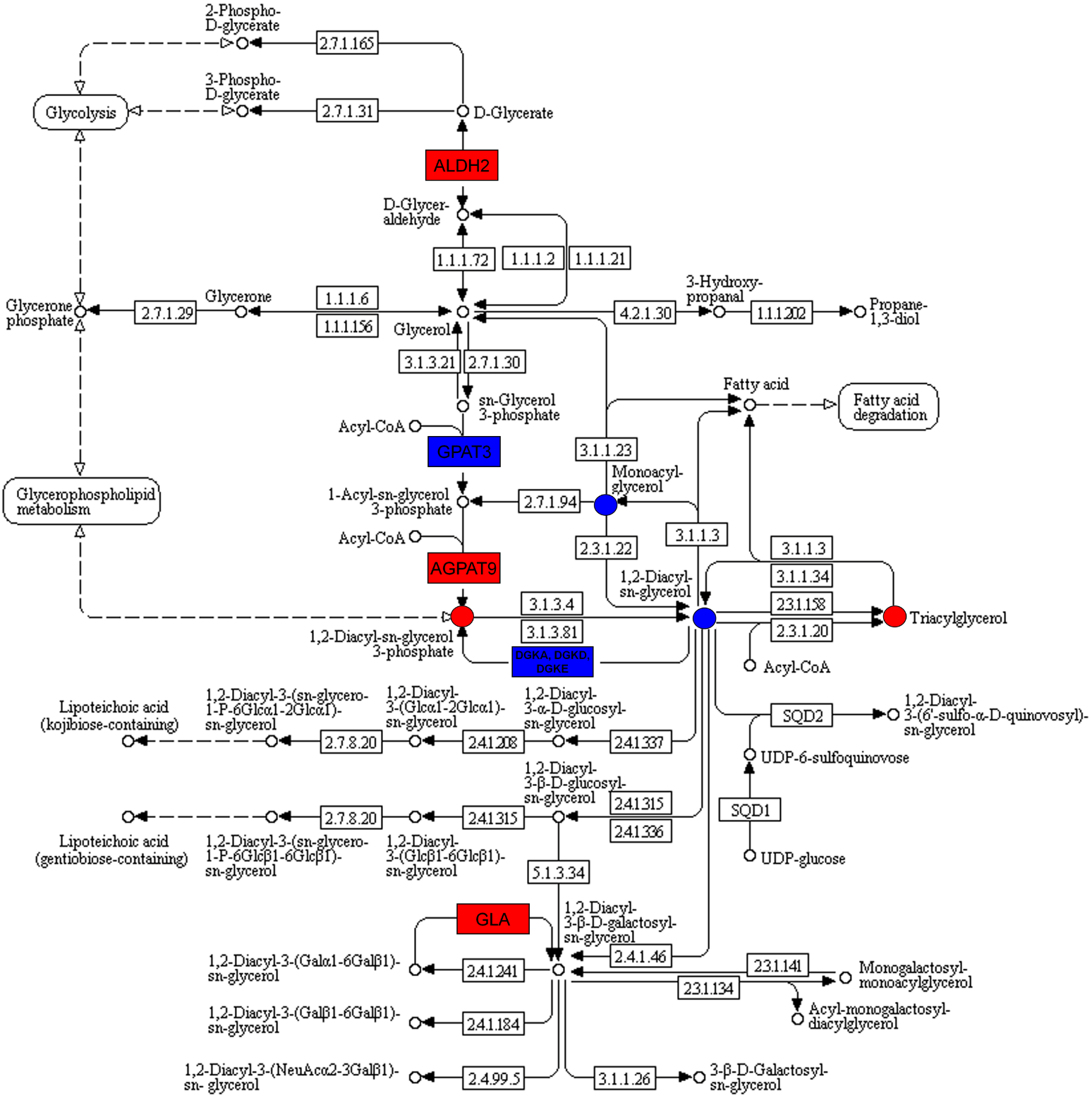


**References**

1 Kanehisa, M., Furumichi, M., Tanabe, M., Sato, Y. & Morishima, K. KEGG: new perspectives on genomes, pathways, diseases and drugs. *Nucleic Acids Res.* **45**, D353-d361, doi:10.1093/nar/gkw1092 (2017).

2 Kanehisa, M. & Goto, S. KEGG: Kyoto Encyclopedia of Genes and Genomes. *Nucleic Acids Res.* **28**, 27-30 (2000).

3 Kanehisa, M., Sato, Y., Kawashima, M., Furumichi, M. & Tanabe, M. KEGG as a reference resource for gene and protein annotation. *Nucleic Acids Res.* **44**, D457-462, doi:10.1093/nar/gkv1070 (2016).

**Supplementary Document S5: Compound identifications based on MSMS of purchased standards or spectral libraries.** In the table below, ** indicates that the compound was matched to mass, retention and MSMS from purchased standards, while * indicates a positive match to MSMS fragments from the NIST14 and/or NIST17 spectral library. The designation *- refers to compounds for which standards were not available or were not present in the NIST spectral library and were therefore matched to fragments in MetFrag.

| **Compound** | **Formula** | **RT**  **(mins)** | **CAS ID** | **HMP ID** | **LMP ID** | **Frac-tion** |
| --- | --- | --- | --- | --- | --- | --- |
| (23S)-26,26,26,27,27,27-hexafluoro-1alpha,23,25-trihydroxyvitamin D3*- | C27 H38 F6 O4 | 3.841 |  |  | LMST03020090 | HILIC |
| (S)-3-Hydroxy-N-methylcoclaurine*- | C18 H21 N O4 | 3.777 |  | HMDB06921 |  | HILIC |
| 1,6-Anhydro-b-D-glucopyranose* | C6 H10 O5 | 4.927 | 498-07-7 | HMDB00640 |  | HILIC |
| 14(15)-EET** | C20 H32 O3 | 5.388 |  | HMDB04264 | LMFA03080005 | C18 |
| 1-Methyl-4-pyridone-5-carboxamide* | C7 H8 N2 O2 | 0.500 | 769-49-3 | HMDB04194 |  | HILIC |
| 1-METHYLADENOSINE** | C11 H15 N5 O4 | 0.642 | 15763-06-1 | HMDB03331 |  | HILIC |
| 1-Methylnicotinamide* | C7 H8 N2 O | 5.628 | 114-33-0 | HMDB03152 |  | HILIC |
| 2,4-DIHYDROXYPTERIDINE** | C6 H4 N4 O2 | 1.620 | 487-21-8 |  |  | C18 |
| 2-DEOXY-D-GLUCOSE** | C6 H12 O5 | 2.328 | 154-17-6 |  |  | HILIC |
| 2-Keto-6-acetamidocaproate*- | C8 H13 N O4 | 7.168 |  | HMDB12150 |  | HILIC |
| 3-METHYLADENINE** | C6 H7 N5 | 0.638 | 5142-23-4 | HMDB11600 |  | HILIC |
| 3-Methylhistidine* | C7 H11 N3 O2 | 7.156 |  | HMDB00479 |  | HILIC |
| 4-(2-Aminoethyl)benzenesulfonyl fluoride* | C8 H10 F N O2 S | 1.732 |  |  |  | C18 |
| 4-GUANIDINOBUTANOATE** | C5 H11 N3 O2 | 4.810 | 463-00-3 | HMDB03464 |  | HILIC |
| 4-PYRIDOXATE** | C8 H9 N O4 | 0.540 | 82-82-6 | HMDB00017 |  | HILIC |
| 5-beta-Androsterone* | C19 H30 O2 | 2.563 | 53-42-9 | HMDB00490 | LMST02020059 | C18 |
| 5-OXO-D-PROLINE** | C5 H7 N O3 | 1.865 | 4042-36-8 | HMDB00805 |  | HILIC |
| 6-Aminocaproic acid* | C6 H13 N O2 | 0.927 |  |  |  | HILIC |
| Acetylcarnitine* | C9 H17 N O4 | 1.597 |  | HMDB00201 |  | HILIC |
| Acetylcholine* | C7 H16 N O2 | 8.595 | 51-84-3 | HMDB00895 |  | HILIC |
| Adenosine* | C10 H13 N5 O4 | 0.199 | 58-61-7 | HMDB00050 |  | HILIC |
| ALPHA-D-GLUCOSE** | C6 H12 O6 | 4.707 | 492-62-6 | HMDB03345 |  | HILIC |
| AMPA** | C7 H10 N2 O4 | 7.002 | 77521-29-0 |  |  | HILIC |
| Arachidonoylthio-PC* | C44 H82 N O6 P S | 2.971 | 146797-82-2 |  |  | HILIC |
| Aspartyl-Threonine*- | C8 H14 N2 O6 | 6.473 |  |  |  | HILIC |
| Benzhydrol* | C13 H12 O | 0.696 |  |  |  | HILIC |
| Bestatin* | C16 H24 N2 O4 | 6.919 | 58970-76-6 |  |  | HILIC |
| Bestatin* | C16 H24 N2 O4 | 1.287 | 65391-42-6 |  |  | HILIC |
| beta-D-Glucose* | C6 H12 O6 | 0.926 |  |  |  | HILIC |
| Betaine* | C5 H11 N O2 | 4.017 | 107-43-7 | HMDB00043 |  | HILIC |
| BILIRUBIN** | C33 H36 N4 O6 | 6.048 | 635-65-4 | HMDB00054 |  | C18 |
| BILIVERDIN** | C33 H34 N4 O6 | 2.256 | 55482-27-4 |  |  | C18 |
| BIS(2-ETHYLHEXYL)PHTHALATE** | C24 H38 O4 | 0.927 | 117-81-7 |  |  | HILIC |
| Butenoyl PAF* | C28 H56 N O7 P | 1.014 |  |  |  | C18 |
| Ceramide (d18:1/16:0)* | C34 H67 N O3 | 1.257 |  | HMDB04949 | LMSP02010004 | C18 |
| Ceramide (d18:1/18:0)* | C36 H71 N O3 | 7.187 | 104404-17-3 | HMDB04950 |  | C18 |
| Ceramide (d18:1/22:0)* | C40 H79 N O3 | 6.286 | 104404-17-3 | HMDB04952 |  | C18 |
| CHENODEOXYCHOLATE** | C24 H40 O4 | 5.086 | 474-25-9 | HMDB00518 |  | C18 |
| CHOLATE** | C24 H40 O5 | 9.228 | 81-25-4 | HMDB00619 |  | HILIC |
| Cholesterol* | C27 H46 O | 5.380 | 57-88-5 | HMDB00067 |  | C18 |
| CHOLESTERYL ACETATE** | C29 H48 O2 | 1.039 | 604-35-3 | HMDB03822 |  | C18 |
| Choline* | C5 H13 N O | 5.704 | 62-49-7 | HMDB00097 |  | HILIC |
| cis-7-Hexadecenoic acid methyl ester* | C17 H32 O2 | 2.199 | 56875-67-3 |  |  | C18 |
| CITRULLINE** | C6 H13 N3 O3 | 5.308 | 372-75-8 | HMDB00904 |  | HILIC |
| COENZYME Q10** | C59 H90 O4 | 5.138 | 303-98-0 |  |  | C18 |
| CORTISOL** | C21 H30 O5 | 2.035 | 50-23-7 | HMDB00063 |  | C18 |
| CORTISONE** | C21 H28 O5 | 2.037 | 53-06-5 | HMDB02802 |  | C18 |
| Cotinine* | C10 H12 N2 O | 0.462 | 50-67-9 | HMDB00259 |  | HILIC |
| Creatine* | C4 H9 N3 O2 | 4.590 | 57-00-1 | HMDB00064 |  | HILIC |
| CREATININE** | C4 H7 N3 O | 3.814 | 60-27-5 | HMDB00562 |  | HILIC |
| DEOXYCHOLATE** | C24 H40 O4 | 2.919 | 302-95-4 |  |  | HILIC |
| DG(34:1)* | C37 H70 O5 | 7.186 |  | HMDB07102 |  | C18 |
| DG(34:2)* | C37 H68 O5 | 2.061 |  | HMDB07103 |  | C18 |
| DG(36:3)* | C39 H70 O5 | 5.855 |  | HMDB07219 |  | C18 |
| Diethylhexyl adipate (DEHA)* | C22 H42 O4 | 1.435 | 103-23-1 | HMDB40270 |  | C18 |
| DL-2-Aminocaprylic acid* | C8 H17 N O2 | 1.338 |  | HMDB00991 |  | HILIC |
| Docosahexaenoic acid** | C22 H32 O2 | 4.909 | 6217-54-5 | HMDB02183 | LMFA01030185 | C18 |
| D-PANTOTHENIC ACID** | C9 H17 N O5 | 4.019 | 137-08-6 |  |  | HILIC |
| Edetic Acid* | C10 H16 N2 O8 | 3.789 | 62-33-9 | HMDB15109 |  | HILIC |
| Eicosapentaenoyl PAF C-16* | C44 H80 N O7 P | 8.206 |  |  |  | C18 |
| FUCOSE** | C6 H12 O5 | 0.641 | 2438-80-4 | HMDB00174 |  | HILIC |
| gamma-Glutamyl-serine*- | C8 H14 N2 O6 | 6.449 | 5875-35-4 | HMDB29158 |  | HILIC |
| GLUCOSAMINATE** | C6 H13 N O6 | 1.620 | 3646-68-2 |  |  | C18 |
| GLUTATHIONE** | C10 H17 N3 O6 S | 2.037 | 70-18-8 | HMDB00125 |  | C18 |
| Glycerophosphocholine* | C8 H20 N O6 P | 7.753 | 28319-77-9 | HMDB00086 |  | HILIC |
| GLYCERYL TRIMYRISTATE** | C45 H86 O6 | 6.012 | 555-45-3 |  |  | C18 |
| GLYCOCHOLATE** | C26 H43 N O6 | 2.147 | 475-31-0 | HMDB00138 |  | C18 |
| Hexadecylamine* | C16 H35 N | 5.827 | 143-27-1 |  |  | C18 |
| HIPPURATE** | C9 H9 N O3 | 3.392 | 495-69-2 | HMDB00714 |  | HILIC |
| HYPOXANTHINE** | C5 H4 N4 O | 2.089 | 68-94-0 | HMDB00157 |  | HILIC |
| Indoleacrylic acid* | C11 H9 N O2 | 1.676 | 1204-06-4 | HMDB000734 |  | HILIC |
| Isodesmosine*- | C24 H40 N5 O8 | 5.786 | 991-01-5 | HMDB000739 |  | HILIC |
| L-ANSERINE** | C10 H16 N4 O3 | 0.690 | 10030-52-1 |  |  | HILIC |
| L-ARGININE** | C6 H14 N4 O2 | 3.241 | 1119-34-2 | HMDB00517 |  | HILIC |
| LAUROYLCARNITINE** | C19 H37 N O4 | 4.662 | 25518-54-1 |  |  | C18 |
| L-Carnitine* | C7 H15 N O3 | 4.702 | 6645-46-1 |  |  | HILIC |
| L-CARNITINE** | C7 H15 N O3 | 0.344 | 6645-46-1 |  |  | C18 |
| Leucine* | C6 H13 N O2 | 9.037 |  | HMDB00687 |  | HILIC |
| Leucine** | C6 H13 N O2 | 0.759 |  | HMDB00687 |  | C18 |
| Leupeptin* | C20 H38 N6 O4 | 3.353 | 103476-89-7 |  |  | HILIC |
| L-GLUTAMIC ACID** | C5 H9 N O4 | 0.318 | 56-86-0 | HMDB00148 |  | HILIC |
| L-GLUTAMINE** | C5 H10 N2 O3 | 6.905 | 56-85-9 | HMDB00641 |  | HILIC |
| L-NORVALINE** | C5 H11 N O2 | 2.094 | 6600-40-4 |  |  | HILIC |
| L-Palmitoylcarnitine* | C23 H45 N O4 | 5.599 | 2364-67-2 | HMDB00222 | LMFA07070079 | C18 |
| L-PHENYLALANINE** | C9 H11 N O2 | 0.858 | 63-91-2 | HMDB00159 |  | HILIC |
| Lysine* | C6 H14 N2 O2 | 11.134 | 56-87-1 | HMDB00182 |  | HILIC |
| LysoPC(14:0)* | C22 H46 N O7 P | 4.911 |  | HMDB10379 |  | C18 |
| LysoPC(16:0)* | C24 H50 N O7 P | 5.722 |  |  | LMGP01050018 | C18 |
| LysoPC(17:0)* | C25 H52 N O7 P | 6.561 |  | HMDB12108 | LMGP01050024 | C18 |
| LysoPC(18:0)* | C26 H54 N O7 P | 5.524 | 5655-17-4 | HMDB10384 | LMGP01050026 | C18 |
| LysoPC(18:1)* | C26 H52 N O7 P | 6.422 | 3542-29-8 | HMDB02815 | LMGP01050032 | C18 |
| LysoPC(18:2)* | C26 H50 N O7 P | 3.859 |  | HMDB10386 |  | HILIC |
| LysoPC(20:0)* | C28 H58 N O7 P | 0.452 |  | HMDB10390 |  | C18 |
| LysoPC(20:4)* | C28 H50 N O7 P | 3.826 |  | HMDB10396 |  | HILIC |
| LysoPC(O-18:0)* | C26 H56 N O6 P | 0.451 | 72490-82-5 | HMDB11149 | LMGP01060014 | C18 |
| LysoPC(P-18:0)* | C26 H54 N O6 P | 8.999 | 103597-60-0 | HMDB13122 | LMGP01070009 | C18 |
| LysoPE(16:0)* | C21 H44 N O7 P | 6.284 |  |  | LMGP02050002 | C18 |
| LysoPE(18:0)* | C23 H48 N O7 P | 5.089 |  | HMDB11129 | LMGP02050038 | C18 |
| LysoPE(18:2)* | C23 H44 N O7 P | 2.557 |  | HMDB11477 |  | HILIC |
| MANNITOL** | C6 H14 O6 | 6.264 | 69-65-8 | HMDB00765 |  | HILIC |
| Meliternatin*- | C19 H14 O8 | 6.913 |  |  | LMPK12113025 | HILIC |
| N-acetyl L-alanine* | C5 H9 N O3 | 4.436 | 97-69-8 | HMDB00766 |  | HILIC |
| N-ACETYLSEROTONIN** | C12 H14 N2 O2 | 1.959 | 1210-83-9 | HMDB01238 |  | C18 |
| NEPSILON,NEPSILON,NEPSILON-TRIMETHYLLYSINE** | C9 H20 N2 O2 | 0.362 | 55528-53-5 |  |  | HILIC |
| Nudifloramide* | C7 H8 N2 O2 | 0.499 | 701-44-0 |  |  | HILIC |
| O5'-(L-Glutamyl-sulfamoyl)-adenosine*- | C15 H21 N7 O9 S | 6.792 |  |  |  | HILIC |
| Octadecanamide** | C18 H37 N O | 5.089 | 124-26-5 | HMDB34146 | LMFA08010003 | C18 |
| Octanoyl-L-carnitine** | C15 H29 N O4 | 1.959 | 25243-95-2 | HMDB00791 |  | C18 |
| Oleamide* | C18 H35 N O | 6.740 |  | HMDB02117 | LMFA08010004 | C18 |
| Oleic acid monoglyceride* | C21 H40 O4 | 1.732 | 25496-72-4 |  |  | C18 |
| OMEGA-HYDROXYDODECANOIC ACID** | C12 H24 O3 | 4.907 | 505-95-3 | HMDB02059 |  | C18 |
| PC(30:0)* | C38 H76 N O8 P | 2.297 |  | HMDB07965 | LMGP01010560 | C18 |
| PC(32:0)* | C40 H80 N O8 P | 1.014 | 2644-64-6 | HMDB00564 | LMGP01010564 | C18 |
| PC(34:1)* | C42 H82 N O8 P | 0.350 |  | HMDB07972 | LMGP01010005 | C18 |
| PC(34:2)* | C42 H80 N O8 P | 0.499 |  | HMDB07912 |  | HILIC |
| PC(34:2)* | C42 H80 N O8 P | 1.855 |  | HMDB07880 |  | HILIC |
| PC(34:2)* | C42 H80 N O8 P | 2.307 |  | HMDB07880 |  | HILIC |
| PC(36:1)* | C44 H86 N O8 P | 6.574 |  | HMDB08037 | LMGP01010750 | C18 |
| PC(36:2)* | C44 H84 N O8 P | 6.581 | 4235-95-4 | HMDB08070 | LMGP01010890 | C18 |
| PC(36:2)* | C44 H84 N O8 P | 2.623 |  |  |  | HILIC |
| PC(36:2)* | C44 H84 N O8 P | 6.956 |  | HMDB08071 |  | HILIC |
| PC(36:2)* | C44 H84 N O8 P | 5.378 |  | HMDB07888 |  | HILIC |
| PC(36:2)* | C44 H84 N O8 P | 4.699 |  | HMDB07888 |  | HILIC |
| PC(36:4)* | C44 H80 N O8 P | 4.662 |  | HMDB07889 |  | C18 |
| PC(36:5)* | C44 H78 N O8 P | 5.827 |  | HMDB07890 |  | C18 |
| PC(38:3)* | C46 H86 N O8 P | 4.992 |  | HMDB08020 |  | C18 |
| PC(38:6)* | C46 H80 N O8 P | 2.497 |  |  |  | HILIC |
| PC(O-16:0)* | C24 H52 N O6 P | 6.426 | 52691-62-0 |  | LMGP01060010 | C18 |
| PE(36:1)* | C41 H80 N O8 P | 5.429 |  | HMDB08993 | LMGP02010036 | C18 |
| PE(P-38:4)* | C43 H78 N O7 P | 5.115 |  | HMDB05779 | LMGP02030003 | C18 |
| Piperine* | C17 H19 N O3 | 0.555 | 94-62-2 | HMDB29377 |  | HILIC |
| Plastoquinol-1*- | C13 H18 O2 | 8.024 |  |  | LMPR02010037 | HILIC |
| Proline betaine* | C7 H13 N O2 | 7.406 | 471-87-4 | HMDB04827 |  | HILIC |
| Propionylcarnitine* | C10 H19 N O4 | 6.868 | 17298-37-2 | HMDB00824 |  | HILIC |
| Purine* | C5 H4 N4 | 1.287 |  |  |  | HILIC |
| PURINE** | C5 H4 N4 | 1.040 | 120-73-0 | HMDB01366 |  | C18 |
| PYRIDOXAL** | C8 H9 N O3 | 0.923 | 65-22-5 |  |  | HILIC |
| Pyridoxamine* | C7 H8 N2 O3 | 4.638 |  |  |  | HILIC |
| Pyroglutamic acid* | C5 H7 N O3 | 2.120 | 98-79-3 | HMDB00267 |  | HILIC |
| RETINOATE** | C20 H28 O2 | 5.086 | 302-79-4 | HMDB01852 |  | C18 |
| SEROTONIN** | C10 H12 N2 O | 1.255 | 153-98-0 |  |  | C18 |
| SM(d18:1/16:0)* | C39 H79 N2 O6 P | 3.661 |  | HMDB10169 | LMSP03010003 | HILIC |
| SM(d18:1/18:1)** | C41 H81 N2 O6 P | 0.972 |  |  | LMSP03010029 | C18 |
| SM(d18:1/24:0)* | C47 H95 N2 O6 P | 0.362 |  | HMDB11697 | LMSP03010008 | C18 |
| SM(d18:1/24:0)* | C47 H95 N2 O6 P | 4.891 |  | HMDB11697 | LMSP03010008 | C18 |
| SM(d18:1/24:1)** | C47 H93 N2 O6 P | 1.425 |  | HMDB12107 | LMSP03010000 | C18 |
| SPHINGOMYELIN (D18:1/18:0)** | C41 H83 N2 O6 P | 0.344 | 85187-10-6 | HMDB01348 |  | C18 |
| SPHINGOMYELIN (D18:1/18:0)** | C41 H83 N2 O6 P | 8.652 | 85187-10-6 | HMDB01348 |  | C18 |
| SQMG(16:1)*- | C25 H46 O11 S | 5.474 |  |  | LMGL04010002 | HILIC |
| TAURINE** | C2 H7 N O3 S | 0.936 | 107-35-7 | HMDB00251 |  | HILIC |
| Taurocholic acid*- | C26 H45 N O7 S | 6.595 |  |  |  | HILIC |
| Taurolithocholic acid 3-glucuronide*- | C32 H53 N O11 S | 3.898 |  | HMDB02429 | LMST05040009 | HILIC |
| Tetraethylene glycol* | C8 H18 O5 | 0.928 |  |  |  | HILIC |
| TG(48:2)* | C51 H94 O6 | 9.151 |  | HMDB42311 |  | C18 |
| TG(52:3)* | C55 H100 O6 | 5.831 |  | HMDB42604 |  | C18 |
| TG(52:4)* | C55 H98 O6 | 1.805 |  | HMDB44339 |  | C18 |
| THEOPHYLLINE** | C7 H8 N4 O2 | 0.392 | 58-55-9 | HMDB01889 |  | C18 |
| THEOPHYLLINE** | C7 H8 N4 O2 | 0.853 | 58-55-9 | HMDB01889 |  | HILIC |
| Trans-Epoxysuccinyl-L-leucylamido(4-guanidino)butane* | C15 H27 N5 O5 | 1.594 |  |  |  | C18 |
| Tryptophan* | C11 H12 N2 O2 | 1.591 |  | HMDB00929 |  | HILIC |
| Tyrosine* | C9 H11 N O3 | 2.327 | 60-18-4 | HMDB00158 |  | HILIC |
| Uric acid* | C5 H4 N4 O3 | 0.966 | 69-93-2 | HMDB00289 |  | HILIC |
| URIDINE** | C9 H12 N2 O6 | 1.594 | 58-96-8 | HMDB00296 |  | C18 |
| UROCANATE** | C6 H6 N2 O2 | 2.158 | 104-98-3 | HMDB00301 |  | HILIC |

**Supplementary Document S6: Putatively identified compounds based on accurate mass, isotope abundance and isotope distribution.** The table below shows the compound annotations for which purchased standards were not available. Names are based on accurate mass (< 10 ppm), isotopic abundance and isotopic distributions, followed by manual inspection of the spectra.

| **Compound** | **Formula** | **RT**  **(mins)** | **CAS ID** | **HMP ID** | **LMP ID** | **Frac-**  **tion** |
| --- | --- | --- | --- | --- | --- | --- |
| (+)-12-Methyl myristic acid | C15 H30 O2 | 2.443 | 5502-94-3 |  | LMFA01020008 | C18 |
| (23R)-3α,7α,12α,23-Tetrahydroxy-5β-cholan-24-oic Acid | C24 H40 O6 | 3.157 |  |  |  | C18 |
| (2R)-2-Hydroxy-2-methylbutanenitrile | C5 H9 N O | 0.354 |  | HMDB60309 |  | C18 |
| (3R,7R)-1,3,7-Octanetriol | C8 H18 O3 | 0.717 | 217650-11-8 | HMDB33625 |  | C18 |
| (6Z,9Z,12Z,15Z,18Z)-3-Oxotetracosapenta-6,9,12,15,18-enoyl-CoA | C45 H70 N7 O18 P3 S | 5.219 |  |  |  | C18 |
| (7R)-7-(4-Carboxybutanamido)cephalosporanate | C15 H18 N2 O8 S | 0.569 |  |  |  | C18 |
| *GlcNAcbeta1-3Galbeta1-4GlcNAcbeta1-3Galbeta1-4Glcbeta-Cer(d18:1/24:1) | C122 H213 N5 O63 | 4.703 |  |  |  | C18 |
| 1-(10-methylhexadecanyl)-2-(8-[3]-ladderane-octanyl)-sn-glycerophosphocholine | C45 H86 N O6 P | 5.823 |  |  | LMGP01040093 | C18 |
| 1-(beta-D-Glucopyranosyloxy)-3-octanone | C14 H26 O7 | 1.685 | 194919-40-9 | HMDB31315 |  | HILIC |
| 1-(O-alpha-D-glucopyranosyl)-(1,3R,25S,27R)-octacosanetetrol | C34 H68 O9 | 3.563 |  |  | LMFA13010014 | C18 |
| 1,2,3,6-Tetrakis-O-galloyl-beta-D-glucose | C34 H28 O22 | 6.351 | 79886-50-3 |  |  | C18 |
| 1,26-Hexacosanediol | C26 H54 O2 | 0.990 | 15541-01-2 | HMDB36581 |  | C18 |
| 10,11-Dihydro-12R-hydroxy-leukotriene E4 | C23 H39 N O6 S | 3.994 |  | HMDB12501 |  | HILIC |
| 10-Deacetyl-2-debenzoylbaccatin III | C22 H32 O9 | 6.369 |  |  |  | HILIC |
| 10-Hydroxycamtothecin | C20 H16 N2 O5 | 7.635 | 19685-09-7 |  |  | HILIC |
| 12(13)-EpOME | C18 H32 O3 | 0.520 |  | HMDB04702 | LMFA02000038 | HILIC |
| 12-hydroxyjasmonic acid | C12 H18 O4 | 0.854 |  |  | LMFA02020011 | HILIC |
| 12-hydroxyjasmonic acid | C12 H18 O4 | 0.853 |  |  | LMFA02020011 | HILIC |
| 12S-HHT | C17 H28 O3 | 2.563 | 54397-84-1 | HMDB12535 |  | C18 |
| 13Z,16Z-docosadienoic acid | C22 H40 O2 | 1.770 |  |  |  | C18 |
| 14,16-Nonacosanedione | C29 H56 O2 | 0.667 | 72089-30-6 | HMDB35234 |  | C18 |
| 15(S)-HEDE | C20 H36 O3 | 2.376 | 92693-04-4 |  |  | C18 |
| 15-HETE-T | C22 H37 N O5 S | 1.018 |  |  | LMFA08020149 | C18 |
| 15-Keto-prostaglandin F2a | C20 H32 O5 | 2.787 | 35850-13-6 | HMDB04240 |  | C18 |
| 17-hydroxyandrostane-3-glucuronide | C25 H40 O9 | 1.684 |  |  | LMST05010036 | C18 |
| 17-trifluoromethylphenyl trinor PGF2α methyl ester | C25 H33 F3 O5 | 1.121 | 195503-20-9 |  |  | HILIC |
| 18:2-Glc-Campesterol | C52 H88 O7 | 6.904 |  |  | LMST01031135 | C18 |
| 18-fluoro-9Z,12Z-octadecadienoic acid | C18 H31 F O2 | 2.439 |  |  | LMFA01090051 | C18 |
| 18-Oxocortisol | C21 H28 O6 | 0.746 |  |  | LMST02030194 | C18 |
| 18Z-Heptacosen-10-one | C27 H52 O | 2.796 |  |  | LMFA12000224 | C18 |
| 1alpha,23R,25S,26-Tetrahydroxyvitamin D3 | C27 H44 O5 | 1.308 |  |  | LMST03020674 | C18 |
| 1-Dehydro-9-fluoro-11-oxotestololactone | C19 H21 F O4 | 2.065 |  |  |  | C18 |
| 1-deoxy-tetradecasphinganine | C14 H31 N O | 1.070 |  |  | LMSP01080033 | C18 |
| 1-Hexacosene | C26 H52 | 4.857 |  |  | LMFA11000320 | C18 |
| 1-Hexadecyl-2-arachidonoyl-glycerol | C39 H70 O4 | 4.217 |  |  |  | C18 |
| 1-Hexadecyl-2-O-methyl-glycerol | C20 H42 O3 | 3.156 | 111188-59-1 |  |  | C18 |
| 1-Hexadecylamine | C16 H35 N | 4.699 | 143-27-1 |  |  | C18 |
| 1-Naphthaleneacetic acid | C12 H10 O2 | 0.330 | 86-87-3 | HMDB32708 |  | C18 |
| 1-O-alpha-D-glucopyranosyl-(2-hexadecanoyloxy)-eicosan-1-ol | C42 H82 O8 | 6.048 |  |  | LMFA13010004 | C18 |
| 1-O-p-Coumaroyl-(b-D-glucose 6-O-sulfate) | C15 H18 O11 S | 6.027 |  | HMDB41174 |  | C18 |
| 1-Phosphatidyl-D-myo-inositol | C11 H19 O13 P | 6.773 |  | HMDB06953 |  | HILIC |
| 2-(o-Carboxybenzamido)glutaramic acid | C13 H14 N2 O6 | 6.773 | 2393-39-7 |  |  | HILIC |
| 2,2',3',4,4',5,5'-Heptachloro-3-biphenylol | C12 H3 Cl7 O | 5.862 |  |  |  | C18 |
| 2,2-Bis(bromomethyl)propane-1,3-diol | C5 H10 Br2 O2 | 1.399 | 3296-90-0 |  |  | C18 |
| 2,3,3-tribromoacrylic acid | C3 H Br3 O2 | 3.262 |  |  | LMFA01090077 | C18 |
| 2',3'-Cyclic CMP | C9 H12 N3 O7 P | 3.975 | 633-90-9 |  |  | HILIC |
| 20:0 Campesteryl ester | C48 H86 O2 | 2.912 |  |  | LMST01020045 | C18 |
| 20-hexacosenal | C26 H50 O | 0.923 |  |  |  | HILIC |
| 22:3-Glc-cholesterol | C55 H92 O7 | 6.573 |  |  | LMST01010382 | C18 |
| 24-Nor-5beta-chol-22-ene-3alpha,7alpha,12alpha-triol | C23 H38 O3 | 2.911 |  |  | LMST04060012 | C18 |
| 24-Nor-5β-cholane-3α,7α,12α,22,23-pentol | C23 H40 O5 | 5.463 |  |  |  | C18 |
| 24-Propylcholestan-3-ol | C30 H54 O | 2.631 | 117675-19-1 | HMDB34814 |  | C18 |
| 25-Hydroxy-24-epi-brassinolide | C28 H48 O7 | 3.552 |  |  |  | C18 |
| 25-Hydroxycholecalciferol (25-hydroxyvitamin D3) | C27 H44 O2 | 6.905 | 19356-17-3 |  |  | C18 |
| 26,26,26-trifluoro-25-hydroxy-27-norvitamin D3 | C26 H39 F3 O2 | 16.641 |  |  | LMST03020024 | HILIC |
| 28:2(5Z,9Z)(6Br) | C28 H51 Br O2 | 1.436 |  |  | LMFA01030890 | C18 |
| 2-Amino-3-methyl-1-butanol | C5 H13 N O | 2.119 | 473-75-6 |  |  | HILIC |
| 2-Aminoethylphosphocholate | C26 H46 N O7 P | 1.319 |  |  |  | C18 |
| 2-Dodecylbenzenesulfonic acid | C18 H30 O3 S | 0.773 |  | HMDB31031 |  | HILIC |
| 2-Hydroxy-24-keto-octacosanolide | C28 H52 O4 | 3.266 |  |  | LMFA07040063 | C18 |
| 2-Hydroxylauroylcarnitine | C19 H37 N O5 | 3.265 |  | HMDB13164 |  | C18 |
| 2-Hydroxymyristoylcarnitine | C21 H41 N O5 | 3.515 |  | HMDB13166 |  | HILIC |
| 2-Octaprenyl-3-methyl-6-methoxy-1,4-benzoquinone | C48 H72 O3 | 3.155 |  |  |  | C18 |
| 2-Octaprenyl-6-methoxyphenol | C47 H72 O2 | 8.827 |  |  |  | C18 |
| 2-Stearyl citrate | C24 H44 O7 | 0.518 |  | HMDB39225 |  | C18 |
| 3, 5-Tetradecadiencarnitine | C21 H37 N O4 | 3.537 |  |  |  | HILIC |
| 3,17-Dihydroxypancuronium | C31 H56 N2 O2 | 1.180 | 43021-46-1 |  |  | HILIC |
| 3,4-Dihydroxymandelic acid | C8 H8 O5 | 6.773 | 775-01-9 | HMDB01866 |  | HILIC |
| 3,5,3',5'-Tetra-tert-butyldiphenoquinone | C28 H40 O2 | 5.015 | 2455-14-3 |  |  | C18 |
| 3',5'-Cyclic Inosine monophosphate (cIMP) | C10 H11 N4 O7 P | 6.808 | 3545-76-4 |  |  | HILIC |
| 3,5-Dihydroxyphenylacetyl-CoA | C29 H42 N7 O19 P3 S | 1.541 |  |  |  | C18 |
| 3,5-Pyridinedicarboxylic acid, 2,6-dimethyl-4-(3-nitrophenyl)-, mono(2-hydroxyethyl) ester | C17 H16 N2 O7 | 5.175 | 134283-05-9 |  |  | C18 |
| 3,7,11,15,18,22,26,30-octamethyldotriacontane-1,32-diol | C40 H82 O2 | 4.364 |  |  | LMFA05000084 | C18 |
| 3a,7a-Dihydroxy-5b-24-oxocholestanoyl-CoA | C48 H78 N7 O20 P3 S | 5.910 |  | HMDB06896 |  | C18 |
| 3a-Hydroxy-5b-pregnane-20-one | C21 H34 O2 | 3.266 |  | HMDB06759 |  | C18 |
| 3alpha-androstanediol glucuronide | C25 H40 O8 | 1.014 |  | HMDB10339 | LMST05010004 | C18 |
| 3-Buten-1-amine | C4 H9 N | 3.216 |  |  |  | HILIC |
| 3-Decaprenyl-4,5-dihydroxybenzoate | C57 H86 O4 | 6.560 |  | HMDB60249 |  | C18 |
| 3-Hexadecanoyloleanolic acid | C46 H78 O4 | 5.026 | 19833-14-8 | HMDB36967 |  | C18 |
| 3-hydroxy-undecanoic acid | C11 H22 O3 | 1.107 |  |  | LMFA01050162 | C18 |
| 3-ketosphingosine | C18 H35 N O2 | 1.277 | 19767-16-9 |  |  | C18 |
| 3-Methyl-19-nor-17alpha-pregna-1,3,5(10)-trien-17-ol | C21 H30 O | 2.441 |  |  |  | C18 |
| 3-Methylbutyraldehyde oxime | C5 H11 N O | 0.351 | 626-90-4 |  |  | C18 |
| 3-O-(Rhaa1-2(Glcb1-4)Glcb)-(25R)-spirost-5en-3beta-ol | C46 H76 O17 | 4.198 |  |  | LMST01080090 | C18 |
| 3-O-beta-D-glucosyl-brassicasterol | C34 H56 O6 | 2.560 |  |  |  | C18 |
| 3-O-Sulfogalactosylceramide (d18:1/18:1) | C42 H79 N O11 S | 5.540 |  | HMDB12317 |  | C18 |
| 3-O-trans-Feruloyleuscaphic acid | C40 H56 O8 | 1.014 |  | HMDB31837 |  | C18 |
| 3-Oxo-5,6-dehydrosuberyl-CoA semialdehyde | C29 H44 N7 O19 P3 S | 1.541 |  |  |  | C18 |
| 3-Oxo-docosa-7,10,13,16,19-all-cis-pentaenoyl-CoA | C43 H66 N7 O18 P3 S | 4.701 |  | HMDB60200 |  | C18 |
| 4-(3-Hydroxybutyl)-3,3,5-trimethylcyclohexanone | C13 H24 O2 | 2.863 | 128660-70-8 | HMDB39805 |  | C18 |
| 4-(Methylnitrosamino)-1-(3-pyridyl)-1-butanone | C10 H13 N3 O2 | 0.725 | 64091-91-4 | HMDB11603 |  | C18 |
| 4,4'-Diapolycopene | C30 H40 | 3.269 |  |  | LMPR01070143 | C18 |
| 4,7-Didehydroneophysalin B | C28 H28 O9 | 1.129 | 134461-76-0 | HMDB39695 |  | HILIC |
| 4-Aminobutyraldehyde | C4 H9 N O | 0.514 | 4390-05-0 | HMDB01080 |  | HILIC |
| 4-Aminoethylbenzenesulfonyl Fluoride | C8 H10 F N O2 S | 1.867 | 30827-99-7 |  |  | HILIC |
| 4-Guanidinobutanoic acid | C5 H11 N3 O2 | 0.602 | 463-00-3 | HMDB03464 |  | HILIC |
| 4-Hydroxycinnamoylmethane | C10 H10 O2 | 1.198 | 3160-35-8 |  |  | C18 |
| 5-(3,4-Diacetoxybut-1-ynyl)-2,2'-bithiophene | C16 H14 O4 S2 | 6.779 |  |  |  | HILIC |
| 5-(L-alanin-3-yl)-2-hydroxy-cis,cis-muconate 6-semialdehyde | C9 H11 N O6 | 6.925 |  |  | LMFA01170073 | HILIC |
| 5,3'-Dihydroxy-6,7,4',5'-tetramethoxyflavanone | C19 H20 O8 | 0.388 |  |  | LMPK12140638 | C18 |
| 5,9,17-hexacosatrienoic acid | C26 H46 O2 | 4.718 |  |  | LMFA01030426 | C18 |
| 5,9-Hexacosadiene | C26 H50 | 4.719 |  |  | LMFA11000452 | C18 |
| 5alpha-Androstane-2alpha-fluoro-17beta-ol-3-one acetate | C21 H31 F O3 | 1.014 |  |  |  | C18 |
| 5-beta-Androsterone | C19 H30 O2 | 1.015 | 53-42-9 | HMDB00490 | LMST02020059 | C18 |
| 5beta-Pregnane-3alpha,17alpha,20alpha-triol | C21 H36 O3 | 2.782 | 1098-45-9 |  |  | C18 |
| 5-Diphosphoinositol pentakisphosphate | C6 H19 O27 P7 | 5.169 |  | HMDB06229 |  | C18 |
| 5-Heptadecyl-1,3-benzenediol | C23 H40 O2 | 3.272 | 41442-57-3 | HMDB38530 |  | C18 |
| 5-Hydroxyisourate | C5 H4 N4 O4 | 4.670 |  |  |  | HILIC |
| 5-Hydroxyisourate | C5 H4 N4 O4 | 4.668 |  |  |  | HILIC |
| 5-Hydroxyriluzole | C8 H5 F3 N2 O2 S | 3.917 | 179070-92-9 |  |  | HILIC |
| 5-Taurinomethyl-2-thiouridine | C12 H19 N3 O8 S2 | 5.744 | 497258-54-5 | HMDB11610 |  | C18 |
| 6beta-(Dimethylamino)-3beta,5-dihydroxy-5alpha-pregnan-20-one | C23 H39 N O3 | 4.718 |  |  |  | C18 |
| 6-Oxabicyclo[3.1.0]hexane-2-undecanoic acid methyl ester | C17 H30 O3 | 0.757 |  |  |  | C18 |
| 7 alpha-Hydroxy-3-oxo-4-cholestenoate | C27 H42 O4 | 1.308 | 115538-85-7 | HMDB12458 |  | C18 |
| 7,7'-Dihydroxy-6,8'-bicoumarin | C18 H10 O6 | 6.719 | 15575-52-7 | HMDB33559 |  | HILIC |
| 7-Hydroxy-3-oxocholanoic acid | C24 H38 O4 | 16.360 | 77059-13-3 | HMDB00460 |  | HILIC |
| 7-keto-stearic acid | C18 H34 O3 | 3.562 |  |  | LMFA02000244 | C18 |
| 7-Oxostigmasterol | C29 H46 O2 | 0.543 | 36449-99-7 | HMDB30015 |  | HILIC |
| 7Z-Docosene | C22 H44 | 16.240 |  |  | LMFA11000091 | HILIC |
| 8',10'-Dihydroxydihydroergotamine | C33 H39 N5 O5 | 1.575 |  |  |  | C18 |
| 8-oxo-Resolvin D1 | C22 H30 O5 | 2.065 |  |  | LMFA04000079 | C18 |
| 8Z,11Z,14Z-heptadecatrienoic acid | C17 H28 O2 | 2.451 |  |  |  | C18 |
| 9,10-DiHOME | C18 H34 O4 | 2.027 |  | HMDB04704 | LMFA02000229 | C18 |
| 9-Decenoylcarnitine | C17 H31 N O4 | 3.690 |  | HMDB13205 |  | HILIC |
| AAPH | C8 H18 N6 | 2.417 | 2997-92-4 |  |  | C18 |
| Acacetin 7-[2'''-(2-methylbutyryl)rutinoside] | C33 H40 O15 | 0.817 |  |  | LMPK12110454 | C18 |
| Adenosine tetraphosphate | C10 H17 N5 O16 P4 | 5.732 | 58337-43-2 | HMDB01364 |  | C18 |
| Adenylyl-molybdopterin | C20 H26 N10 O12 P2 S2 | 4.754 |  | HMDB59628 |  | C18 |
| Ala Glu Val | C13 H23 N3 O6 | 0.519 |  |  |  | HILIC |
| alatolide | C19 H26 O6 | 0.927 |  |  |  | HILIC |
| All trans decaprenyl diphosphate | C50 H90 O7 P2 | 6.223 |  | HMDB06288 |  | C18 |
| alpha,beta-Methylene ATP | C11 H18 N5 O12 P3 | 3.837 |  |  |  | HILIC |
| alpha-Amyrin cerotate | C56 H100 O2 | 5.089 | 307319-21-7 | HMDB36744 |  | C18 |
| Alpha-CEHC | C16 H22 O4 | 1.336 | 4072-32-6 | HMDB01518 |  | C18 |
| Alpha-N-Phenylacetyl-L-glutamine | C13 H16 N2 O4 | 1.020 | 28047-15-6 | HMDB06344 |  | HILIC |
| alpha-tocopherol | C29 H50 O2 | 5.358 |  | HMDB01893 | LMPR02020001 | C18 |
| AMC Arachidonoyl Amide | C30 H39 N O3 | 1.256 |  |  |  | C18 |
| Amprenavir | C25 H35 N3 O6 S | 0.664 | 161814-49-9 |  |  | C18 |
| Androstan-3α,17β-diol | C19 H32 O2 | 3.153 |  |  |  | C18 |
| Annoglaxin | C35 H62 O8 | 6.018 |  | HMDB33603 |  | C18 |
| Annotinine | C16 H21 N O3 | 6.405 |  |  |  | HILIC |
| ANTU | C11 H10 N2 S | 1.246 | 86-88-4 |  |  | HILIC |
| Apigenin 7,4'-diglucuronide | C27 H26 O17 | 6.719 |  |  | LMPK12110361 | HILIC |
| Arachidonoylmorpholine | C24 H39 N O2 | 1.872 |  |  | LMFA08020052 | C18 |
| Arg Arg Lys | C18 H38 N10 O4 | 1.443 |  |  |  | C18 |
| Arg Val His | C17 H30 N8 O4 | 1.015 |  |  |  | C18 |
| Asn Tyr His | C19 H24 N6 O6 | 3.855 |  |  |  | HILIC |
| Aspidospermatine | C21 H26 N2 O2 | 1.184 |  | HMDB30358 |  | C18 |
| Avermectin A2a | C49 H76 O15 | 5.017 |  |  | LMPK04000023 | C18 |
| Azinphos Methyl | C10 H12 N3 O3 P S2 | 6.790 | 86-50-0 |  |  | HILIC |
| Balsalazide | C17 H15 N3 O6 | 0.703 | 80573-04-2 | HMDB15149 |  | C18 |
| Begacestat | C9 H8 Cl F6 N O3 S2 | 5.087 |  |  |  | C18 |
| Biguanide | C2 H7 N5 | 11.113 | 56-03-1 |  |  | HILIC |
| Biphenyl | C12 H10 | 1.291 | 92-52-4 | HMDB34437 |  | HILIC |
| Bis(2-chloroethyl)ether | C4 H8 Cl2 O | 0.321 | 111-44-4 |  |  | C18 |
| Bismurrayafoline E | C48 H56 N2 O4 | 1.014 | 252350-80-4 | HMDB37741 |  | C18 |
| Bouillonamide A | C46 H67 N5 O8 | 4.650 |  |  |  | C18 |
| Brevifolincarboxylic acid 9-sulfate | C13 H8 O11 S | 5.086 |  | HMDB31592 |  | C18 |
| Butenoyl PAF | C28 H57 N O7 P | 3.301 |  |  |  | C18 |
| Butyl oleate sulfate | C22 H42 O6 S | 2.416 | 38621-44-2 | HMDB37511 |  | C18 |
| Butyryl-L-carnitine | C11 H22 N O4 | 5.090 | 25576-40-3 |  |  | HILIC |
| C.I. Food Black 1 | C28 H21 N5 O14 S4 | 1.400 | 2519-30-4 | HMDB33389 |  | C18 |
| C16 Sphinganine-1-phosphate | C16 H36 N O5 P | 1.014 |  |  | LMSP01050006 | C18 |
| C16 Sulfatide | C40 H77 N O11 S | 5.509 |  |  | LMSP06020002 | C18 |
| C16-OH Sulfatide | C40 H77 N O12 S | 5.217 |  |  | LMSP06020003 | C18 |
| C20-OHSulfatide | C44 H85 N O12 S | 6.280 |  |  | LMSP06020005 | C18 |
| C22 Sulfatide | C46 H89 N O11 S | 6.232 |  |  | LMSP06020009 | C18 |
| C22-PGF4alpha | C22 H34 O5 | 2.925 |  |  | LMFA04000046 | C18 |
| C24:1 Sulfatide | C48 H91 N O11 S | 6.220 |  |  | LMSP06020015 | C18 |
| Caffeine | C8 H10 N4 O2 | 0.448 | 58-08-2 | HMDB01847 |  | C18 |
| Calcium Gluceptate | C14 H26 Ca O16 | 1.541 | 29039-00-7 | HMDB14471 |  | C18 |
| Campesteryl glucoside | C34 H58 O6 | 2.816 |  |  | LMST01031126 | C18 |
| Capric acid | C10 H20 O2 | 1.297 |  | HMDB00511 | LMFA01010010 | C18 |
| Capsiamide | C17 H35 N O | 2.381 | 64317-66-4 | HMDB40940 |  | C18 |
| Captafol | C10 H9 Cl4 N O2 S | 5.023 | 6/1/2425 | HMDB31669 |  | C18 |
| Carboxyterbinafine | C21 H23 N O2 | 0.533 | 99473-14-0 |  |  | HILIC |
| CE(18:1) | C45 H78 O2 | 4.364 |  | HMDB05189 |  | C18 |
| CE(20:4) | C47 H76 O2 | 8.928 | 604-34-2 | HMDB06726 |  | C18 |
| Cer(d18:0/22:0) | C40 H81 N O3 | 7.310 |  | HMDB11765 |  | C18 |
| Ceramide (d18:1/16:0) | C34 H67 N O3 | 5.722 | 104404-17-3 | HMDB04949 |  | C18 |
| Ceramide (d18:1/18:0) | C36 H71 N O3 | 6.283 | 104404-17-3 | HMDB04950 |  | C18 |
| Ceramide (d18:1/20:0) | C38 H75 N O3 | 6.808 | 104404-17-3 | HMDB04951 |  | C18 |
| Ceramide (d18:1/24:1) | C42 H81 N O3 | 7.266 | 104404-17-3 | HMDB04953 |  | C18 |
| Ceramide(d14:1(4E)/22:0(2OH)) | C36 H71 N O4 | 4.767 |  |  | LMSP02010070 | C18 |
| Ceramide(d15:1/20:0) | C35 H69 N O3 | 6.010 |  |  | LMSP02010043 | C18 |
| Ceramide(d16:1/23:0) | C39 H77 N O3 | 7.031 |  |  | LMSP02010017 | C18 |
| Ceramide(d18:0/24:1) | C42 H83 N O3 | 7.595 |  | HMDB11769 | LMSP02020011 | C18 |
| Ceramide(d18:1/17:0) | C35 H69 N O3 | 6.011 |  |  | LMSP02010020 | C18 |
| Ceramide(d18:2/22:0) | C40 H77 N O3 | 6.869 |  |  | LMSP02010029 | C18 |
| Ceramide(d18:2/23:0) | C41 H79 N O3 | 7.086 |  |  | LMSP02010030 | C18 |
| Ceramide(t18:0/16:0(2OH)) | C34 H69 N O5 | 2.837 |  |  | LMSP02030015 | C18 |
| Ceramide(t18:0/18:0(2OH)) | C36 H73 N O5 | 6.892 |  |  | LMSP02030016 | C18 |
| Ceramide(t18:0/20:0(2OH)) | C38 H77 N O5 | 7.312 |  |  | LMSP02030017 | C18 |
| Cerebroside C | C43 H79 N O9 | 5.902 | 98677-33-9 |  |  | C18 |
| CerP(d18:1/14:0) | C32 H64 N O6 P | 5.870 |  |  | LMSP02050013 | C18 |
| CerP(d18:1/24:1) | C42 H82 N O6 P | 5.630 |  |  | LMSP02050007 | C18 |
| Chenodeoxycholic acid | C24 H40 O4 | 5.361 | 474-25-9 | HMDB00518 |  | C18 |
| Chenodeoxyglycocholate | C26 H43 N O5 | 1.060 |  | HMDB06898 | LMST05030003 | C18 |
| Chenodeoxyglycocholic acid | C26 H43 N O5 | 1.060 |  | HMDB06898 |  | C18 |
| Cheritamine | C40 H68 N2 O | 3.266 | 223696-37-5 | HMDB34872 |  | C18 |
| Chloraniformethan | C9 H7 Cl5 N2 O | 4.918 | 20856-57-9 |  |  | C18 |
| Chlorobactane | C40 H74 | 0.588 |  |  | LMPR01070287 | C18 |
| Chlorthiophos | C11 H15 Cl2 O3 P S2 | 4.072 | 21923-23-9 |  |  | HILIC |
| Cholesteryl linoleate | C45 H76 O2 | 9.238 | 604-33-1 | HMDB00610 |  | C18 |
| CL(1'-[18:2/0:0],3'-[18:2/0:0]) | C45 H82 O15 P2 | 4.617 |  |  | LMGP12070001 | C18 |
| CL(1'-[22:1/22:1],3'-[22:1/14:1]) | C89 H166 O17 P2 | 5.339 |  |  | LMGP12010005 | C18 |
| CL(16:0/16:0/18:1/18:1) | C77 H146 O17 P2 | 4.159 |  | HMDB56431 |  | C18 |
| CL(16:0/18:0/18:0/18:0) | C79 H154 O17 P2 | 5.023 |  | HMDB56497 |  | C18 |
| CL(16:0/18:2/20:4/20:4) | C84 H144 O17 P2 | 1.541 |  | HMDB56724 |  | C18 |
| CL(18:0/18:0/18:0/22:5) | C85 H156 O17 P2 | 4.922 |  | HMDB56967 |  | C18 |
| CLIOXANIDE | C15 H10 Cl I2 N O3 | 6.913 |  |  |  | HILIC |
| Cohibin C | C37 H68 O4 | 4.572 |  | HMDB35397 |  | C18 |
| Cohibin D | C37 H68 O4 | 4.034 |  | HMDB35398 |  | C18 |
| Concanamycin A | C46 H75 N O14 | 5.045 | 80890-47-7 |  |  | C18 |
| CPX | C16 H24 N4 O2 | 4.757 | 102146-07-6 |  |  | HILIC |
| Cucurbitacin O | C30 H46 O7 | 0.450 |  |  | LMST01010113 | C18 |
| Cucurbitacin P | C30 H48 O7 | 1.399 |  |  | LMST01010114 | C18 |
| Curcumin II | C22 H22 O5 | 0.927 | 91884-87-6 | HMDB39610 |  | HILIC |
| Cyanidin | C15 H11 Cl O6 | 6.794 | 528-58-5 | HMDB02708 |  | HILIC |
| Cyanidin 3-glucoside | C21 H21 O11 | 6.729 | 7084-24-4 | HMDB30684 |  | HILIC |
| Cyanidin 3-O-dimalonyl-laminaribioside | C33 H35 O22 | 5.087 |  | HMDB29242 |  | C18 |
| Cyanidin 3-sambubioside | C26 H29 O15 | 1.013 | 33012-73-6 | HMDB37976 |  | C18 |
| Cyclic dAMP | C10 H12 N5 O5 P | 6.773 | 1157-33-1 |  |  | HILIC |
| Cyclic GMP-AMP | C20 H24 N10 O13 P2 | 0.419 |  | HMDB60465 |  | HILIC |
| Cyclofoetoside B | C47 H80 O18 | 4.527 | 108333-83-1 |  |  | C18 |
| Cyclopamine | C27 H41 N O2 | 3.267 | 4449-51-8 |  |  | C18 |
| Cytidine diphosphate | C9 H15 N3 O11 P2 | 5.567 | 63-38-7 |  |  | C18 |
| D-Alanyl-D-serine | C6 H12 N2 O4 | 7.517 |  |  |  | HILIC |
| Decaprenoxanthin | C50 H72 | 3.153 |  |  | LMPR01080008 | C18 |
| Dehydrovomifoliol | C13 H18 O3 | 1.015 | 15764-81-5 | HMDB36819 |  | C18 |
| Delphinine | C33 H45 N O9 | 3.898 | 561-07-9 |  |  | HILIC |
| Demethylphylloquinone | C30 H44 O2 | 0.518 |  | HMDB04649 |  | C18 |
| Deoxymiroestrol | C20 H22 O5 | 1.197 |  |  |  | C18 |
| Deoxythymidine 5'-diphosphate (dTDP) | C10 H16 N2 O11 P2 | 5.080 | 491-97-4 |  |  | C18 |
| DG(13:0/22:6) | C38 H62 O5 | 5.908 |  |  | LMGL02010377 | C18 |
| DG(16:0/18:1) | C37 H70 O5 | 6.742 |  | HMDB07102 | LMGL02010006 | C18 |
| DG(16:0/20:2) | C39 H72 O5 | 6.782 |  | HMDB07109 |  | C18 |
| DG(16:0/20:40) | C39 H68 O5 | 6.740 |  | HMDB07112 |  | C18 |
| DG(16:1/20:3) | C39 H68 O5 | 6.050 |  | HMDB07140 |  | C18 |
| DG(16:1/20:5) | C39 H64 O5 | 5.943 |  | HMDB07143 |  | C18 |
| DG(17:2/22:0) | C42 H78 O5 | 4.159 |  |  | LMGL02010134 | C18 |
| DG(18:0/19:0) | C40 H78 O5 | 4.160 |  |  | LMGL02010065 | C18 |
| DG(18:0/20:5) | C41 H70 O5 | 6.783 |  | HMDB07172 |  | C18 |
| DG(18:1/18:1) | C39 H72 O5 | 6.784 |  | HMDB07218 | LMGL02010049 | C18 |
| DG(18:2/18:2) | C39 H68 O5 | 6.047 |  |  | LMGL02010063 | C18 |
| DG(18:2/18:3) | C39 H66 O5 | 6.365 |  | HMDB07250 |  | C18 |
| DG(19:0/20:0) | C42 H82 O5 | 4.857 |  |  | LMGL02010107 | C18 |
| DG(22:0/24:0) | C49 H96 O5 | 2.437 |  | HMDB07615 |  | C18 |
| DG(O-16:0/18:1) | C37 H72 O4 | 4.765 |  |  | LMGL02020001 | C18 |
| Dibenzo-p-dioxin | C12 H8 O2 | 0.353 | 262-12-4 |  |  | C18 |
| didesmethyl tocotrienol | C25 H36 O2 | 4.519 |  |  | LMPR02020058 | C18 |
| Diethylcarbamazine | C10 H21 N3 O | 2.932 | 90-89-1 |  |  | C18 |
| Dihydro-NAME | C21 H18 O8 | 0.443 |  |  |  | C18 |
| Dihydrotetrabenazine | C19 H29 N O3 | 2.301 | 3466-75-9 |  |  | C18 |
| Diisopropyl phosphate | C6 H15 O4 P | 1.070 |  |  |  | HILIC |
| Dimethylallyl diphosphate (DMAPP) | C5 H12 O7 P2 | 3.903 | 1186-30-7 |  | LMPR01010001 | HILIC |
| Dimexano | C4 H6 O2 S4 | 4.137 | 1468-37-7 |  |  | HILIC |
| Diphthamide | C13 H24 N5 O3 | 2.253 |  | HMDB60473 |  | C18 |
| Diplodiatoxin | C18 H28 O4 | 3.159 | 41060-01-9 | HMDB31471 |  | C18 |
| Docetaxel M2 | C43 H53 N O15 | 3.897 |  |  |  | HILIC |
| Docosanol | C22 H46 O | 3.266 | 30303-65-2 | HMDB14770 |  | C18 |
| dodecanamide | C12 H25 N O | 1.322 |  |  | LMFA08010001 | C18 |
| Dodecanoylcarnitine | C19 H37 N O4 | 3.589 | 25518-54-1 | HMDB02250 |  | HILIC |
| Dodecaprenyl diphosphate | C60 H100 O7 P2 | 1.583 |  | HMDB12217 |  | C18 |
| Dolichoic acid-[18-20] | C25 H42 O2 | 5.858 |  |  | LMPR03070025 | C18 |
| Dolichyl-19 phosphate | C95 H157 O4 P | 4.843 |  |  | LMPR03080001 | C18 |
| Dynemicin A | C30 H19 N O9 | 1.013 | 124412-57-3 |  |  | C18 |
| Echinenone/ (Myxoxanthin) | C40 H54 O | 3.287 |  |  | LMPR01070060 | C18 |
| Eicosanedioic acid | C20 H38 O4 | 4.906 |  |  | LMFA01170035 | C18 |
| Emblicanin B | C34 H20 O22 | 5.660 | 180465-45-6 | HMDB30436 |  | C18 |
| Epoxymurin A | C35 H62 O3 | 3.295 | 151484-64-9 | HMDB40920 |  | C18 |
| erythro-6,8-Tritriacontanediol | C33 H68 O2 | 1.541 | 155800-90-1 | HMDB41072 |  | C18 |
| EtN-P-6ManAlpha1-2ManAplha1-6ManAlpha1-4GlcNAplha1-6-PI(14:0/14:0) | C64 H121 N2 O35 P2 | 5.869 |  |  | LMGP15010001 | C18 |
| Fast green FCF | C37 H36 N2 O10 S3 | 6.347 | 2353-45-9 | HMDB38220 |  | C18 |
| Flavomycin | C69 H107 N4 O35 P | 5.083 | 11015-37-5 |  |  | C18 |
| Fluo-3 | C36 H30 Cl2 N2 O13 | 5.823 | 123632-39-3 |  |  | C18 |
| Formylmethionyl-leucyl-phenylalanine methyl ester | C22 H33 N3 O5 S | 0.667 | 65929-03-5 |  |  | C18 |
| Fortimicin AP | C14 H30 N4 O5 | 3.267 |  |  |  | C18 |
| Fructoselysine | C12 H24 N2 O7 | 7.826 |  |  |  | HILIC |
| Fuca1-2Galb1-3GalNAcb1-4(NeuGca2-3)Galb1-4Glcb-Cer(d18:1/18:0) | C79 H141 N3 O36 | 3.821 |  |  |  | HILIC |
| Fucalpha2-3GlcNAcbeta1-6GalNAcbeta1-3Galalpha1-4Galbeta1-4Glcbeta-Cer(d18:1/22:0) | C80 H145 N3 O32 | 1.960 |  |  |  | C18 |
| Fumonisin B1 | C34 H59 N O15 | 4.703 | 116355-83-0 |  | LMSP01080022 | C18 |
| Galabiosylceramide (d18:1/24:1) | C54 H101 N O13 | 6.370 | 77538-38-6 | HMDB04837 |  | C18 |
| Galalpha1-4Galbeta1-4Glcbeta-Cer(d18:1/26:1) | C62 H115 N O18 | 5.005 |  |  |  | C18 |
| Galb1-3(NeuAca2-6)(Fuca1-4)GlcNAcb1-3Galb1-4Glcb-Cer(d18:1/16:0) | C77 H137 N3 O35 | 1.402 |  |  |  | C18 |
| GalNAcb1-4(NeuGca2-3)Galb1-4Glcb-Cer(d18:1/24:1) | C74 H133 N3 O27 | 1.541 |  |  |  | C18 |
| GalNAcβ1-4GlcNAcβ-Sp | C18 H31 N5 O11 | 0.798 |  |  |  | C18 |
| gamma-L-Glutamyl-L-methionine sulfoxide | C10 H18 N2 O6 S | 4.134 | 39015-69-5 | HMDB38613 |  | HILIC |
| Ganglioside GA1 (d18:1/16:0) | C60 H110 N2 O23 | 4.201 | 71012-19-6 | HMDB04902 |  | C18 |
| Ganglioside GA2 (d18:1/25:0) | C63 H118 N2 O18 | 4.981 | 88506-68-7 | HMDB04898 |  | C18 |
| Ganglioside GA2 (d18:1/26:1) | C64 H118 N2 O18 | 1.015 | 88506-68-7 | HMDB04895 |  | C18 |
| Ganglioside GD3 (d18:0/18:0) | C70 H127 N3 O29 | 5.186 |  | HMDB11859 |  | C18 |
| Ganglioside GD3 (d18:0/22:0) | C74 H135 N3 O29 | 5.828 |  | HMDB11863 |  | C18 |
| Ganglioside GM1 (18:1/22:0) | C77 H139 N3 O31 | 1.401 |  | HMDB04858 |  | C18 |
| Ganglioside GM2 (d18:0/22:0) | C72 H132 N2 O26 | 5.177 |  | HMDB11901 |  | C18 |
| Ganglioside GM2 (d18:1/22:0) | C71 H129 N3 O26 | 5.177 | 104443-57-4 | HMDB04941 |  | C18 |
| Ganglioside GQ1c (d18:1/22:0) | C111 H191 N5 O55 | 6.559 |  | HMDB11954 |  | C18 |
| Ganoderic acid S | C32 H48 O5 | 1.396 |  | HMDB35313 |  | C18 |
| Geranylgeraniol | C20 H34 O | 3.560 | 24034-73-9 |  |  | C18 |
| Ginsenoyne B | C17 H23 Cl O2 | 7.431 | 139035-29-3 | HMDB39134 |  | HILIC |
| Glacin B | C35 H64 O7 | 2.440 |  | HMDB32091 |  | C18 |
| GlcCer(d14:1/22:0) | C42 H81 N O8 | 5.402 |  |  |  | C18 |
| GlcCer(d14:2(4E,6E)/20:0) | C40 H75 N O8 | 4.714 |  |  |  | C18 |
| GlcCer(d14:2(4E,6E)/22:0(2OH)) | C42 H79 N O9 | 5.172 |  |  |  | C18 |
| GlcCer(d15:1/18:0) | C39 H75 N O8 | 5.479 |  |  |  | C18 |
| GlcNAcbeta1-3Galbeta1-4Glcbeta-Cer(d18:1/26:1) | C64 H118 N2 O18 | 1.015 |  |  |  | C18 |
| GlcNAcbeta1-3Galbeta1-4GlcNAcbeta1-3Galbeta1-4Glcbeta-Cer(d18:1/26:1) | C78 H141 N3 O28 | 1.553 |  |  |  | C18 |
| GlcNAcbeta1-6GalNAcbeta1-3Galalpha1-4Galbeta1-4Glcbeta-Cer(d18:1/24:1 | C76 H137 N3 O28 | 1.541 |  |  |  | C18 |
| GlcNAcbeta1-6GalNAcbeta1-3Galalpha1-4Galbeta1-4Glcbeta-Cer(d18:1/26:1) | C78 H141 N3 O28 | 1.399 |  |  |  | C18 |
| Glu Glu Pro | C15 H23 N3 O8 | 3.898 |  |  |  | HILIC |
| Glucosylceramide (d18:1/18:0) | C42 H81 N O8 | 3.679 | 85305-87-9 | HMDB04972 |  | C18 |
| Glucosylceramide (d18:1/20:0) | C44 H85 N O8 | 5.594 | 85305-87-9 | HMDB04973 |  | C18 |
| Glutarylcarnitine | C12 H21 N O6 | 8.455 |  | HMDB13130 |  | HILIC |
| Gly His Lys | C14 H24 N6 O4 | 1.011 |  |  |  | C18 |
| Glycerol tripropanoate | C12 H20 O6 | 0.571 | 139-45-7 | HMDB32857 |  | HILIC |
| Glycodeoxycholic acid | C26 H43 N O5 | 1.097 |  |  | LMST05030006 | HILIC |
| Glycylproline | C7 H12 N2 O3 | 0.621 | 704-15-4 | HMDB00721 |  | C18 |
| Gossypetin 8-glucuronide-3-sulfate | C21 H18 O17 S | 3.812 |  |  | LMPK12113221 | HILIC |
| Guanosine 3',5'-bis(diphosphate) | C10 H17 N5 O17 P4 | 6.000 |  | HMDB59638 |  | C18 |
| Hedamycin | C41 H50 N2 O11 | 5.659 | 11048-97-8 |  |  | C18 |
| Hemibrevetoxin B | C28 H42 O7 | 1.448 | 122271-91-4 |  |  | C18 |
| hentriacontan-1-ol | C31 H64 O | 0.854 |  |  | LMFA05000096 | HILIC |
| Heptadecan-2-ol | C17 H36 O | 3.265 |  |  | LMFA05000531 | C18 |
| Heterophylliin F | C68 H50 O44 | 5.705 |  | HMDB32720 |  | C18 |
| Hexachlorobenzene | C6 Cl6 | 1.013 | 118-74-1 |  |  | C18 |
| Hexacosanoyl carnitine | C33 H65 N O4 | 4.959 |  | HMDB06347 |  | C18 |
| Hexacosyl-palmitate | C42 H84 O2 | 1.015 |  |  | LMFA07010012 | C18 |
| Hexanoyl-CoA | C27 H46 N7 O17 P3 S | 6.569 | 5060-32-2 | HMDB02845 |  | C18 |
| Histidinyl-Glutamine | C11 H17 N5 O4 | 1.689 |  | HMDB28883 |  | HILIC |
| hydroxyl dihexosylceramide (d18:1/18:0) | C48 H91 N O14 | 7.192 |  |  |  | C18 |
| Hydroxyphthioceranic acid (C34) | C34 H68 O3 | 2.909 |  |  | LMFA01020322 | C18 |
| Imidazoleacetic acid ribotide | C10 H15 N2 O9 P | 0.419 | 2888-19-9 | HMDB06032 |  | HILIC |
| Indole-3-carboxilic acid-O-sulphate | C9 H7 N O5 S | 1.402 |  | HMDB60002 |  | C18 |
| Indospicine | C7 H15 N3 O2 | 5.146 | 16377-00-7 |  |  | HILIC |
| Indoxyl | C8 H7 N O | 0.280 | 480-93-3 | HMDB04094 |  | HILIC |
| Iopamidol | C17 H22 I3 N3 O8 | 5.843 |  |  |  | C18 |
| Iopentol | C20 H28 I3 N3 O9 | 6.562 | 89797-00-2 | HMDB41909 |  | C18 |
| Isoeruboside B | C51 H84 O24 | 1.399 | 186545-52-8 | HMDB29769 |  | C18 |
| Isopentenyl pyrophosphate | C5 H12 O7 P2 | 3.848 | 147385-63-5 | HMDB01347 | LMPR01010008 | HILIC |
| Kahalalide F | C75 H124 N14 O16 | 5.662 | 149204-42-2 |  |  | C18 |
| Kentsin | C21 H40 N8 O6 | 3.896 | 56767-30-7 | HMDB05776 |  | HILIC |
| Labadoside | C38 H42 O16 | 1.541 |  | HMDB36397 |  | C18 |
| Lablabsaponin I | C54 H82 O22 | 1.392 | 161842-85-9 | HMDB41042 |  | C18 |
| LacCer(d18:0/16:0) | C46 H89 N O13 | 6.039 |  |  | LMSP0501AB14 | C18 |
| LacCer(d18:1/12:0) | C42 H79 N O13 | 4.943 |  |  | LMSP0501AB02 | C18 |
| LacCer(d18:1/14:0) | C44 H83 N O13 | 2.737 |  |  |  | HILIC |
| L-Alanine | C3 H7 N O2 | 0.366 | 56-41-7 | HMDB00161 |  | C18 |
| Landomycin D | C31 H34 O12 | 6.339 |  |  |  | C18 |
| Lansiol | C33 H56 O | 6.453 | 62462-35-5 | HMDB29587 |  | HILIC |
| Leu Pro Asn | C15 H26 N4 O5 | 2.355 |  |  |  | C18 |
| Leukotriene C5 | C30 H45 N3 O9 S | 1.964 | 75207-09-9 | HMDB12993 |  | C18 |
| Leupeptin | C20 H38 N6 O4 | 0.514 | 55123-66-5 |  |  | C18 |
| Lignoceric acid | C24 H48 O2 | 5.008 | 557-59-5 |  | LMFA01010024 | C18 |
| Lignoceroyl-EA | C26 H53 N O2 | 4.994 |  |  | LMFA08040054 | C18 |
| Linoleamide | C18 H33 N O | 1.916 |  |  | LMFA08010008 | C18 |
| Lipoxin A4 | C20 H32 O5 | 2.786 | 89663-86-5 | HMDB04385 |  | C18 |
| L-Lactic acid | C3 H6 O3 | 0.394 | 79-33-4 | HMDB00190 |  | C18 |
| Lucidenic acid J | C27 H38 O8 | 1.014 |  | HMDB35700 |  | C18 |
| Lycodine | C16 H22 N2 | 1.018 | 20316-18-1 |  |  | HILIC |
| LysoPC(16:1) | C24 H48 N O7 P | 3.923 |  | HMDB10383 |  | HILIC |
| LysoPC(22:4) | C30 H54 N O7 P | 1.630 |  | HMDB10401 |  | C18 |
| LysoPC(22:6) | C30 H50 N O7 P | 1.351 |  | HMDB10404 |  | C18 |
| LysoPE(20:5) | C25 H42 N O7 P | 1.443 |  | HMDB11489 |  | C18 |
| LysoPE(22:6) | C27 H44 N O7 P | 1.386 |  | HMDB11496 |  | C18 |
| Maltotetradecaose | C84 H142 O71 | 4.982 |  | HMDB59950 |  | C18 |
| Malvidin 3-(6''-p-coumarylglucoside)-5-dimalonylglucoside | C44 H45 O25 | 1.959 |  |  | LMPK12010392 | C18 |
| Malvidin 3-glucoside-4-vinylphenol | C31 H29 O13 | 4.979 | 388089-39-2 | HMDB31968 |  | C18 |
| Meperidinic acid glucuronide | C19 H25 N O8 | 4.010 | 71177-38-3 |  |  | HILIC |
| Mesaconyl-CoA | C26 H35 N7 O19 P3 S | 5.574 |  | HMDB60124 |  | C18 |
| Methylitaconate | C6 H8 O4 | 0.926 |  |  |  | HILIC |
| Metrizamide | C18 H22 I3 N3 O8 | 5.728 | 31112-62-6 | HMDB15518 |  | C18 |
| MG(16:1) | C19 H36 O4 | 2.890 |  | HMDB11565 |  | C18 |
| MG(17:0) | C20 H40 O4 | 2.522 |  |  | LMGL01010002 | C18 |
| MG(20:2) | C23 H42 O4 | 5.824 |  | HMDB11544 |  | C18 |
| MG(20:3) | C23 H40 O4 | 5.086 |  | HMDB11576 |  | C18 |
| MGDG(20:5/18:3) | C47 H74 O10 | 5.655 |  |  | LMGL05010013 | C18 |
| Montbretin B | C53 H64 O32 | 5.731 |  |  | LMPK12112448 | C18 |
| Mucronine B | C28 H36 N4 O4 | 1.016 | 38840-26-5 |  |  | C18 |
| N-(2-hydroxy-octadecanoyl)-1-beta-(3'-sulfo)-glucosyl-sphing-4-enine | C42 H81 N O12 S | 4.716 |  |  |  | C18 |
| N-(2R-Hydroxydocosanoyl)-2S-amino-1,3S,4R-octadecanetriol | C40 H81 N O5 | 7.678 |  | HMDB35469 |  | C18 |
| N(6)-(Octanoyl)lysine | C14 H28 N2 O3 | 1.015 |  | HMDB11684 |  | C18 |
| N-(6-Oxo-6H-dibenzo[b,d]pyran-3-yl)methanesulfonamide | C14 H11 N O4 S | 1.545 |  |  |  | C18 |
| N-Acetyl-D-fucosamine | C8 H15 N O5 | 8.556 |  |  |  | HILIC |
| N-acyl capnines | C32 H63 N O5 S | 5.285 |  |  | LMSP00000001 | C18 |
| Naltrindole | C26 H26 N2 O3 | 0.997 | 111555-53-4 |  |  | C18 |
| Naphthyl-2-hydroxymethyl-succinyl CoA | C36 H48 N7 O20 P3 S | 1.401 |  |  |  | C18 |
| N-Desmethyltramadol | C15 H23 N O2 | 0.931 |  | HMDB61007 |  | C18 |
| Ne,Ne dimethyllysine | C8 H18 N2 O2 | 4.716 |  | HMDB13287 |  | C18 |
| Neocasomorphin (1-5) | C29 H41 N5 O9 | 1.411 |  | HMDB60144 |  | C18 |
| NeuAca2-3Galb-Cer(d18:1/16:0) | C51 H94 N2 O16 | 1.547 |  |  |  | C18 |
| NeuAca2-3Galb-Cer(d18:1/20:0) | C55 H102 N2 O16 | 1.959 |  |  |  | C18 |
| NeuGca2-3Galb1-4GlcNAcb1-3(Galb1-4GlcNAcb1-6)Galb1-4GlcNAcb1-3Galb1-4Glcb-Cer(d18:1/16:0) | C99 H173 N5 O52 | 3.847 |  |  |  | HILIC |
| Neurine | C5 H13 N O | 1.992 | 463-88-7 | HMDB31259 |  | C18 |
| N-Hexadecanoylpyrrolidine | C20 H39 N O | 2.936 | 70974-48-0 | HMDB32740 |  | C18 |
| N-hexadecanoyl-valine | C21 H41 N O3 | 3.265 | 45287-42-1 |  | LMFA08020120 | C18 |
| Nicarbazin | C13 H10 N4 O5 | 1.025 | 330-95-0 |  |  | HILIC |
| nicotinate beta-D-ribonucleotide | C11 H15 N O9 P | 0.419 |  | HMDB59646 |  | HILIC |
| N-Nitrosodiethylamine | C4 H10 N2 O | 0.603 | 55-18-5 |  |  | HILIC |
| Notoginsenoside I | C54 H92 O22 | 1.399 | 193977-08-1 | HMDB31371 |  | C18 |
| N-palmitoyl-D-sphingosyl-1-(2-aminoethyl)phosphonate | C36 H73 N2 O5 P | 6.369 |  |  | LMSP04000002 | C18 |
| N-pentadecanoyl-L-Homoserine lactone | C19 H35 N O3 | 4.527 | 182359-66-6 |  |  | C18 |
| O-Acetylcypholophine | C20 H28 N2 O4 | 0.411 | 26482-11-1 |  |  | HILIC |
| O-Acetylserine | C13 H21 N2 O7 P S | 5.661 |  | HMDB03011 |  | C18 |
| O-decanoyl-R-carnitine | C17 H33 N O4 | 3.688 |  | HMDB00651 | LMFA07070006 | HILIC |
| Oleamide | C18 H35 N O | 4.285 | 301-02-0 |  |  | C18 |
| Oligomycin D | C44 H72 O11 | 1.016 | 1404-59-7 |  |  | C18 |
| Ornithokinin | C48 H77 N15 O11 | 6.722 | 14042-92-3 | HMDB38224 |  | C18 |
| Oxalyl-CoA | C23 H36 N7 O19 P3 S | 5.976 |  |  |  | C18 |
| Oxethazaine | C28 H41 N3 O3 | 6.454 | 126-27-2 |  |  | C18 |
| PA(12:0/16:0) | C31 H61 O8 P | 3.558 |  |  | LMGP10010944 | C18 |
| PA(17:2) | C20 H37 O7 P | 0.971 |  |  | LMGP10050022 | HILIC |
| PA(20:2/22:0) | C45 H85 O8 P | 4.013 |  |  | LMGP10010582 | C18 |
| PA(21:0/0:0) | C24 H49 O7 P | 1.684 |  |  | LMGP10050032 | C18 |
| PA(21:0/22:0) | C46 H91 O8 P | 2.442 |  |  | LMGP10010697 | C18 |
| PA(22:0/22:2) | C47 H89 O8 P | 4.718 |  |  | LMGP10010722 | C18 |
| PA(22:1/22:2)) | C47 H87 O8 P | 4.163 |  |  | LMGP10010752 | C18 |
| PA(22:2) | C25 H47 O7 P | 6.704 |  |  | LMGP10050030 | HILIC |
| PA(O-16:0/12:0) | C31 H63 O7 P | 1.014 |  |  | LMGP10020002 | C18 |
| PA(O-20:0/16:0) | C39 H79 O7 P | 6.786 |  |  | LMGP10020072 | C18 |
| PA(O-20:0/21:0) | C44 H89 O7 P | 5.535 |  |  | LMGP10020066 | C18 |
| PA(O-20:0/22:6) | C45 H79 O7 P | 5.259 |  |  | LMGP10020070 | C18 |
| PA(P-20:0/0:0) | C23 H47 O6 P | 3.266 |  |  | LMGP10070001 | C18 |
| PA(P-20:0/22:4) | C45 H81 O7 P | 5.792 |  |  | LMGP10030085 | C18 |
| Palmityl Trifluoromethyl Ketone | C17 H31 F3 O | 3.257 | 141022-99-3 |  |  | C18 |
| Palustrine | C17 H31 N3 O2 | 3.560 | 22324-44-3 |  |  | C18 |
| Paraquat dichloride | C12 H14 Cl2 N2 | 6.455 | 1910-42-5 | HMDB33121 |  | HILIC |
| Patuletin 3-(6''-p-coumaroylglucoside) | C31 H28 O15 | 6.908 |  |  | LMPK12112959 | HILIC |
| PC(14:0/20:4) | C42 H76 N O8 P | 5.009 |  |  | LMGP01012128 | C18 |
| PC(15:0/18:2) | C41 H78 N O8 P | 4.869 |  | HMDB07940 |  | C18 |
| PC(16:0) | C24 H50 N O7 P | 1.541 |  | HMDB10382 | LMGP01050018 | C18 |
| PC(16:0/20:3) | C44 H82 N O8 P | 5.117 |  | HMDB07980 |  | C18 |
| PC(16:0/9:0(CHO)) | C33 H64 N O9 P | 1.879 |  |  | LMGP20010008 | C18 |
| PC(16:1/20:1)) | C44 H84 N O8 P | 5.823 |  | HMDB08011 |  | C18 |
| PC(18:0) | C26 H54 N O7 P | 1.959 |  | HMDB10384 | LMGP01050026 | C18 |
| PC(18:1/18:3)) | C44 H80 N O8 P | 5.080 |  |  | LMGP01012151 | C18 |
| PC(18:1/20:2) | C46 H86 N O8 P | 6.013 |  |  | LMGP01012153 | C18 |
| PC(18:1/22:5) | C48 H84 N O8 P | 2.747 |  | HMDB08088 |  | HILIC |
| PC(18:2(9Z,12Z)/18:2(9Z,12Z)) | C44 H80 N O8 P | 5.133 |  |  | LMGP01012169 | C18 |
| PC(18:2/15:0) | C41 H78 N O8 P | 4.892 |  | HMDB08132 |  | C18 |
| PC(18:2/18:1) | C44 H82 N O8 P | 5.117 |  | HMDB08136 |  | C18 |
| PC(18:3/18:0) | C44 H82 N O8 P | 5.358 |  | HMDB08168 |  | C18 |
| PC(18:4(6Z,9Z,12Z,15Z)/20:4(5Z,8Z,11Z,14Z)) | C46 H76 N O8 P | 4.658 |  | HMDB08246 |  | C18 |
| PC(20:3(5Z,8Z,11Z)/16:0) | C44 H82 N O8 P | 5.118 |  |  | LMGP01012194 | C18 |
| PC(20:3(5Z,8Z,11Z)/20:4(8Z,11Z,14Z,17Z)) | C48 H82 N O8 P | 5.723 |  | HMDB08379 |  | C18 |
| PC(20:3(5Z,8Z,11Z)/22:4(7Z,10Z,13Z,16Z)) | C50 H86 N O8 P | 6.204 |  | HMDB08384 |  | C18 |
| PC(20:3(8Z,11Z,14Z)/20:4(5Z,8Z,11Z,14Z)) | C48 H82 N O8 P | 5.220 |  | HMDB08410 |  | C18 |
| PC(20:3/20:5) | C48 H80 N O8 P | 4.584 |  | HMDB08412 |  | C18 |
| PC(20:3/20:3) | C48 H84 N O8 P | 5.572 |  | HMDB08377 |  | C18 |
| PC(20:4(8Z,11Z,14Z,17Z)/16:0) | C44 H80 N O8 P | 5.085 |  | HMDB08462 |  | C18 |
| PC(20:4/22:5) | C50 H82 N O8 P | 5.572 |  | HMDB08483 |  | C18 |
| PC(20:5() | C28 H48 N O7 P | 3.860 |  | HMDB10397 | LMGP01050050 | HILIC |
| PC(20:5(5Z,8Z,11Z,14Z,17Z)/0:0) | C28 H48 N O7 P | 1.400 |  | HMDB10397 | LMGP01050050 | C18 |
| PC(20:5/18:1) | C46 H80 N O8 P | 4.979 |  |  | LMGP01012216 | C18 |
| PC(20:5/22:5) | C50 H80 N O8 P | 5.010 |  | HMDB08516 |  | C18 |
| PC(20:5/P-16:0) | C44 H78 N O7 P | 5.508 |  | HMDB08521 |  | C18 |
| PC(22:2(13Z,16Z)/P-18:1(9Z)) | C48 H90 N O7 P | 6.904 |  | HMDB08622 |  | C18 |
| PC(22:4/P-18:1) | C48 H86 N O7 P | 6.020 |  | HMDB08654 |  | C18 |
| PC(22:5(7Z,10Z,13Z,16Z,19Z)/P-18:1(11Z)) | C48 H84 N O7 P | 6.213 |  | HMDB08720 |  | C18 |
| PC(22:6/22:5) | C52 H82 N O8 P | 6.367 |  | HMDB08746 |  | C18 |
| PC(34:2) | C42 H80 N O8 P | 5.167 |  | HMDB07880 |  | C18 |
| PC(36:4) | C44 H80 N O8 P | 5.085 |  | HMDB07889 |  | C18 |
| PC(O-16:0/22:6(4Z,7Z,10Z,13Z,16Z,19Z)) | C46 H82 N O7 P | 5.467 |  | HMDB13409 | LMGP01020064 | C18 |
| PC(O-18:0/20:4(5E,8E,11E,14E)) | C46 H86 N O7 P | 6.215 |  |  | LMGP01020100 | C18 |
| PC(O-20:0/18:4(6Z,9Z,12Z,15Z)) | C46 H86 N O7 P | 4.716 |  |  | LMGP01020231 | C18 |
| PC(P-16:0/12:0) | C36 H72 N O7 P | 2.915 |  |  | LMGP01030019 | C18 |
| PC(P-18:0/22:6) | C48 H84 N O7 P | 5.448 |  | HMDB11262 |  | C18 |
| PC(P-18:1(11Z)/18:4(6Z,9Z,12Z,15Z)) | C44 H78 N O7 P | 5.508 |  | HMDB11280 |  | C18 |
| PC(P-18:1(9Z)/0:0) | C26 H52 N O6 P | 1.776 |  | HMDB10408 | LMGP01070012 | C18 |
| PC(P-20:0/20:5(5Z,8Z,11Z,14Z,17Z)) | C48 H86 N O7 P | 6.007 |  |  | LMGP01030098 | C18 |
| PC(P-20:0/22:4(7Z,10Z,13Z,16Z)) | C50 H92 N O7 P | 6.871 |  |  | LMGP01030103 | C18 |
| PE(14:0/16:0) | C35 H70 N O8 P | 4.992 |  | HMDB08824 |  | C18 |
| PE(15:0/18:0) | C38 H76 N O8 P | 4.903 |  | HMDB08892 |  | C18 |
| PE(15:1/20:0) | C40 H78 N O8 P | 5.018 |  |  | LMGP02010493 | C18 |
| PE(16:1(9Z)/19:0) | C40 H78 N O8 P | 5.019 |  |  | LMGP02010527 | C18 |
| PE(18:1(11Z)/22:2(13Z,16Z)) | C45 H84 N O8 P | 5.794 |  | HMDB09041 |  | C18 |
| PE(18:1(9Z)/16:1(9Z)) | C39 H74 N O8 P | 5.266 |  |  | LMGP02011198 | C18 |
| PE(18:1) | C23 H46 N O7 P | 5.388 |  | HMDB11506 | LMGP02050004 | C18 |
| PE(18:2(9Z,12Z)/20:1(11Z)) | C43 H80 N O8 P | 5.129 |  |  | LMGP02010670 | C18 |
| PE(18:3(6Z,9Z,12Z)/19:0) | C42 H78 N O8 P | 4.646 |  |  | LMGP02010696 | C18 |
| PE(18:3(9Z,12Z,15Z)/22:0) | C45 H84 N O8 P | 5.795 |  | HMDB09171 |  | C18 |
| PE(18:3(9Z,12Z,15Z)/P-18:1(11Z)) | C41 H74 N O7 P | 4.524 |  | HMDB09182 |  | C18 |
| PE(18:3/21:0) | C44 H82 N O8 P | 5.354 |  |  | LMGP02010704 | C18 |
| PE(19:0) | C24 H50 N O7 P | 3.908 |  |  | LMGP02050028 | HILIC |
| PE(19:0) | C24 H50 N O7 P | 3.908 |  |  | LMGP02050028 | HILIC |
| PE(19:1) | C24 H48 N O7 P | 1.319 |  |  | LMGP02050019 | C18 |
| PE(20:0/20:4(8Z,11Z,14Z,17Z)) | C45 H82 N O8 P | 5.359 |  | HMDB09235 |  | C18 |
| PE(20:2(11Z,14Z)/P-18:1(11Z)) | C43 H80 N O7 P | 5.656 |  | HMDB09314 |  | C18 |
| PE(20:4(5Z,8Z,11Z,14Z)/P-16:0) | C41 H74 N O7 P | 5.480 |  | HMDB09411 |  | C18 |
| PE(20:4)/21:0) | C46 H84 N O8 P | 5.731 |  |  | LMGP02010956 | C18 |
| PE(20:5(5Z,8Z,11Z,14Z,17Z)/P-18:1(11Z)) | C43 H74 N O7 P | 4.442 |  | HMDB09479 |  | C18 |
| PE(21:0/16:1) | C42 H82 N O8 P | 5.660 |  |  | LMGP02010995 | C18 |
| PE(22:1(11Z)/18:3(6Z,9Z,12Z)) | C45 H82 N O8 P | 5.429 |  |  | LMGP02011054 | C18 |
| PE(22:2(13Z,16Z)/15:1(9Z)) | C42 H78 N O8 P | 4.919 |  |  | LMGP02011076 | C18 |
| PE(22:2(13Z,16Z)/P-16:0) | C43 H82 N O7 P | 4.720 |  | HMDB09576 |  | C18 |
| PE(22:4(7Z,10Z,13Z,16Z)/15:1(9Z)) | C42 H74 N O8 P | 4.523 |  |  | LMGP02011107 | C18 |
| PE(22:4(7Z,10Z,13Z,16Z)/P-18:1(11Z)) | C45 H80 N O7 P | 5.381 |  | HMDB09611 |  | C18 |
| PE(22:5)/P-16:0) | C43 H76 N O7 P | 5.550 |  | HMDB09675 |  | C18 |
| PE(22:6(4Z,7Z,10Z,13Z,16Z,19Z)/17:1(9Z)) | C44 H74 N O8 P | 4.440 |  |  | LMGP02011140 | C18 |
| PE(22:6(4Z,7Z,10Z,13Z,16Z,19Z)/19:1(9Z)) | C46 H78 N O8 P | 4.366 |  |  | LMGP02011149 | C18 |
| PE(22:6(4Z,7Z,10Z,13Z,16Z,19Z)/21:0) | C48 H84 N O8 P | 5.574 |  |  | LMGP02011156 | C18 |
| PE(22:6/P-16:0) | C43 H74 N O7 P | 5.325 |  | HMDB09708 |  | C18 |
| PE(O-16:0/12:0) | C33 H68 N O7 P | 1.014 |  |  | LMGP02020021 | C18 |
| PE(O-16:0/13:0) | C34 H70 N O7 P | 2.420 |  |  | LMGP02020022 | C18 |
| PE(O-16:0/17:1(9Z)) | C38 H76 N O7 P | 3.265 |  |  | LMGP02020028 | C18 |
| PE(O-16:0/22:5(4Z,7Z,10Z,13Z,16Z)) | C43 H78 N O7 P | 5.217 |  |  | LMGP02020006 | C18 |
| PE(P-16:0/0:0) | C21 H44 N O6 P | 1.767 |  |  | LMGP02070001 | C18 |
| PE(P-16:0/20:4(6E,8Z,11Z,14Z)(5OH[S])) | C41 H74 N O8 P | 5.197 |  |  | LMGP20020011 | C18 |
| PE(P-18:1/20:4(12OH[S])) | C43 H76 N O8 P | 4.869 |  |  | LMGP20020006 | C18 |
| PE-Cer(d14:1/23:0) | C39 H79 N2 O6 P | 4.701 |  |  | LMSP03020010 | C18 |
| Pentacosanyl oleate | C41 H80 O2 | 1.015 |  |  | LMFA07010029 | C18 |
| Petunidin | C16 H13 Cl O7 | 4.722 | 1429-30-7 | HMDB03173 |  | C18 |
| Petunidin 3,7-di-(6-malonylglucoside) | C34 H37 O23 | 6.561 |  |  | LMPK12010367 | C18 |
| PG(12:0/18:0) | C36 H71 O10 P | 2.896 |  |  | LMGP04010942 | C18 |
| PG(15:1(9Z)/14:1(9Z)) | C35 H65 O10 P | 1.014 |  |  | LMGP04010164 | C18 |
| PG(19:1(9Z)/22:6(4Z,7Z,10Z,13Z,16Z,19Z)) | C47 H79 O10 P | 4.099 |  |  | LMGP04010510 | C18 |
| PG(20:4/22:6) | C48 H75 O10 P | 3.845 |  |  | LMGP04010044 | HILIC |
| PG(P-20:0/0:0) | C26 H53 O8 P | 1.385 |  |  | LMGP04070001 | C18 |
| PGF2α isopropyl ester | C23 H40 O5 | 1.563 | 53764-90-2 |  | LMFA03010076 | C18 |
| PGP(18:0/18:1(9Z)) | C42 H82 O13 P2 | 4.100 |  | HMDB13506 |  | C18 |
| Phalluside-1 | C41 H75 N O9 | 5.324 |  |  | LMSP05010039 | C18 |
| Phosphoric acid | H3 O4 P | 4.133 | 7664-38-2 | HMDB02142 |  | HILIC |
| Phyllanthusol B | C35 H49 N O18 | 5.219 | 294864-89-4 | HMDB35904 |  | C18 |
| PI(12:0/21:0) | C42 H81 O13 P | 5.876 |  |  | LMGP06010032 | C18 |
| PI(16:0/18:1(9Z)) | C43 H81 O13 P | 4.569 |  | HMDB09783 | LMGP06010001 | C18 |
| PI(16:0/20:5(5Z,8Z,11Z,14Z,17Z)) | C45 H77 O13 P | 4.098 |  |  | LMGP06010874 | C18 |
| PI(18:1(9Z)/16:1(9Z)) | C43 H79 O13 P | 4.098 |  | HMDB09835 |  | C18 |
| PI(18:1(9Z)/18:1(11Z)) | C45 H83 O13 P | 4.763 |  | HMDB09836 |  | C18 |
| PI(18:1(9Z)/18:3(6Z,9Z,12Z)) | C45 H79 O13 P | 4.032 |  |  | LMGP06010298 | C18 |
| PI(18:1/19:0) | C46 H87 O13 P | 6.364 |  |  | LMGP06010300 | C18 |
| PI(18:2/22:6) | C49 H79 O13 P | 5.578 |  |  | LMGP06010844 | C18 |
| PI(18:3/20:4) | C47 H77 O13 P | 4.031 |  |  | LMGP06010357 | C18 |
| PI(18:3/20:5) | C47 H75 O13 P | 4.101 |  |  | LMGP06010358 | C18 |
| PI(18:3/22:6) | C49 H77 O13 P | 3.909 |  |  | LMGP06010364 | C18 |
| PI(18:3(9Z,12Z,15Z)/19:0) | C46 H83 O13 P | 4.989 |  |  | LMGP06010379 | C18 |
| PI(18:3(9Z,12Z,15Z)/22:4(7Z,10Z,13Z,16Z)) | C49 H81 O13 P | 4.616 |  |  | LMGP06010391 | C18 |
| PI(18:4(6Z,9Z,12Z,15Z)/16:0) | C43 H75 O13 P | 3.945 |  |  | LMGP06010399 | C18 |
| PI(19:1(9Z)/13:0) | C41 H77 O13 P | 3.944 |  |  | LMGP06010449 | C18 |
| PI(19:1(9Z)/22:0) | C50 H95 O13 P | 1.463 |  |  | LMGP06010474 | C18 |
| PI(20:0/19:0) | C48 H93 O13 P | 6.392 |  |  | LMGP06010488 | C18 |
| PI(20:1) | C29 H55 O12 P | 6.782 |  |  | LMGP06050019 | HILIC |
| PI(20:3/16:0) | C45 H81 O13 P | 4.282 |  | HMDB09886 |  | C18 |
| PI(20:4(5Z,8Z,11Z,14Z)/18:3(6Z,9Z,12Z)) | C47 H77 O13 P | 4.033 |  |  | LMGP06010601 | C18 |
| PI(20:4(5Z,8Z,11Z,14Z)/18:3(9Z,12Z,15Z)) | C47 H77 O13 P | 4.032 |  |  | LMGP06010602 | C18 |
| PI(20:4) | C29 H49 O12 P | 1.169 |  |  | LMGP06050006 | C18 |
| PI(20:5(5Z,8Z,11Z,14Z,17Z)/0:0) | C29 H47 O12 P | 1.174 |  |  | LMGP06050026 | C18 |
| PI(21:0/20:2(11Z,14Z)) | C50 H93 O13 P | 1.013 |  |  | LMGP06010663 | C18 |
| PI(22:1(11Z)/22:0) | C53 H101 O13 P | 1.540 |  |  | LMGP06010720 | C18 |
| PI(22:4(7Z,10Z,13Z,16Z)/22:2(13Z,16Z)) | C53 H91 O13 P | 1.463 |  |  | LMGP06010784 | C18 |
| PI(22:6/16:0) | C47 H79 O13 P | 4.262 |  |  | LMGP06010815 | C18 |
| PI(O-20:0/12:0) | C41 H81 O12 P | 4.162 |  |  | LMGP06020042 | C18 |
| PI(O-20:0/22:2(13Z,16Z)) | C51 H97 O12 P | 6.702 |  |  | LMGP06020068 | C18 |
| PI(P-18:0/14:1(9Z)) | C41 H77 O12 P | 1.014 |  |  | LMGP06030032 | C18 |
| PI(P-20:0/21:0) | C50 H97 O12 P | 6.909 |  |  | LMGP06030082 | C18 |
| Pinocembrin 7-O-neohesperidoside 3'''-O-acetate | C29 H34 O14 | 5.807 |  |  | LMPK12140140 | C18 |
| PIP(18:1/18:0) | C45 H86 O16 P2 | 5.946 |  | HMDB09972 |  | C18 |
| Piperidine | C5 H11 N | 0.391 | 110-89-4 | HMDB34301 |  | C18 |
| Polyethylene, oxidized | C12 H20 O5 | 6.350 | 68441-17-8 | HMDB32472 |  | HILIC |
| Procaterol | C16 H22 N2 O3 | 0.733 | 59828-07-8 |  |  | C18 |
| Prosopinine | C16 H33 N O3 | 4.100 |  |  | LMSP01080049 | C18 |
| Prostaglandin E2 p-acetamidophenyl ester | C28 H39 N O6 | 1.015 | 57790-52-0 |  |  | C18 |
| PS(16:1/22:0) | C44 H84 N O10 P | 5.437 |  |  | LMGP03010222 | C18 |
| PS(17:1/22:6) | C45 H74 N O10 P | 5.085 |  |  | LMGP03010277 | C18 |
| PS(19:0/0:0) | C25 H50 N O9 P | 1.320 |  |  | LMGP03050028 | C18 |
| PS(19:1(9Z)/16:1(9Z)) | C41 H76 N O10 P | 4.377 |  |  | LMGP03010487 | C18 |
| PS(19:1(9Z)/22:4(7Z,10Z,13Z,16Z)) | C47 H82 N O10 P | 4.946 |  |  | LMGP03010509 | C18 |
| PS(19:1/20:5) | C45 H76 N O10 P | 5.370 |  |  | LMGP03010504 | C18 |
| PS(20:0/20:0) | C46 H90 N O10 P | 6.230 |  |  | LMGP03010863 | C18 |
| PS(21:0/20:4) | C47 H84 N O10 P | 5.939 |  |  | LMGP03010696 | C18 |
| PS(21:0/22:6) | C49 H84 N O10 P | 6.363 |  |  | LMGP03010004 | C18 |
| PS(22:1/17:1) | C45 H84 N O10 P | 5.422 |  |  | LMGP03010734 | C18 |
| PS(22:2(13Z,16Z)/20:0) | C48 H90 N O10 P | 6.868 |  |  | LMGP03010775 | C18 |
| PS(22:6/21:0) | C49 H84 N O10 P | 5.759 |  |  | LMGP03010841 | C18 |
| PS(22:6/19:0) | C47 H80 N O10 P | 5.040 |  |  | LMGP03010833 | C18 |
| PS(O-18:0/0:0) | C24 H50 N O8 P | 1.316 |  |  | LMGP03060002 | C18 |
| PS(O-20:0/14:1(9Z)) | C40 H78 N O9 P | 5.258 |  |  | LMGP03020044 | C18 |
| PS(P-18:0/18:3(6Z,9Z,12Z)) | C42 H76 N O9 P | 5.218 |  |  | LMGP03030041 | C18 |
| PS(P-20:0/18:4(6Z,9Z,12Z,15Z)) | C44 H78 N O9 P | 4.379 |  |  | LMGP03030072 | C18 |
| PS(P-20:0/22:2(13Z,16Z)) | C48 H90 N O9 P | 6.621 |  |  | LMGP03030084 | C18 |
| PS(P-20:0/22:6) | C48 H82 N O9 P | 5.935 |  |  | LMGP03030093 | C18 |
| PtdIns-(1,2-dihexanoyl) | C21 H39 O13 P | 1.108 |  |  |  | C18 |
| Punicalin | C68 H44 O44 | 5.085 | 65995-64-4 | HMDB39205 |  | C18 |
| Purpureacin-1 | C37 H66 O8 | 1.015 | 150134-21-7 |  |  | C18 |
| Quercetin 3-(2''-galloyl-alpha-L-arabinopyranoside) | C27 H22 O15 | 6.448 |  |  | LMPK12112212 | HILIC |
| Quercetin 3-glucuronide-7-rutinoside | C33 H38 O22 | 5.830 |  |  | LMPK12112210 | C18 |
| Rescinnamine | C35 H42 N2 O9 | 1.373 | 24815-24-5 | HMDB15311 |  | C18 |
| Resolvin D1 | C22 H32 O5 | 2.417 |  | HMDB03733 |  | C18 |
| Rhazidigenine Nb-oxide | C19 H26 N2 O2 | 3.791 | 26066-43-3 | HMDB30263 |  | C18 |
| Rhodanine | C3 H3 N O S2 | 0.330 | 141-84-4 |  |  | C18 |
| Ribose 1,5-bisphosphate | C5 H12 O11 P2 | 3.843 | 14689-84-0 | HMDB11688 |  | HILIC |
| S-(Indolylmethylthiohydroximoyl)-L-cysteine | C13 H15 N3 O3 S | 6.780 |  |  |  | HILIC |
| Sabinene hydrate | C10 H18 O | 2.186 |  |  |  | C18 |
| Salannin | C34 H44 O9 | 1.176 | 992-20-1 |  |  | C18 |
| Salicin 6-phosphate | C13 H19 O10 P | 0.577 |  |  |  | C18 |
| Sanguiin H3 | C68 H48 O44 | 5.117 | 82262-86-0 | HMDB39257 |  | C18 |
| Sarcoaldesterol A | C30 H52 O4 | 3.555 |  |  |  | C18 |
| Sclerocitrin | C36 H22 O17 | 4.786 |  |  |  | C18 |
| SDZ PSC 833 | C63 H111 N11 O12 | 5.088 | 121584-18-7 |  |  | C18 |
| Ser Asn Asn | C11 H19 N5 O7 | 3.844 |  |  |  | HILIC |
| Ser Ser Lys | C12 H24 N4 O6 | 0.535 |  |  |  | HILIC |
| Siroheme | C42 H44 Fe N4 O16 | 6.901 |  |  |  | HILIC |
| SK&F 91581 | C8 H14 N4 S | 0.922 | 34970-65-5 |  |  | HILIC |
| SM(d16:1/18:0) | C39 H79 N2 O6 P | 4.719 |  |  | LMSP03010042 | C18 |
| SM(d18:1/16:0) | C39 H79 N2 O6 P | 4.709 |  |  | LMSP03010003 | C18 |
| SM(d18:1/17:0) | C40 H81 N2 O6 P | 5.005 |  |  | LMSP03010044 | C18 |
| SM(d18:1/20:1) | C43 H85 N2 O6 P | 5.513 |  |  | LMSP03010059 | C18 |
| SM(d18:1/23:0) | C46 H93 N2 O6 P | 6.892 |  |  | LMSP03010078 | C18 |
| SM(d18:1/24:0) | C47 H95 N2 O6 P | 6.297 |  |  | LMSP03010008 | C18 |
| SM(d18:2/23:0) | C46 H91 N2 O6 P | 6.436 |  |  | LMSP03010075 | C18 |
| SM(d18:2/24:0) | C47 H93 N2 O6 P | 6.565 |  |  | LMSP03010081 | C18 |
| SM(d19:1/16:0) | C40 H81 N2 O6 P | 5.007 |  |  | LMSP03010045 | C18 |
| S-Methyl benzenecarbothioate | C8 H8 O S | 10.552 | 5925-68-8 | HMDB29694 |  | C18 |
| Smilagenin 3-[2''-glucosyl-6''-arabinosylglucoside] | C44 H72 O17 | 4.100 | 244762-26-3 | HMDB34309 |  | C18 |
| Sophoracoumestan B | C17 H10 O7 | 3.983 |  |  | LMPK12090037 | HILIC |
| Sphing-4-enine-1-phosphocholine | C23 H49 N2 O5 P | 2.915 |  |  | LMSP01060001 | C18 |
| Sphinganine-1-phosphate | C18 H40 N O5 P | 4.161 |  | HMDB01383 | LMSP01050002 | C18 |
| Sterculic acid | C19 H34 O2 | 1.960 |  |  | LMFA01140018 | C18 |
| Succinylcholine | C14 H30 N2 O4 | 0.334 | 306-40-1 | HMDB14347 |  | HILIC |
| Sulfathalidine | C17 H13 N3 O5 S2 | 4.987 | 85-73-4 |  |  | C18 |
| Synaptolepis factor K1 | C36 H54 O8 | 3.559 | 66268-94-8 |  |  | C18 |
| Tellimagrandin I | C34 H26 O22 | 5.823 | 79786-08-6 |  |  | C18 |
| Tetracaine N-oxide | C15 H24 N2 O3 | 0.510 | 55750-02-2 |  |  | C18 |
| Tetracenomycin F2 | C20 H16 O8 | 0.353 |  |  |  | C18 |
| Tetrahydrocortisol | C21 H34 O5 | 3.158 |  | HMDB00949 | LMST02030143 | C18 |
| Tetrahydrodipicolinate | C7 H9 N O4 | 6.987 | 2353-17-5 | HMDB12289 |  | HILIC |
| Tetronasin | C35 H53 O8 | 1.366 | 75139-06-9 | HMDB33494 |  | C18 |
| TG(12:0/12:0/12:0) | C39 H74 O6 | 6.780 |  | HMDB11188 |  | C18 |
| TG(12:0/20:5/22:6) | C57 H88 O6 | 6.910 |  |  | LMGL03013643 | C18 |
| TG(14:0/14:0/14:0) | C45 H86 O6 | 4.720 |  |  | LMGL03012616 | C18 |
| TG(16:0/20:4/o-18:0) | C57 H104 O5 | 3.156 |  | HMDB44556 |  | C18 |
| TG(16:0/22:0/16:1) | C57 H108 O6 | 3.265 |  | HMDB43975 |  | C18 |
| TG(16:1/16:0/20:4) | C55 H96 O6 | 8.375 |  | HMDB48468 |  | C18 |
| TG(16:1(9Z)/20:0/16:1) | C55 H102 O6 | 9.431 |  | HMDB48501 |  | C18 |
| TG(16:1/20:4/18:2) | C58 H98 O6 | 1.014 |  | HMDB48970 |  | C18 |
| TG(17:2/18:3/18:4) | C56 H90 O6 | 6.560 |  |  | LMGL03016076 | C18 |
| TG(17:2/18:3/22:5) | C60 H96 O6 | 2.911 |  |  | LMGL03011303 | C18 |
| TG(18:1/14:0/18:3) | C53 H94 O6 | 8.971 |  | HMDB49707 |  | C18 |
| TG(18:1/14:0/18:2) | C53 H96 O6 | 8.620 |  | HMDB10436 |  | C18 |
| TG(18:2/15:0/18:4) | C55 H94 O6 | 8.620 |  | HMDB52420 |  | C18 |
| TG(18:2/22:6/22:6) | C66 H100 O6 | 2.909 |  | HMDB52829 |  | C18 |
| TG(18:4/18:4/18:4) | C57 H86 O6 | 6.441 |  |  | LMGL03012624 | C18 |
| TG(20:0/14:1/18:3) | C55 H98 O6 | 9.444 |  | HMDB45671 |  | C18 |
| TG(20:1/14:0/18:4) | C55 H96 O6 | 8.980 |  | HMDB50313 |  | C18 |
| TG(20:1/14:0/o-18:0) | C55 H106 O5 | 4.988 |  |  |  | C18 |
| TG(22:1/14:1/20:4) | C59 H102 O6 | 9.446 |  | HMDB51533 |  | C18 |
| TG(22:4/16:0/18:3) | C59 H100 O6 | 9.010 |  | HMDB54660 |  | C18 |
| TG(24:0/24:0/18:2) | C70 H132 O6 | 1.962 |  | HMDB47158 |  | C18 |
| Thiobinupharidine | C30 H42 N2 O2 S | 3.533 | 30343-72-7 |  |  | C18 |
| Thornasterol A | C27 H44 O4 | 16.877 |  |  | LMST01010319 | HILIC |
| Thr Pro Asn | C13 H22 N4 O6 | 0.533 |  |  |  | C18 |
| Thr Pro Asn | C13 H22 N4 O6 | 1.289 |  |  |  | HILIC |
| Thr Ser Ser | C10 H19 N3 O7 | 0.868 |  |  |  | HILIC |
| Thr-Met-OH | C15 H20 N2 O7 S | 5.087 |  |  |  | C18 |
| Tin chloride (SnCl4) | Cl4 Sn | 1.400 | 7646-78-8 | HMDB36546 |  | C18 |
| trans-2-Dodecenoylcarnitine | C19 H35 N O4 | 3.584 |  | HMDB13326 |  | HILIC |
| trans-9-palmitoleic acid | C16 H30 O2 | 2.253 |  |  | LMFA01030057 | C18 |
| Tridecanoylglycine | C15 H29 N O3 | 0.793 |  | HMDB13317 |  | C18 |
| Trihexosylceramide (d18:1/16:0) | C52 H97 N O18 | 4.449 | 71965-57-6 | HMDB04879 |  | C18 |
| Trimethoprim | C14 H18 N4 O3 | 3.917 | 738-70-5 |  |  | HILIC |
| Trimetrexate | C19 H23 N5 O3 | 5.660 | 52128-35-5 |  |  | C18 |
| Troglitazone glucuronide | C30 H35 N O11 S | 5.909 | 127040-01-1 |  |  | C18 |
| Tropine | C8 H15 N O | 4.470 | 120-29-6 |  |  | HILIC |
| Tubocurarine | C37 H41 N2 O6 | 1.414 | 6989-98-6 | HMDB15330 |  | C18 |
| Tyr Gly Cys | C14 H19 N3 O5 S | 0.558 |  |  |  | HILIC |
| Tyr Tyr Phe | C27 H29 N3 O6 | 0.525 |  |  |  | C18 |
| Ubiquinol 8 | C49 H78 O4 | 3.644 | 56275-39-9 | HMDB01060 |  | HILIC |
| Ubiquinol-10 | C59 H92 O4 | 6.562 | 5677-55-4 | HMDB13111 |  | C18 |
| UDP-2,3-diacetamido-2,3-dideoxy-alpha-D-glucuronate | C19 H28 N4 O18 P2 | 6.792 |  |  |  | HILIC |
| UDP-L-Ara4O | C14 H20 N2 O16 P2 | 0.419 |  |  |  | HILIC |
| UDP-N-acetyl-D-galactosamine 4,6-bissulfate | C17 H27 N3 O23 P2 S2 | 1.546 |  |  |  | C18 |
| Undecaprenyl diphosphate | C55 H92 O7 P2 | 1.587 | 23-13-2 | HMDB01469 |  | C18 |
| Veratrine | C32 H49 N O9 | 1.504 | 62-59-9 |  |  | C18 |
| Vernolide | C19 H22 O7 | 0.563 | 27428-86-0 |  |  | C18 |
| Xanthophyll | C40 H56 O2 | 5.218 | 127-40-2 | HMDB03233 |  | C18 |
| Zeaxanthin | C40 H56 O2 | 3.294 | 144-68-3 | HMDB02789 |  | C18 |
| zeta-Carotene | C40 H60 | 2.782 |  |  | LMPR01070256 | C18 |
| Z-Gly-Pro-Leu-Gly-Pro | C28 H39 N5 O8 | 1.564 | 2646-61-9 |  |  | C18 |
| β-Carotene 5,6-epoxide | C40 H56 O | 6.931 |  |  | LMPR01070267 | HILIC |
